# Parallel Evolution of HIV-1 in a Long-Term Experiment

**DOI:** 10.1093/molbev/msz155

**Published:** 2019-07-04

**Authors:** Frederic Bertels, Christine Leemann, Karin J Metzner, Roland R Regoes

**Affiliations:** 1 Department of Environmental Systems Sciences, Institute of Integrative Biology, ETH Zurich, Zurich, Switzerland; 2 Department of Microbial Population Biology, Max-Planck-Institute for Evolutionary Biology, Plön, Germany; 3 Division of Infectious Diseases and Hospital Epidemiology, University Hospital Zurich, Zurich, Switzerland; 4 Insitute of Medical Virology, University of Zurich, Zurich, Switzerland

**Keywords:** evolution experiment, parallel evolution, predicting evolution, virus evolution, HIV-1

## Abstract

One of the most intriguing puzzles in biology is the degree to which evolution is repeatable. The repeatability of evolution, or parallel evolution, has been studied in a variety of model systems, but has rarely been investigated with clinically relevant viruses. To investigate parallel evolution of HIV-1, we passaged two replicate HIV-1 populations for almost 1 year in each of two human T-cell lines. For each of the four evolution lines, we determined the genetic composition of the viral population at nine time points by deep sequencing the entire genome. Mutations that were carried by the majority of the viral population accumulated continuously over 1 year in each evolution line. Many majority mutations appeared in more than one evolution line, that is, our experiments showed an extreme degree of parallel evolution. In one of the evolution lines, 62% of the majority mutations also occur in another line. The parallelism impairs our ability to reconstruct the evolutionary history by phylogenetic methods. We show that one can infer the correct phylogenetic topology by including minority mutations in our analysis. We also find that mutation diversity at the beginning of the experiment is predictive of the frequency of majority mutations at the end of the experiment.

## Introduction

In long-term evolution experiments, organisms are passaged for many generations in controlled environments in order to understand basic evolutionary principles (Elena and Lenski 2003). One of the most basic questions addressed with experimental evolution is to what extent the evolutionary path of an organism is predetermined, and hence can be predicted and repeated. Stephen J. Gould (1989) famously claimed, “[…] any replay of the tape of life would lead evolution down a pathway radically different from the one actually taken […]” thus rejecting the idea of evolution as a repeatable and predictable process. According to Gould, any deviation from a common evolutionary path will lead to different evolutionary end points. This view is consistent with the neutral theory of evolution (Kimura 1983), according to which genetic changes accumulate mostly randomly without conferring fitness advantages or disadvantages.

Gould’s hypothesis has been experimentally tested numerous times by letting populations of organisms evolve repeatedly in the same environment. In such experiments, the parallel evolution of similar traits and, in rare cases, also identical mutations, have been observed in bacteria and viruses (Bull et al. 1997; Wichman et al. 1999, 2005; Barrick et al. 2009; Engel et al. 2011; Lobkovsky and Koonin 2012; de Visser and Krug 2014; Foll et al. 2014; Bailey et al. 2015; Lind et al. 2015, 2018; Gallie et al. 2019).

Particularly, relevant experiments that study parallel evolution in viruses were performed by James Bull and Holly Wichman. In an early experiment phage, φX174 was grown on two different bacterial hosts, *Escherichia coli* and *Salmonella typhimurium* (Bull et al. 1997). After 11 days of growth in two and three replicates, respectively, the authors observed 42% of all mutations in more than one replicate line. Due to the extensive parallelism, phylogenetic inference methods, applied to the final sequence data, failed to recover the known evolutionary history of the phage populations. Ever since, multiple follow-up experiments have been performed with other phages exploring convergent and parallel evolution (Wichman et al. 1999, 2000, 2005; Miller et al. 2016).

There are also examples of experimental evolution studies with clinically relevant viruses. Experimental evolution has been applied to answer a wide range of question about the epidemiology and biology of these viruses. For example, studies have investigated: the effect of different drug concentrations on viral growth and evolution (Gatanaga et al. 2002; Maisnier-Patin and Andersson 2004); the effect of fitness recovery after bottlenecking (Lorenzo-Redondo et al. 2011); the effect of different environmental and genetic parameters on adaptation (Das et al. 1999; van Opijnen and Berkhout 2005; van Opijnen et al. 2007; Vasilakis et al. 2009; Bordería et al. 2015); and the shape of fitness landscapes (Acevedo et al. 2014; Lorenzo-Redondo et al. 2014). Hence, experimental evolution has been a valuable tool to understand the epidemiology and evolution of viruses.

Apart from the insights gained from a detailed analysis of a well-defined experiment, experimental studies of clinically relevant viruses can also take advantage of the vast amount of knowledge gained from clinical studies. Particularly, Human Immunodeficiency Virus (HIV) is one of the most relevant and deadly infections in the human population. In 2012, about 36.9 million people were infected with HIV (Joint United Nations Programme on HIV AIDS 2018). Due to its prevalence and a mortality rate of close to 100% if left untreated, HIV is extremely well studied. In PubMed alone, there are 185,490 articles with HIV in the title (May 2, 2019).

Part of the reason for the evolutionary success of HIV-1 in infecting the human population is its high mutation rate, which contributes to its ability to evade the human immune response and persist for years within the host (Coffin 1995; Koenig et al. 1995; Borrow et al. 1997; Goulder et al. 1997; Wei et al. 2003; Trkola et al. 2005; Liao et al. 2013). Over this time span, HIV-1 evolves and acquires a large number of mutations (Shankarappa et al. 1999; Lemey et al. 2006; Keele et al. 2008; Poon et al. 2010). The distribution and type of these mutations is not entirely random but converges between different lineages that evolve in similar hosts or are treated with similar drugs (Crandall et al. 1999; Duwe et al. 2001; Gatanaga et al. 2002; Lataillade et al. 2007). This convergence even allows the inference of the genetic setup of the human host from the viral sequence (Moore et al. 2002; Bhattacharya et al. 2007).

To study the extent of parallel evolution in a constant environment, we track the evolution of four independent HIV-1 populations for 315 days, which corresponds to about 180 generations (Srivastava et al. 1991; Ho et al. 1995; Wei et al. 1995; Mohammadi et al. 2013; Holmes et al. 2015). We identify all mutations that increase in frequency above that of the wild-type nucleotide, and compare mutations acquired in the four independent lineages. We observe a high degree of parallel evolution that causes phylogenetic reconstruction methods to infer an incorrect evolutionary history. This observation highlights the need to carefully assess the validity of phylogenetic methods that rely on the assumption of neutral evolution when analyzing within-host evolution of HIV-1, in particular phyloanatomic analyses (Salemi and Rife 2016; Lorenzo-Redondo et al. 2016; Bons and Regoes 2018). We also provide insights into how viral population sequencing data can be used to alleviate problems of phylogenetic reconstruction and analyze sequence diversity to predict evolutionary outcomes.

## Results

**Fig. 1.**
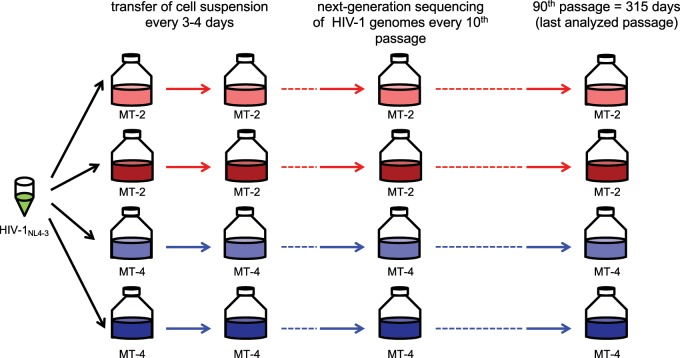
Experimental design. HIV-1 NL4-3 was serially transferred in MT-2 and MT-4 T-cells in two replicates each. Odd transfers occurred after 3 days, even transfers after 4 days. At each transfer, we infected new T-cells with ∼0.05% of the cell suspension from the previous culture (supplementary fig. S1, Supplementary Material online).

We investigated the long-term evolution of HIV-1 in the laboratory by passaging HIV-1 NL4-3 for 315 days in the absence of antiviral drugs in two different human T-cell cultures (MT-2 and MT-4), with two replicates on each T-cell culture (fig. 1). The viral populations were transferred to fresh culture two times every week (90 times in total), which should correspond to about 180 viral generations (Srivastava et al. 1991; Ho et al. 1995; Wei et al. 1995; Mohammadi et al. 2013; Holmes et al. 2015). Every tenth transfer, we determined the population composition by Illumina sequencing for each of the four evolution lines.

The HIV-1 population grows exponentially between transfers. At the start of each transfer, our cultures contain ∼400,000 uninfected T-cells. With a multiplicity of infection (MOI) of 0.0001–0.004 (supplementary fig. S1, Supplementary Material online), between 40 and 1,600 cells are infected during the first generation after each inoculation. The number of initially infected cells (i.e., 40–1,600) constitutes the population bottlenecks in our experiment. Since we transfer ∼0.05% of the previous culture into the new culture and we do not observe any obvious HIV-1 population growth or death across our experiment, we can assume that each HIV-1 population amplifies 2,000-fold between transfers (supplementary fig. S1, Supplementary Material online). There are two parameters that determine the level of population amplification in our experiment: the generation time and the number of infective viral offspring (*R*_0_). *R*_0_ of ∼10 (Ribeiro et al. 2010) and the generation time of ∼2 days (Srivastava et al. 1991; Ho et al. 1995; Wei et al. 1995; Mohammadi et al. 2013; Holmes et al. 2015) that are cited in the literature cannot explain the virus amplification in our experiment. These parameters would only allow for a 100-fold amplification of the virus population and hence would lead to the HIV-1 populations dying out in our experiment. To increase the population size 2,000-fold, we need to either assume *R*_0_ to be 44 while the HIV-1 population completes two generations or assume *R*_0_ to be 12 while the HIV-1 population completes three generations. The truth may lie somewhere in the middle. Either way we expect the MOI to reach a value close to one at the end of each transfer, which would allow for recombination to happen in our experiment.

### Majority Mutations Accumulate Linearly over Time

**Fig. 2.**
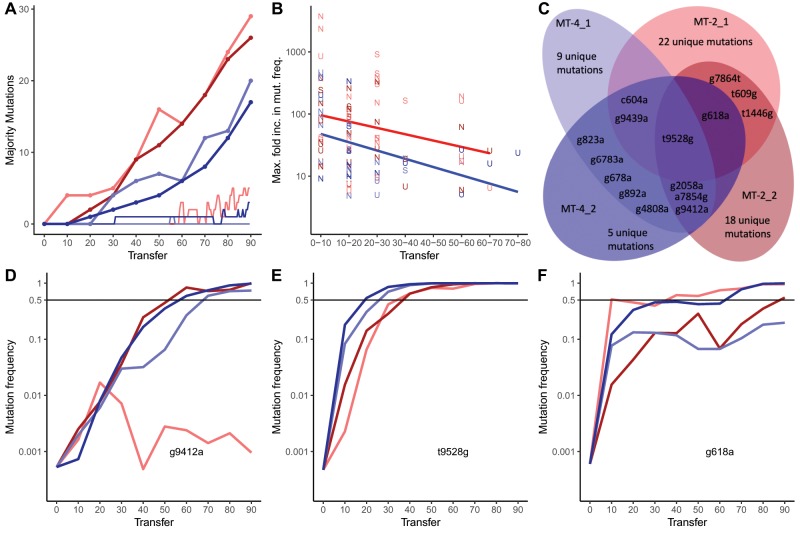
Accumulation, maximum mutation frequency slopes (fitness), parallelism, and dynamics of majority mutations. (*A*) Accumulation of majority mutations over time (thick lines), accumulation of neutral majority mutations in simulations over 180 generations (thin lines). (*B*) For each majority mutation observed in the evolution experiment, we show the maximum fold mutation frequency increase, plotted against the time frame at which the frequency increase occurred. The maximum fold increase of the mutation frequencies is a surrogate of the fitness advantage of a mutation. Synonymous mutations are displayed as “S,” nonsynonymous mutations are displayed as “N,” and mutations in untranslated regions are shown as “U.” Linear regressions on the fitness advantages in MT-2 and MT-4 T-cells are displayed in red and blue, respectively. The decline in fitness advantages for evolution lines grown in MT-2 (red) is marginally significant from 0 (*P* value = 0.05) and significantly different for MT-4 (blue) evolution lines (*P* value = 0.008). There is no majority mutation, for which the maximum mutation frequency increase occurred after transfer 80. (*C*) Venn diagram of the presence of majority mutations in the four evolution lines at transfer 90. (*D*–*F*) show mutation frequencies of three selected majority mutations for the four evolution lines. The black solid line indicates a mutation frequency of 50%. Mutation frequencies are unlikely to be informative once they fall below the Illumina sequencing error rate of ∼0.1%. The evolution lines are colored as in (*C*). (*D*) The mutation g9412a becomes a majority mutation in three populations in parallel except for MT-2_1. (*E*) Mutation frequencies of mutation, t9528g, increase almost simultaneously for all four evolution lines. (*F*) In all four evolution lines, the mutation g618a increases in frequency; in two lines, it becomes a majority mutation at the beginning of the experiment; and in one line toward the end of the experiment. For the frequency dynamics of other majority mutations, please see supplementary figure S2, Supplementary Material online.

We observed a total of 92 mutations across the four evolution lines (69 unique mutations) that became more frequent in the population than the wild-type nucleotide (fig. 2*A*). We call these mutations majority mutations. Although we expected the accumulation of majority mutations to decelerate when a population approaches an adaptive peak, we did not observe any deceleration during the 90 transfers we considered in this study.

To understand why mutations continue to accumulate at a high rate even toward the end of the experiment, we determined the time point of the maximum fold increase of the frequencies for each majority mutation (fig. 2*B*). These maximum fold increases should correspond to “fitness” because a large increase in the frequency of a novel mutation could be indicative of greater reproductive success of the viruses that carry this mutation. However, one has to keep in mind that the reproductive effect of a novel mutation is not the only factor affecting the frequency increase of that mutation. Random fluctuations (especially for low-frequency mutants), clonal interference, recombination, and linkage can also substantially affect mutation frequency differences. Nevertheless, our analysis shows that the “fitness gains” of majority mutations significantly decrease over time at similar rates. However, the fitness gains for majority mutations in MT-2 evolution lines initially are much larger than the fitness gains of majority mutations in MT-4 evolution lines (fitted regression lines in fig. 2*B*). The difference in the fitness gains suggests that the viruses growing on MT-4 evolution lines might be closer to an adaptive peak than the viruses growing on MT-2 evolution lines.

For all four evolution lines, we observe that fitness gains of novel majority mutations decrease over time. This could be due to diminishing fitness effects, or more clonal interference as the sequence diversity increases in the population. Diminishing fitness effects are expected for populations that are adapting to a new environment (Lenski et al. 1991; Wiser et al. 2013). Hence, the fitness effects we observe in our experiment of new mutations are decreasing as expected. Nevertheless, the number of new majority mutations observed at each time step does not decrease (fig. 2*A*). This pattern is similar to the linear accumulation of mutations observed in the long-term evolution experiment in *E. coli*, which the authors explained through an accumulation of a large number of small effect mutations (Barrick et al. 2009). However, for our experiment, there is an alternative explanation for the observed pattern. Due to the smaller population sizes in our experiment, neutral majority mutations are expected to accumulate toward the end of the experiment. These neutral majority mutations could maintain a constant accumulation rate of majority mutations.

The appearance of neutral majority mutations at the end of the experiment is supported by simulations. We performed simulations of neutrally evolving viruses using experimental data on population sizes at the start of every ten transfers (fig. 2*A* and supplementary fig. S1, Supplementary Material online). We simulate the evolution of individual HIV-1 genomes for 180 generations with a periodic bottleneck every two generations. Between bottlenecks, the simulated HIV-1 populations replicate exponentially (*R*_0_=44). Our results suggest that neutral majority mutations appear at the end of the experiment because it takes time for neutral mutations to accumulate in a population before they can become majority mutations (thin lines in fig. 2*A*).

We also analyzed the effect of APOBEC3G on the accumulation of majority mutations. APOBEC3G is only expressed in MT-2 T-cells and not in MT-4 T-cells (Borman et al. 1995). Interestingly, the proportion of G to A majority mutations at the end of the experiment is higher in HIV-1 growing in MT-4 T-cells (24 G to A mutations or 67%) than in HIV-1 growing in MT-2 T-cells (24 G to A mutations or 44%). Among these mutations, five were found in the APOBEC3G motif (GG to GA) in MT-2 and eight were found in MT-4 HIV-1 populations. Hence, it is quite clear that APOBEC3G expression does not drive the accumulation of majority mutations through an increased mutation rate. However, it is not clear whether there is an indirect effect of APOBEC3G expression (e.g., stronger selective pressure) that drives the faster accumulation of majority mutations in MT-2 virus populations.

### Large Extent of Parallel Evolution across Evolutionary Lines

**Fig. 3.**
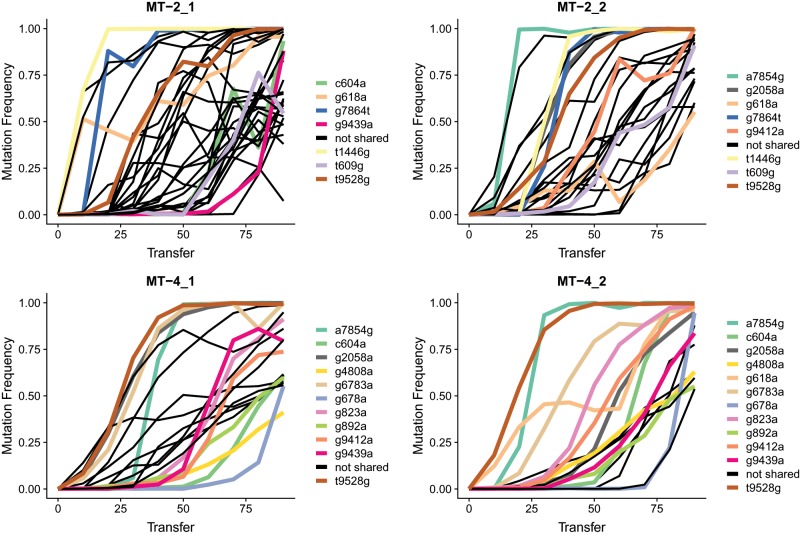
Frequency dynamics of majority mutations show decoupling of mutations. We observe decoupling when the mutation frequency dynamics of two mutations changes from being identical to becoming different. For example, in MT-2_1, there is a yellow and a black mutation that show the exact same frequency increase from transfer 0 to 10. At transfer 20, the mutation frequencies are different. Hence, at transfer 20, the two mutations decoupled probably through recombination. Colored thick lines in each of the four graphs indicate mutations that are acquired in more than one evolution line in parallel. The same mutations are drawn in the same colors. Black thin lines are majority mutations that are only found in a single line.

Consistent with the large observed fitness effects of the majority mutations, we see many of them arise in parallel in more than one evolution line (fig. 2*C*–*F* and colored lines in fig. 3). To test whether this observation is expected for neutrally evolving viruses with even mutation rates across the genome, we performed simulations (see Materials and Methods). For each evolution line, we distributed the observed majority mutations at transfer 90 across the genome (i.e., an observed A to G majority mutations will remain an A to G substitution albeit at another position in the genome). We then determined how many of the mutations occurred in more than one evolution line. We repeated the simulation 100 times and found that in only 28 of 100 simulations there was a single mutation that occurred in more than one evolution line. In the remaining 72 simulations, we found no mutations in more than one evolution line. Hence, we expect that almost all parallel mutations we observe in our experiment are adaptive or due to mutational hotspots (Cuevas et al. 2015; Sackman et al. 2017; Lind et al. 2019).

In the most extreme case, out of 17 majority mutations observed in one of the MT-4 evolution lines (MT-4_2) at transfer 90, 12 were shared with the other evolution lines. Of the remaining five private majority mutations from MT-4_2, two were also present at frequencies >10% in other evolution lines (g909a at 15% in MT-4_1 and g614a at 43% in MT-2_1, supplementary table S1, Supplementary Material online). In MT-4_1, 11 of 20 majority mutations also reached majority status in at least one other line. Of the remaining nine mutations, two occurred at frequencies >10%.

In viral populations grown on MT-2 T-cells, parallelism was not as high. Only 7 of 29 majority mutations in MT-2_1 occur in another evolution line, and only 8 of 26 majority mutations for MT-2_2. Additionally, five private majority mutations in MT-2_1 and three such mutations in MT-2_2 are present at frequencies >10% in other evolutionary lines. Notably, we observed one majority mutation (t9528g) that occurred across all four replicates and six mutations that occurred in three replicates in parallel (fig. 2*C*).

Interestingly, there was one synonymous mutation that also evolved in parallel (g4808a) of a total of 16 synonymous mutations. Out of 41 nonsynonymous mutations, we observed seven majority mutations in more than one line. Hence, the ratio of parallel mutations to private mutations is about three times as high in nonsynonymous mutations (17%) compared with synonymous mutations (6.2%). This difference, however, is not significant (Fisher’s exact test: *P* value = 0.42). The degree of parallelism is higher for majority mutations in untranslated regions of the genome: Of 26 mutations, 7 were shared (27%, but again not significantly different to both, parallel synonymous [*P* = 0.21] and nonsynonymous majority mutations [*P* = 0.53], Fisher’s exact test).

Apart from highlighting parallel mutations, figure 3 also shows that there are a few instances where recombination has decoupled the increase in frequency of a few mutations from each other. For example, the first mutation to attain a frequency >50% in MT-2_1 (t1446g, yellow) rises simultaneously with a private mutation (not parallel, g3078a, black). Both mutations reach a frequency of 66% at transfer 10. At transfer 20, the two mutations decouple in their increase: while t1446g has risen to 99%, g3078a has only increased to 75%. Similarly, g618a (orange) in MT-2_1 first increases in parallel with the private mutation t8700c (black) to reach 51% of the population at transfer 10. Subsequently, the mutations decouple. But, in this case, interestingly, the private mutation keeps increasing in frequency to 82% at transfer 20 while g618a decreases to 45%. There are other examples in figure 3 showing similar patterns. These patterns strongly suggest that recombination occurs frequently in our evolution experiment.

### Majority Mutations Appear in a Different Order, Suggesting Low Levels of Sign Epistasis between Parallel Majority Mutations

**Fig. 4.**
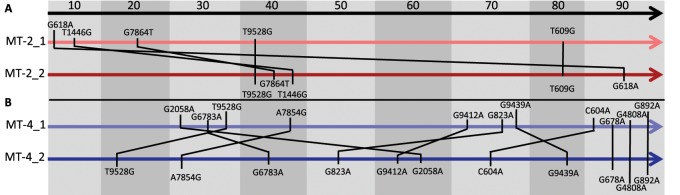
Order of majority mutations shared between evolution lines from transfer 10 to 90. (*A*) All majority mutations shared by MT-2_1 and MT-2_2. Black lines connect identical mutations. (*B*) Majority mutations shared by MT-4_1 and MT-4_2.

Although many majority mutations occur in parallel across evolution lines, their chronological order is different (fig. 4). For example, one of the mutations, that exceed the frequency of the wild-type nucleotide at transfer 10 in MT-2_1 (g618a), becomes a majority mutation only at transfer 90 in MT-2_2. Similar permutations of the chronological order are observed for the MT-4 evolution lines. A different order of mutations suggests that they are beneficial in different genetic backgrounds. Although we cannot exclude that the extent of the benefit is modulated by the genetic background, the benefit is apparently not turned into a deleterious effect. This means that there is no sign epistasis between the parallel majority mutations in our experiment.

### Implications of Parallelism for the Reconstruction of the Evolutionary History

**Fig. 5.**
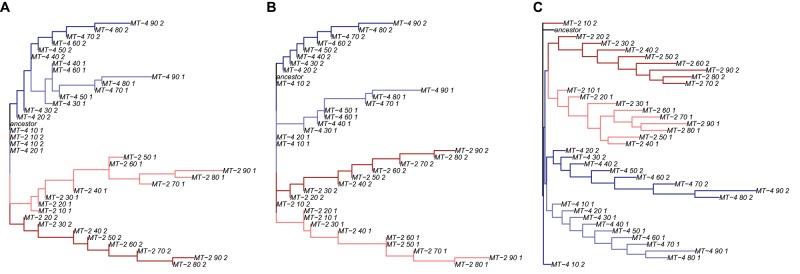
Phylogenetic trees. (*A*) Phylogeny inferred from the observed consensus sequences. (*B*) A phylogenetic tree, of which the topology reflects the correct evolutionary history. The tree was generated by randomly (for each evolution line independently) distributing the number of observed majority mutations across the HIV-1 genome. The phylogenies in (*A*) and (*B*) were inferred with PhyML (Guindon et al. 2010). (*C*) Phylogeny is based on the differences between all mutation frequencies that were >1% in our data set. For all pairwise differences, we constructed a distance matrix. We used the distance matrix to infer the phylogeny via Neighbor joining as implemented in the R package ape (Paradis et al. 2004). The trees were colored with phytools (Revell 2012). The colors correspond to the four evolution lines and are consistent between (*A*–*C*).

The high level of parallelism we find in our experiment poses a challenge for the reconstruction of the evolutionary history of our viral populations. When inferring the evolutionary history for the consensus sequences at every tenth transfer, the history cannot be reconstructed correctly for MT-4 evolution lines, where we observe the highest level of parallel evolution. Instead, the sequences cluster by the environment they evolved in (fig. 5*A*). The mixing of MT-4_1 and MT-4_2 evolution lines in this tree would be wrongly interpreted as a signature of viral migration in a phylogeographic analysis.

The correct evolutionary history (fig. 5*B*) can be inferred from simulated sequence data, in which the observed majority mutations are randomly redistributed across the HIV-1 genome (see Construction of Neutral Phylogenies in Materials and Methods). When statistically comparing the correct phylogeny with the one reconstructed from the sequences, the hypothesis that our viral populations evolved neutrally can be rejected with high confidence (Likelihood ratio = 3.5 × 10^−69^, see Construction of Neutral Phylogenies in Materials and Methods).

After failing to reconstruct the correct phylogeny for our evolution lines from majority mutations, we decided to include minority mutations in our analysis. Unfortunately, commonly used phylogenetic methods cannot take the frequency of minority mutations into account when reconstructing phylogenies. Hence, we developed our own method. This method calculates the genetic distance between two populations by summing up the absolute differences in frequency of all wild-type nucleotides and all three possible point mutations. We calculated the distances between all sample pairs and with this distance matrix inferred a phylogeny with Neighbor Joining (Saitou and Nei 1987). The inferred tree recapitulates the evolutionary history better than the majority mutation tree (fig. 5*C*). Both MT-4 lines are now clustered separately on the tree. This means that the two evolution lines genetically diverge early at the level of minority mutations but not at the level of majority mutations.

### d*N*/d*S* Method Identifies Parallel Majority Mutations

To identify positively selected substitutions, one commonly applies d*N*/d*S* methods. These methods assume synonymous substitutions as selectively neutral (Kimura 1977). This assumption allows the estimation of selection by comparing the number of expected synonymous substitutions with the expected number of nonsynonymous substitutions (d*N*/d*S*) (Yang and Bielawski 2000). An excess of nonsynonymous substitutions indicates positive selection, an excess of synonymous substitutions purifying selection. More sophisticated methods, such as CodeML (Yang 2007) also take the phylogenetic relationship between sequences into account to identify positively selected codons.

We used CodeML to infer positively selected codons. In our case, we can provide CodeML with the consensus sequences of the four evolution lines at all time points and the three different trees from figure 5. Based on our sequence analyses, we expect that CodeML, a package from the PAML suite of programs, will identify the seven majority mutations that occurred in parallel across evolution lines as positively selected, if we provide CodeML with the tree that reflects the true evolutionary history (fig. 5*B*). As expected, CodeML identifies all seven parallel nonsynonymous majority mutations as likely positively selected (fig. 5*B* and supplementary table S2, Supplementary Material online). However, it also infers two more mutations that appear, disappear, and reappear as majority mutations due to changes in mutation frequency. These fluctuations in mutation frequency are incorrectly interpreted as independent substitution events because CodeML is designed to analyze fully speciated organisms and not evolving populations. These two mutations are also inferred as positively selected when providing the other two phylogenies (fig. 5*A* and *C*). CodeML performs worse when supplying the inferred phylogeny and the minority mutation phylogeny. Hence, our analysis shows that CodeML also identifies parallelly evolving nonsynonymous sites as positively selected.

### The Distribution of Minority Mutations as an Indicator for Selection Strength

**Fig. 6.**
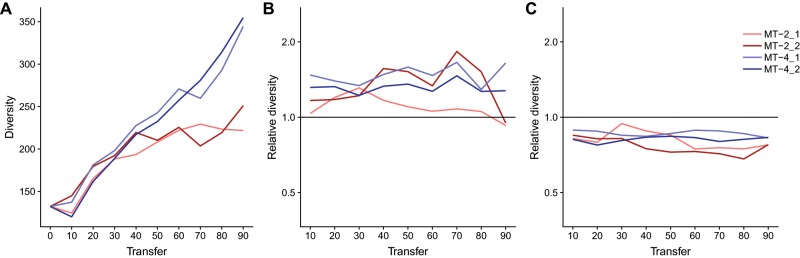
Nucleotide diversity observed across time for all four evolutionary lines. (*A*) We applied the Shannon Entropy as diversity measure, which we calculated for each nucleotide site with coverage of more than 1,000 sequence reads and then summed it up across the HIV-1 genome. (*B*) Relative diversity measures for the 3′ noncoding region, the most diverse region in the HIV-1 genome in our experiment. To calculate relative diversity, we divide the average diversity across one gene by the average diversity across the entire genome. Hence, values >1 indicate higher than average diversity and <1 a lower than average diversity. (*C*) Like (*B*), just for the least diverse gene *gag* in our experiment.

In our experiment, we do not have to solely rely on majority mutations to gauge selection strength, we can also include all mutation frequencies in the population by using common diversity indices. We find that over time the nucleotide diversity (measured as the Shannon entropy summed over all nucleotide sites) in all four HIV-1 populations increases (fig. 6*A*). This mirrors the pattern that has previously been observed for HIV-1 in vivo (Shankarappa et al. 1999). We observe that diversity increases faster in MT-4 T-cells than in MT-2 T-cells late in the experiment.

We also measure nucleotide diversity for each gene separately (fig. 6*B* and *C* and supplementary figs. S3 and S4, Supplementary Material online). Interestingly, genes show similar levels of diversity in the different evolution lines. Across the experiment diversity is greatest in *nef* and the 5′ and 3′ noncoding regions (fig. 6*B* and supplementary fig. S4, Supplementary Material online). *Nef* is particularly diverse in HIV-1 populations growing on MT-4 T-cells, whereas the 5′ noncoding region is the most diverse region in MT-2 virus populations. The genes with the lowest diversity during the experiment are *gag* and *tat* (fig. 6*C*).

**Fig. 7.**
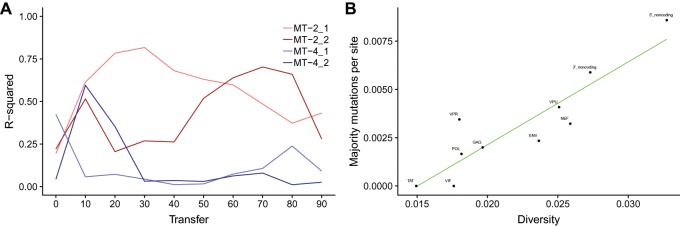
Mutation diversity is predictive of observed parallel mutations at transfer 90. (*A*) Correlation between mutation diversity from all measured time points and parallel majority mutations observed at transfer 90 for all genes and noncoding regions in the HIV-1 genome of all four lines. *R*^2^ values are a measure of how close the data are to the fitted regression line. High *R*^2^ values indicate good correlations, low *R*^2^ values indicate bad correlations. (*B*) The linear model fitted for the diversity observed for all genes and noncoding regions at transfer 30 and the number of parallel majority mutations in the same regions for MT-2_1 at transfer 90.

We hypothesize that genes with high nucleotide diversity should accumulate large numbers of parallel majority mutations and genes with low nucleotide diversity should accumulate low numbers of parallel majority mutations. When we correlate the nucleotide diversity at transfer 90 with the number of parallel majority mutations at transfer 90, we find relatively weak correlations ranging from *R*^2^ values of 0.03 for MT-4_2 to 0.43 in MT-2_1, with only MT-2_1 showing a significant *P* value of 0.04 (fig. 7*A*). However, when we correlate nucleotide diversity of all the earlier time points with the number of majority mutations that accumulate at transfer 90, we find much higher correlations (fig. 7*A*). For MT-2_1, we observe the best correlation at transfer 30 (*P* value = 0.0003, *R*^2^=0.79, fig. 7*B*). Interestingly, for each evolution line the time point is different at which the correlation between nucleotide diversity and number of parallel majority mutations at transfer 90 is highest. The *R*^2^ is highest at transfer 70, 0 and 10 for MT-2_2 (*R*^2^=0.70, *P* value = 0.002), MT-4_1 (*R*^2^=0.42, *P* value = 0.04), and MT-4_2 (*R*^2^=0.60, *P* value = 0.009), respectively. Hence, nucleotide diversity at earlier time points is predictive of the number of parallel majority mutations observed at the end of our experiment, at a gene by gene basis.

## Discussion

In this study, we investigate the long-term evolutionary dynamics of HIV-1 NL4-3 in four independent evolutionary lines. We find that the four viral populations rapidly and continuously diverge from the ancestor over the course of almost an entire year. The mutation dynamics suggest that at least the first mutations that accumulate in our experiment confer large fitness benefits to the virus. Hence, our four evolution lines respond to large selective pressures despite the absence of antiviral drugs or the immune system. The selective pressures the viral populations respond to could be due to fluctuating selection (alternating transfers last 3 or 4 days), adaptation to different T-cells, or adaptation to growing in flask cell cultures.

Detailed analyses of the mutation dynamics suggest that mutational fitness effects are decreasing over the course of our evolution experiment, similar to diminishing fitness effects found in the Lenski experiment (Wiser et al. 2013). We find that the fitness effects decrease at similar rates for HIV-1 populations growing on MT-2 and MT-4 T-cells. However, viruses growing on MT-2 cells show greater fitness effects early on in the experiment, indicating that HIV-1 NL4-3 is closer to a local optimum in the MT-4 fitness landscape than in the MT-2 fitness landscape. In line with this hypothesis, mutations sweep through the MT-4 viral populations at a lower rate. Being closer to a local optimum in a fitness landscape also means that there are fewer beneficial mutations available (Silander et al. 2007). If there are fewer beneficial mutations available then it is more likely that, in two independent populations, the same mutations are selected to become majority mutations. This is exactly what we observe: a higher proportion of sites that evolve in parallel between the two MT-4 viral populations. Also relevant in this context are the less stringent bottlenecks (larger *N*_e_) that MT-4 viral populations undergo (supplementary fig. S1, Supplementary Material online). This increases the balance between drift and selection in favor of selection in the MT-4 evolution lines and thereby increases the proportion of parallel mutations.

Since our analysis suggests that parallel mutations are likely positively selected, we mined the existing literature to determine the biological relevance of these parallel mutations. The most common parallel nonsynonymous mutation is a7864g (also known as D547G in ENV, or D36G in gp41). This mutation is a reversion to the database consensus of glycine at position 547 in the ENV protein. Aspartate at position 36 has been shown to confer resistance to enfuvirtide, a fusion inhibitor (Marconi et al. 2008). There is one more nonsynonymous mutation (g2058a or M423I in GAG, M46I in p7) that occurs in three evolution lines in parallel. This mutation is also extremely common in other evolution experiments that were performed in the presence of protease inhibitors (Koh et al. 2009). Unfortunately, these in vitro evolution experiments rarely comprise a drug free control. Hence, it cannot be determined if the mutation has been selected by the drug, or if it constitutes an adaptation to the host cell type. Our experiment suggests that it is a general adaptation to the host cell type or laboratory environment. We found another GAG mutation (t1446g or H219Q in GAG or H87Q in p24) that only occurs in evolution lines growing on MT-2 T-cells. This mutation has also been reported previously and found to increase replicative capacity in cyclophilin-A rich cells (such as MT-2) (Gatanaga et al. 2006). There are four more nonsynonymous mutations that we found in more than one evolution line (E12K and V35I in GAG, S190N and Q550H in ENV). Except for one (S190N in ENV), all of them were reported in previous evolution experiments: one emerged in the absence of drug pressure (V35I in GAG; Lorenzo-Redondo et al. 2011), the other two were found to evolve in the presence of antiviral drugs (Nameki et al. 2005; Aoki et al. 2009). The emergence of adaptive mutations in our long-term, drug-free HIV evolution experiment emphasizes that drug free control lines in in vitro experiments are required to ascertain that a mutation confers drug resistance rather than an adaptive advantage at growing in the host cell line.

Most of our parallel mutations (7 out of 15) occurred in the untranslated part of the HIV-1 genome, which only makes up 5.5% of the HIV-1 genome. Unfortunately, there is little information on the function of these mutations. However, there is evidence that the host cell environment significantly affects replication rates for different LTR (long terminal repeat) sequences (van Opijnen et al. 2004). Interestingly, five of the seven parallel majority mutations occur in three of the four evolution lines and only two are host cell specific. Hence, our data suggest that HIV-1 NL4-3 adapts to in vitro growth conditions rather than to the specific host cell environment. Alternatively, one could argue that the virus adapts most to environmental features that are common between MT-2 and MT-4 cells.

The large extent of parallel evolution has also a strong effect on our ability to infer the evolutionary history from the sequence data at the end of the experiment. If parallel evolution also plays a significant role in vivo, then we expect HIV-1 phylogenies to also reflect host similarities, particularly when the viruses have evolved for long periods of time within these hosts. Indeed, parallel evolutionary changes have been observed in vivo in genetically identical twins infected with the same viral strain as well as in HIV-1 populations during early infection and to a smaller extent also in other studies (Draenert et al. 2006; Wood et al. 2009; Bertels et al. 2018).

Nevertheless, reconstructed transmission trees have been found to be consistent with a small number of epidemiologically confirmed transmission chains (Leitner et al. 1996). Although the reliability of such reconstructions is still being investigated (Leitner et al. 1996; Romero-Severson et al. 2014), it is conceivable that transmission histories can be reconstructed accurately if each host exerts a specific selection pressure, thus leaving a signature in the viral genome unique to the infected host (Carlson et al. 2016). Such an effect is also apparent in our experiment: The extent of parallel evolution between populations growing on different T-cell lines is relatively low (fig. 2*C*). But one has to keep in mind that even a small number of parallel mutations could cause distortions in the branch lengths and the topology of a phylogenetic tree (Bertels et al. 2014).

The levels of parallel evolution we found may be particularly relevant for phyloanatomical approaches (Salemi and Rife 2016; Bons and Regoes 2018) that have received a lot of attention recently (Lorenzo-Redondo et al. 2016). Phyloanatomy entails the application of phylogeographic methods to viral genetic data sampled from different anatomical compartments within infected individuals. The aim is to reconstruct replication rates within each anatomical compartment, and the rates of migration between these compartments. The basis for estimating the migration rates is the presence of the same mutation in more than one compartment, which, according to a neutral model, is most likely due to a virus migrating from one compartment to another. With high rates of parallel evolution, however, such an inference cannot be drawn. Indeed, the phylogenetic tree constructed with the majority sequences obtained from our four independently evolving viral populations wrongly suggests migration events between the populations. The implications of our experiments for phyloanatomical analyses are more immediate than for the reconstruction of epidemiological transmission history of the virus because selection by the immune system in different hosts might lead to divergent viral sequences, making the inference of the transmission history from viral sequences possible. Within a single host in contrast, immune selection is likely to be more homogeneous across different compartments, and will not generate divergent lineages. It may generate even more parallel evolution (Vanderford et al. 2011), which will further confound a phyloanatomical analysis.

We tried to alleviate this issue by building a tree from a distance matrix calculated by subtracting mutation frequencies between different sequencing samples. The resulting phylogeny separates the two MT-4 lines better than the tree that was only built from majority mutations. Nevertheless, there are still numerous inaccuracies of the tree topology, which will lead to issues in downstream analyses as shown with our CodeML analysis. However, since most studies nowadays take advantage of deep sequencing this method could supplement classical phylogenetic analyses when the evolutionary history of sampled populations is not known.

The extent of parallel evolution in our experiment is likely to be determined by the number of high-fitness effect mutations available during adaptation (Orr 2005; Bons et al. 2018). Interestingly, the majority mutations that evolve in more than one line do not necessarily appear in the same chronological order in different evolutionary lines. This suggests that these mutations not only have a high fitness effect but also that the effect is independent of the presence of other majority mutations that have been acquired previously. Technically speaking, there appears to be only low levels of sign epistasis between these mutations. If the number of mutations with high fitness effect were large then picking the same mutation twice is unlikely. Similarly, if their effect strongly depends on the genetic background, populations would diverge on different evolutionary trajectories. Hence, we can explain high parallelism and different mutation orders with a relatively simple argument: for each population, mutations are independently drawn from the same small pool of large effect mutations.

Apart from low levels of sign epistasis and large fitness effects, parallel evolution could also be explained by differences in mutation rates across the genome, which could for example happen when APOBEC3G affects viral evolution (Cuevas et al. 2015; Bertels et al. 2018). High mutation rates at certain positions in the genome could lead to increased levels of parallel evolution at these locations in different evolution lines (Bauer and Gokhale 2015). Similar observations have been made in other model organisms (Sackman et al. 2017; Lind et al. 2019). However, it is very challenging to disentangle the contributions of mutation rate differences and selection to the levels of parallel evolution in our experiment.

We also show that the level of nucleotide diversity in each gene early in the experiment is predictive of the number of majority mutations that accumulate in these genes by the end of the experiment (fig. 7). Interestingly, the point at which the correlation between diversity and number of majority mutations was highest was different between the replicate lines. The cause of these differences is unclear, but one contributing factor could be the changing bottleneck sizes in our experiment. We postulate at least two causes for the correlation between diversity and the accumulation of majority mutations. Either fitness effects or mutation rates (or both) may be unevenly distributed across the genome. In future studies, we hope to improve our understanding of this phenomenon.

There are only few long-term evolution experiments with viruses (Holland et al. 1979; Kearney et al. 1999; Wichman et al. 2005). An interesting experiment with viruses was conducted with phage φX174 growing on *E. coli* (Wichman et al. 2005). The phage population grew in a chemostat for 6 months and ∼13,000 generations. At the end of the experiment, 137 substitutions were identified, which is ∼4- to 8-fold higher than in our experiment. In our experiment, we observe between 17 (MT-4_2) and 29 (MT-2_1) majority mutations after ∼1 year, but considering that HIV-1 undergoes only 180 generations this difference is not surprising. Despite the 70-fold difference in the number of generations, we only observe an ∼7-fold lower number of majority mutations in HIV-1. There are at least two factors affecting this difference, first the 5-fold higher mutation rate of HIV-1 (∼2 × 10^−5^; Mansky 1996) compared with φX174 (∼1 × 10^−6^; Cuevas et al. 2009), and the second probably more important factor is the much smaller population size in HIV-1 (bottleneck sizes between 40 and 1,600) compared with φX174 (∼10^9^). Smaller population sizes lead to significantly shorter fixation times. Neutral mutations sweep through the population in about 2 *N*_e_ generations (Kimura 1983), which is a tremendously long period of time in the φX174 experiment (∼76,000 years) but likely to occur in our HIV-1 experiment (>160 days).

In conclusion, our experiment suggests that the high level of parallel evolution we observe is the result of a limited number of large effect mutations with low levels of sign epistasis between them. The high level of parallel evolution together with the observation that genomic regions of high nucleotide diversity early in the experiment accumulate more majority mutations late in the experiment indicates that HIV-1 evolution may be predictable.

## Materials and Methods

### Passaging of HIV-1

The human T-cell leukemia cell lines MT-2 and MT-4 (Harada et al. 1985) were obtained through the AIDS Research and Reference Reagent Program, Division of AIDS, NIAID, NIH from Dr. Douglas Richman. Cells were maintained in RPMI 1640 medium containing 10% fetal calf serum, 100 U/ml penicillin, and 100 µg/ml streptomycin. The HIV-1 full-length plasmid pNL4–3 was obtained through the AIDS Research and Reference Reagent Program, Division of AIDS, NIAID, NIH from Dr. Malcolm Martin (Adachi et al. 1986). The virus stock HIV-1 NL4-3 was generated and characterized as previously described (Di Giallonardo et al. 2014). The cultures were grown in separate cell culture flasks. At day 0, 4×10^5^ cells per replicate and per cell line were infected with HIV-1 NL4-3 at an MOI (multiplicity of infection measured on peripheral blood mononuclear cells) of 0.01 resulting in four independent T-cell cultures (fig. 1). Virus passaging was performed twice a week (odd transfers after 3 days, even transfers after 4 days) as follows: Infected cell cultures were resuspended. About 4×10^5^ uninfected cells were inoculated with 2 µl cell suspension. Every tenth transfer, cell-free supernatant was stored at −80 °C. In the first 25 transfers, higher volumes (30–3 µl) were transferred based on the extent of cytopathic effects microscopically observed in the cell cultures.

### Ancestral Sequence

The HIV-1 NL4-3 ancestor has been sequenced and assembled previously (Di Giallonardo et al. 2014). Differences to the HIV-1 NL4-3 reference strain (accession AF324493) are listed in Supplementary Table 3 of the same article (Di Giallonardo et al. 2014).

### Sequencing of Near Full-Length HIV-1 Genomes

Near full-length genomes of the virus stock HIV-1 NL4-3 (ancestor) and transfers 10, 20, 30, 40, 50, 60, 70, 80, and 90 were sequenced using the Illumina MiSeq next-generation sequencing platform as previously described (Di Giallonardo et al. 2014). Briefly, HIV-1 RNA was isolated from 150 µl virus stock HIV-1 NL4-3 or cell-free supernatant; four samples per transfer. Five overlapping amplicons were generated by RT-PCR covering almost the full genome of HIV-1 per sample. In total, 185 amplicons were obtained (4 samples/transfer×9 transfers×5 amplicons/transfer + 5 amplicons of the ancestral virus) after one round of PCR. The five amplicons per sample were pooled and libraries were prepared with the Nextera XT DNA Sample Preparation Kit (Illumina, San Diego) according to the manufacturer’s description. Next-generation sequencing was performed using a MiSeq Benchtop Sequencer generating paired-end reads of 2 × 250 bp length (v2 kit). To minimize the risk of cross-contamination, samples from each replicate line were processed separately. Samples were pooled after barcoding and the following samples were sequenced on one chip: Chip 1) virus transfers 10–60; chip 2) virus transfers 70–90; and chip 3) the virus stock among other samples.

### Determining 50% Tissue Culture Infectious Dose and MOIs for Every Tenth Transfer

To determine the number of infectious viruses, we plated increasing dilutions of the cell free supernatant on 1,000 cells each. For each of the nine time points and four evolution lines, we performed four replicates. After 7 days, we determined the number of cell cultures that were successfully infected by checking for cytopathic effects under the microscope. This allows us to estimate the 50% tissue culture infectious dose (TCID50) (Reed and Muench 1938). Using the TCID50 and the number of cells (400,000) and transfer volumes (2–10 µl) in our experiment, we can calculate the average number of viruses (MOI) that successfully infect a single T-cell in the first generation of every tenth transfer in our experiment.

### Mutation Identification from Illumina Sequencing Data

Illumina sequences were aligned to the ancestral sequence via Bowtie 2 (Langmead and Salzberg 2012). All aligned nucleotides with a quality score of >35 (i.e., error probability of <0.00032) were considered. The first 80 bp and last 30 bp of the HIV-1 genome were excluded from all analyses due to low and spurious coverage. The remaining genome was covered in all data sets by at least 1,000 nucleotides. Mean coverage for any sequence sample was at least 2,912 nucleotides (supplementary fig. S5, Supplementary Material online).

Consensus sequences were determined by selecting the most common base at each nucleotide site.

### Construction of Neutral Phylogenies

For each transfer, we simulated sequence evolution under a neutral evolutionary model. We randomly distributed the same number of majority mutations that occurred from one transfer to the next for each evolution line across the HIV-1 genome. When introducing mutations we maintained the GC content of the ancestral HIV genome. Hence, at the end of the simulation, the sequences contained the exact same number of majority mutations as the consensus sequence sampled in our experiment. From these sequences, we built the phylogenetic tree shown in figure 5*B*.

To determine the robustness of the neutral phylogenetic tree, we repeated the sequence simulation 100 times. The log-likelihood of the correct trees (inferred from randomly distributed mutations) for the observed consensus sequence alignment is −13,375.7 with a SD of 1.1. We call trees correct that resemble the true setup of the experiment, that is, trees expected for neutrally and independently evolving sequences with even mutation rates across the genome. In contrast, the most likely tree for our consensus sequences has a log-likelihood of −13,218.1, which is ∼3.5×10^69^ times greater than the correct trees. The most likely tree does not correspond to the real evolutionary history of the sequences because it clusters MT-4 strains together, although in the experiment they kept completely separate from each other. In figure 5*B*, we only showed a single example for illustrative purposes.

### Phylogenetic Analyses

We inferred all phylogenetic trees with PhyML (Guindon et al. 2010) under a general time reversible nucleotide substitution model and gamma distributed rate variations.

### Phylogeny Based on Minority Mutations

To calculate a high-quality phylogeny, we first eliminated all mutations that occurred at frequencies of <1% in the population and included only sites with >1,000-fold sequence coverage. For the remaining mutations, we calculated the absolute distances $D_{\mathrm{ij}}$between all populations at all time points:

$$D_{\mathrm{ij}}=\sum_{o=1}^{l}\sum_{p\mathrm{in}[\mathrm{ATCG}]}{|f}_{\mathrm{iop}}-f_{\mathrm{jop}}|$$

*D_ij_ The absolute distance between population i and population j. L Length of the HIV genome. P Nucleotide at position o. F Frequency of the nucleotide p at position o in population i or j.*

From the distance matrix $D_{\mathrm{ij}},$ we calculated a Neighbor-joining tree in R with the ape package (Paradis et al. 2004).

### Likelihood Ratio between the Correct and the Inferred Phylogeny

To assess the likelihood that the sequences of our experiment evolved under a neutral model of evolution, we determined the likelihood of the simulated correct tree for the observed consensus sequence and compared it to the likelihood of the tree inferred from the consensus sequence alignment.

### Determining Maximum Fitness

For each mutation that became a majority mutation at transfer 90, we determined the maximum fitness by calculating the maximum increase in mutation frequency between two consecutive 10th transfers. We also distinguished between viruses growing on MT-2 and MT-4 T-cells. We then fitted a linear model to the three data sets and got the following results. For the entire data set, the decline slope (log_10_(fitness) declines by 0.006779 per transfer) was insignificantly different from zero (*P* value = 0.4). For MT-2, the decline slope was even smaller (log_10_(fitness) declines by −0.001345 per transfer) and also not significantly different from zero (*P* value= 0.9). For MT-4, however, the decline slope was larger (log_10_(fitness) declines by 0.02762 per transfer) and significantly different from zero (*P* value = 0.02762).

### d*N*/d*S* Calculations

d*N*/d*S* calculations were done using the Ka_Ks calculator (Zhang et al. 2006). We applied the calculator with standard settings (model averaging) to the open reading frames of the ancestor aligned to consensus sequences at transfer 90.

### Calculating Diversity

We calculated diversity by measuring the Shannon entropy for each position in the HIV-1 genome that has a coverage of >1,000 bp. Then the diversity at position j at transfer t is:

$$D_{j}\left(t\right)=\sum_{i\mathrm{in}\{A,T,G,C\}}f_{\mathrm{ji}}\left(t\right)\log\;\left(f_{\mathrm{ji}}\left(t\right)\right)$$

*f_ji_ is the relative frequency of base I at position j at transfer t*

The per site diversity $\overset{-}{D}$ for gene x of length l is then:

$$\bar{D_{x}\left(t\right)}=\frac{\sum_{i=1}^{l}D_{i}\left(t\right)}{l}$$

and for the whole genome with length gl:

$$\bar{D_{w}\left(t\right)}=\frac{\sum_{i=1}^{\mathrm{gl}}D_{i}\left(t\right)}{\mathrm{gl}}$$

We defined the accumulated diversity $\bar{CD_{x}\left(t\right)}$ as:

$$\bar{CD_{x}\left(t\right)}=\sum_{i=0}^{t}\bar{D_{x}\left(i\right)}$$

The relative diversity for gene x, ${\mathrm{Drel}}_{x}$, is then:

$${\mathrm{Drel}}_{x}\left(t\right)=\frac{\bar{D_{x}\left(t\right)}}{\bar{D_{w}\left(t\right)}}$$

*Calculating correlation between diversity and per site majority mutations*

We defined the per site majority mutation numbers as:

$$\bar{{\mathrm{MM}}_{x}\left(t\right)}=\frac{{\mathrm{MM}}_{x}\left(t\right)}{\mathrm{length}\left(x\right)}$$

*MM_x_* (*t*) is the number of majority mutations found in gene *x* at transfer *t*

For figure 7*A*, we combined all $\bar{{\mathrm{MM}}_{x}\left(90\right)}$ from the four different lines and correlated it with all $\bar{D_{x}(t)}$ (where t runs from 0 to 90) individually, by inferring a linear model in R. We also correlated $\bar{{\mathrm{MM}}_{x}\left(90\right)}$ with the accumulated diversity $\bar{CD_{x}\left(t\right)}$, which slightly improved the fit.

For figure 7*B* and *C*, we inferred a linear model for all $\bar{D_{x}(t)}$ and $\bar{{\mathrm{MM}}_{x}(90)}$ from each line separately as well as between $\bar{CD_{x}\left(t\right)}$ and $\bar{{\mathrm{MM}}_{x}(90)}$.

### Simulation of Neutral Mutations

To predict how many neutral mutations would accumulate in our experiments, we simulated the evolution of individual HIV-1 genomes of length 9,130 for 180 generations (fig. 2*A*). We started our simulation with 400 individuals. Each individual genome is assumed to produce 44 offspring genomes per generation (*R*_0_=44). The viral population is allowed to replicate exponentially for two generations. Consistent with the experimental setup, we simulate a bottleneck every second viral generation, by randomly selecting a small number of individuals from the previous population. The size of the bottleneck, that is, the number of viruses transferred at every passage, was empirically determined. To this end, we determined bottleneck sizes every 20 generations in supplementary figure S1, Supplementary Material online. Because we do not have more detailed information on the number of transferred viruses we maintain the number for 20 viral generations which corresponds to ten transfers. Every newly produced viral genome can acquire a mutation with a probability of 2.16 × 10^−5^ (Lee et al. 2009).

### Determining Extent of Parallel Evolution in Neutrally Evolving HIV-1 Evolution Lines

We determined the level of parallel evolution that we would observe for neutrally evolving sequences. For this purpose, we randomly distribute the same number of mutations that we observed in the four evolution lines across the HIV-1 genome. When distributing substitutions we first randomly chose a position in the genome. The mutation introduced at this position was then determined from transition probabilities inferred from the mutations observed in full length *env* sequence data during early infection (Keele et al. 2008). Hence, we distributed 29 and 26 mutations for the MT-2 evolution lines and 20 and 17 mutations for the MT-4 evolution lines randomly across 9,130 unique HIV-1 NL4-3 nucleotide sites. Once the mutations were distributed we determined the number of mutations that occurred in more than one evolution line in parallel. We then repeated the entire simulation 100 times.

### Running CodeML

To identify positively selected sites in the translated part of the HIV-1 genome, we supplied CodeML with an alignment of the translated regions of all evolution lines and sequenced time points in the experiment where majority mutations are substituted for the wild-type allele. We ran CodeML three times each time supplying a different phylogeny, that is, the inferred phylogeny (fig. 5*A*), the true phylogeny (fig. 5*B*), and the phylogeny inferred from the comparison of minority as well as majority mutations (fig. 5*C*). We used the same settings as the HIVNSsites Sweden example. Briefly, the parameter values that were set to a nonzero value are: the CodonFreq model is set to F3X4, kappa is 0.3, omega is 1.3, ncatG is 10, cleandata is 1, and Small_diff is 0.45e-6.

## Supplementary Material

Supplementary data are available at *Molecular Biology and Evolution* online.

## Supplementary Material

msz155_Supplementary_Data
