# Supplementary material for: Parallel Evolution of HIV-1 in a Long-Term Experiment
Source: Mol Biol Evol. 2019 Jul 4;36(11):2400–14. doi: 10.1093/molbev/msz155 (PMC6805227; doi:10.1093/molbev/msz155)
Supplement: msz155_Supplementary_Data [file msz155_supplementary_data.zip › Figure_S2.pdf]

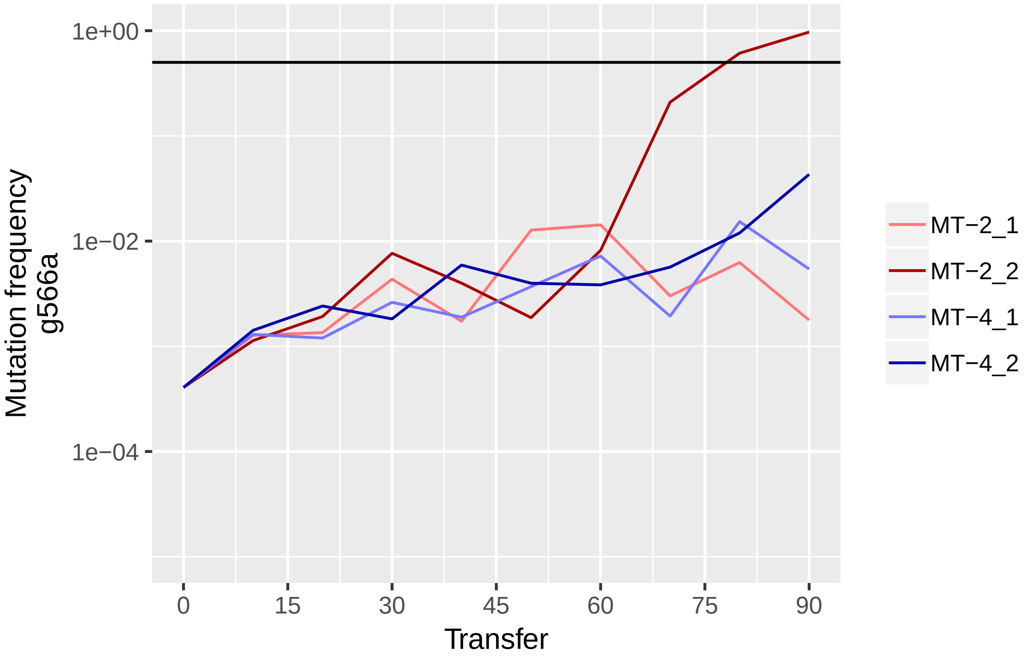

1e+00
1e−02
1e−04
Mutation frequency
g566a
0
15
30
45
60
75
90
Transfer
MT−2_1
MT−2_2
MT−4_1
MT−4_2

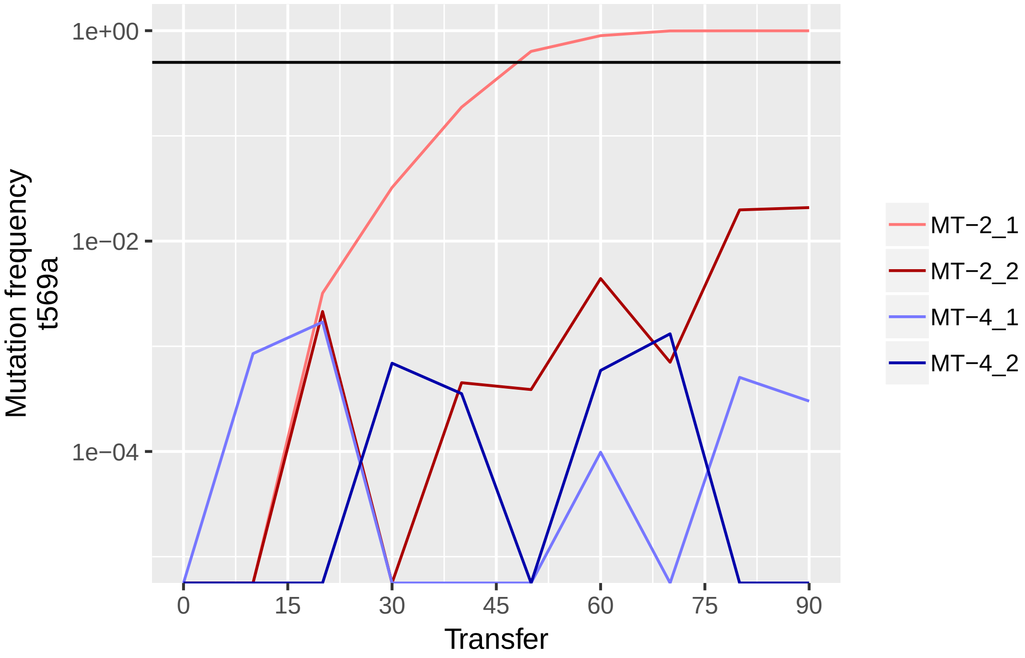

1e+00
1e−02
1e−04
Mutation frequency
t569a
0
15
30
45
60
75
90
Transfer
MT−2_1
MT−2_2
MT−4_1
MT−4_2

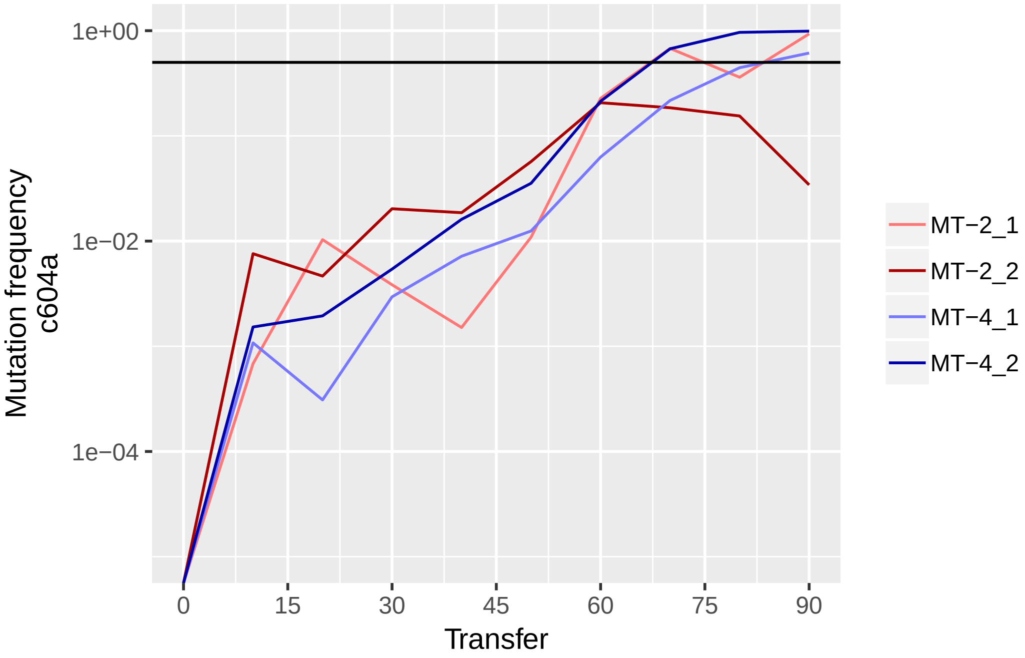

1e+00
1e−02
1e−04
Mutation frequency
c604a
0
15
30
45
60
75
90
Transfer
MT−2_1
MT−2_2
MT−4_1
MT−4_2

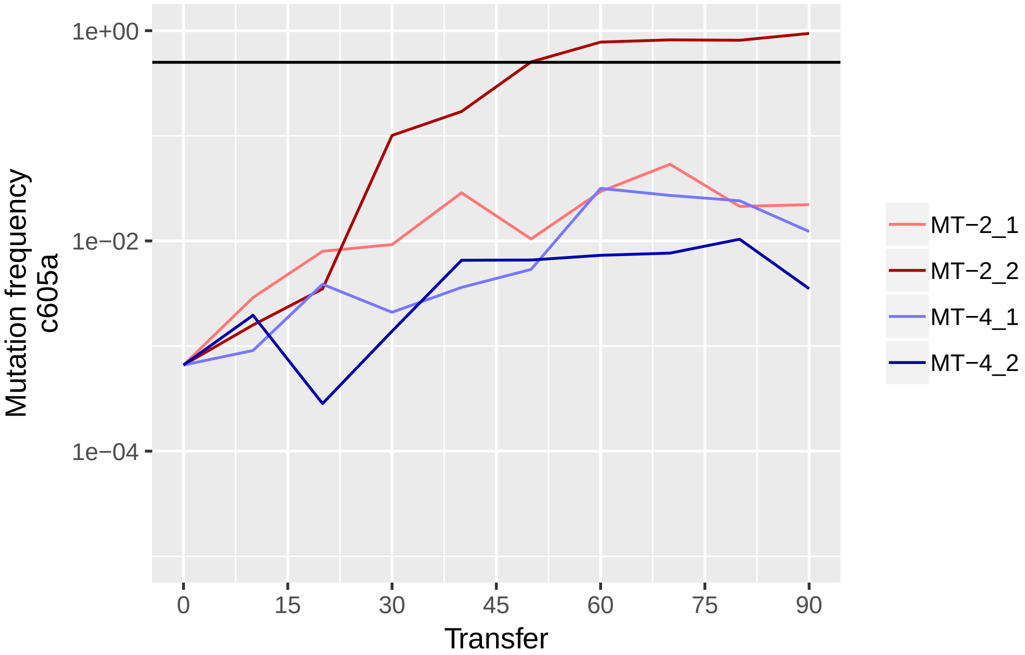

1e+00
1e−02
1e−04
Mutation frequency
c605a
0
15
30
45
60
75
90
Transfer
MT−2_1
MT−2_2
MT−4_1
MT−4_2

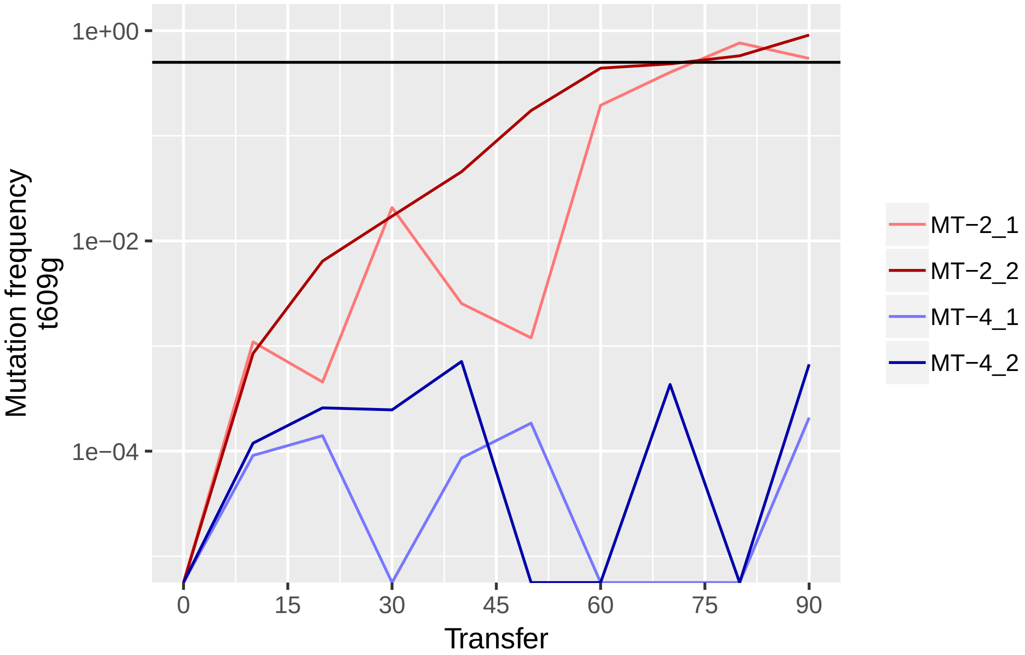

1e+00
1e−02
1e−04
Mutation frequency
t609g
0
15
30
45
60
75
90
Transfer
MT−2_1
MT−2_2
MT−4_1
MT−4_2

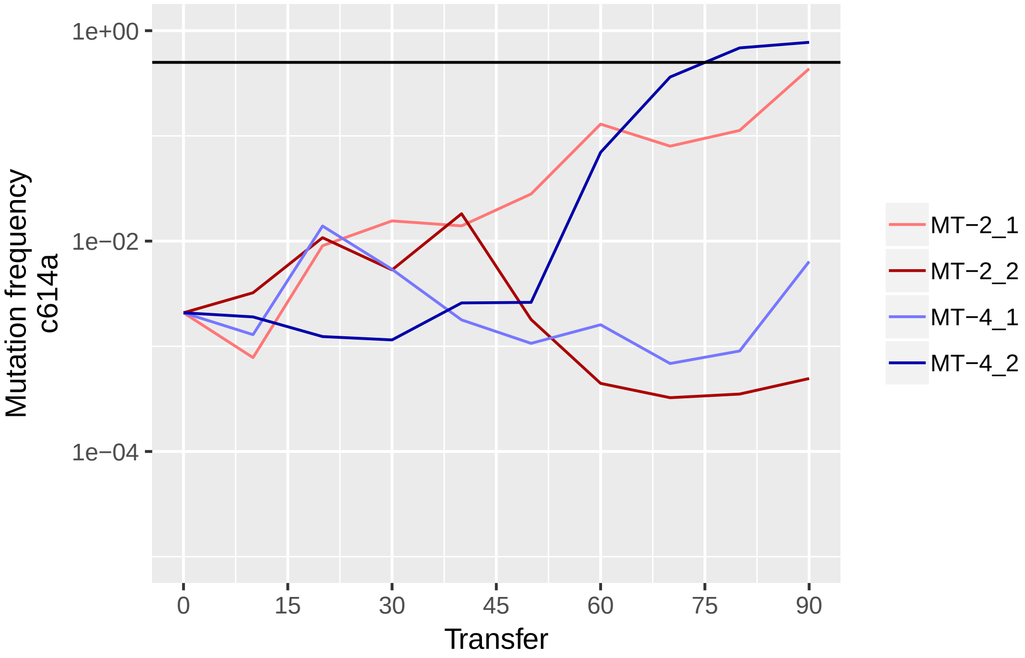

Mutation frequency
c614a
1e+00
1e−02
1e−04
0
15
30
45
60
75
90
Transfer
MT−2_1
MT−2_2
MT−4_1
MT−4_2

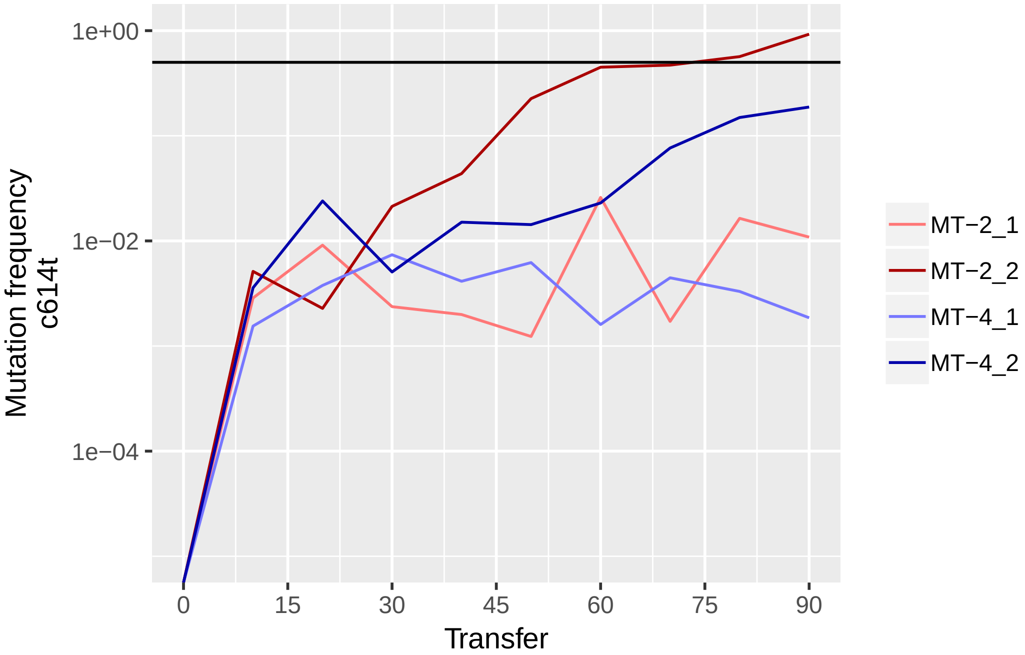

Mutation frequency
c614t
1e+00
1e−02
1e−04
0
15
30
45
60
75
90
Transfer
MT−2_1
MT−2_2
MT−4_1
MT−4_2

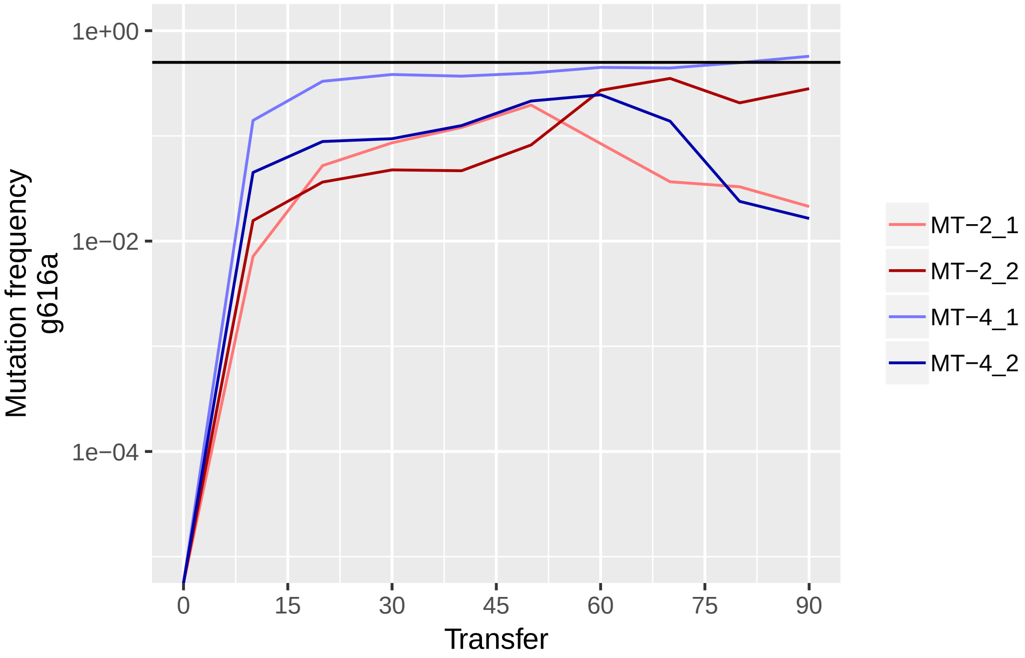

1e+00
1e−02
1e−04
Mutation frequency
g616a
0
15
30
45
60
75
90
Transfer
MT−2_1
MT−2_2
MT−4_1
MT−4_2

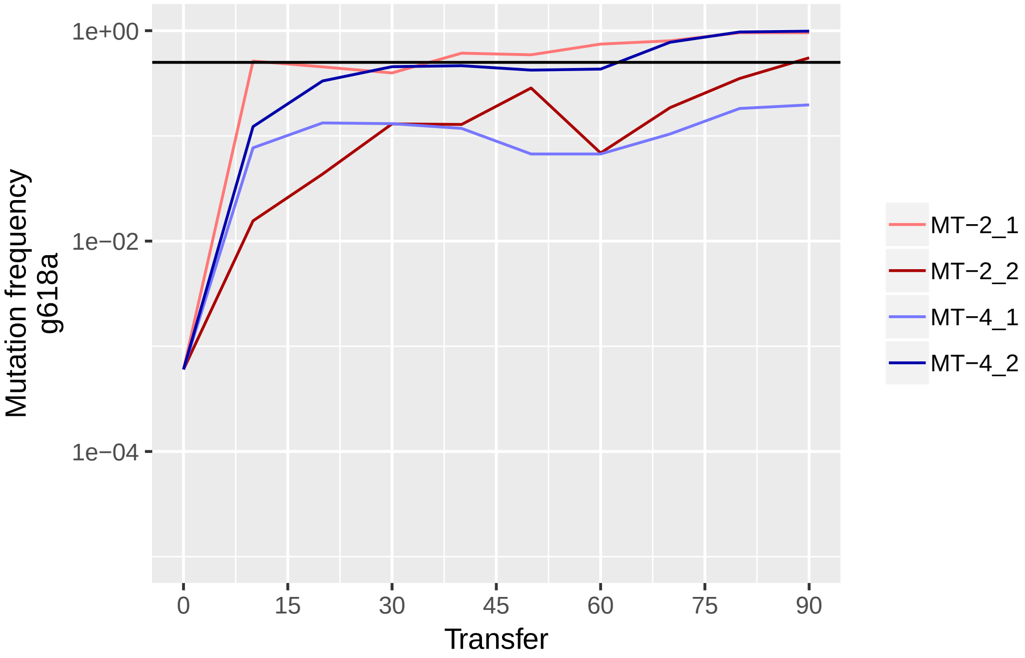
Mutation frequency
g618a
1e+00
1e−02
1e−04
0
15
30
45
60
75
90
Transfer
MT−2_1
MT−2_2
MT−4_1
MT−4_2

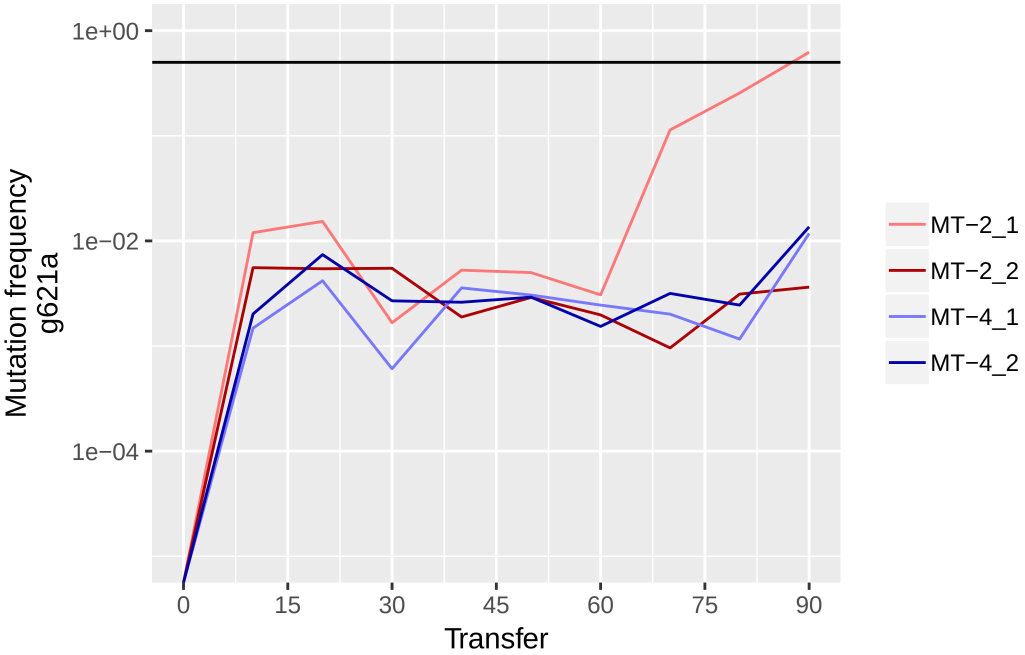

Mutation frequency
g621a
1e+00
1e−02
1e−04
0
15
30
45
60
75
90
Transfer
MT−2_1
MT−2_2
MT−4_1
MT−4_2

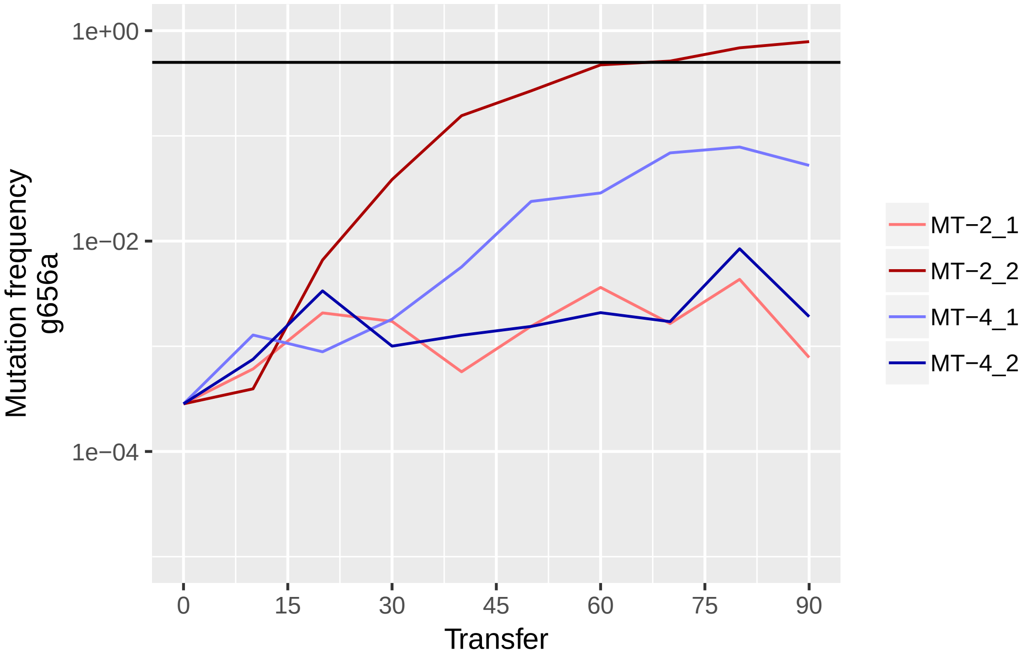

1e+00
1e−02
1e−04
Mutation frequency
g656a
0
15
30
45
60
75
90
Transfer
MT−2_1
MT−2_2
MT−4_1
MT−4_2

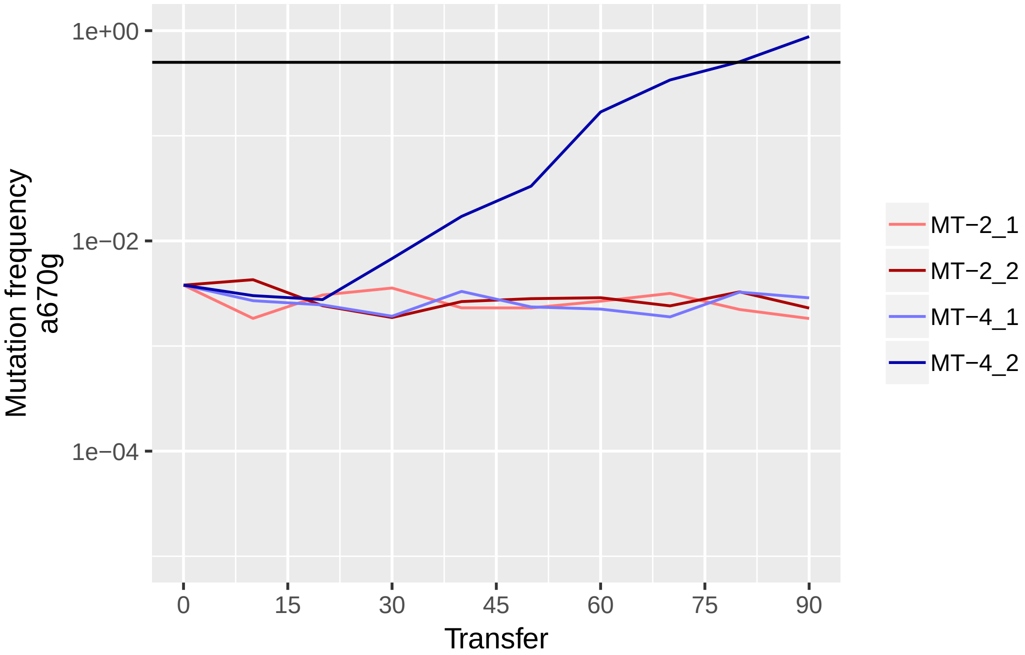

1e+00
1e−02
1e−04
Mutation frequency
a670g
0
15
30
45
60
75
90
Transfer
MT−2_1
MT−2_2
MT−4_1
MT−4_2

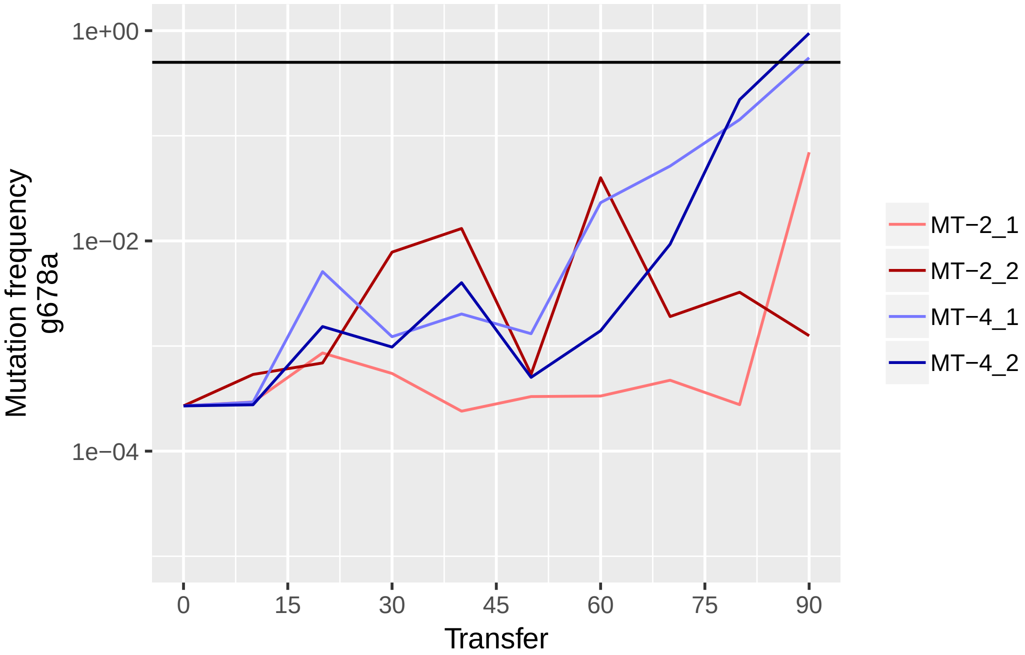

1e+00
1e−02
1e−04
Mutation frequency
g678a
0
15
30
45
60
75
90
Transfer
MT−2_1
MT−2_2
MT−4_1
MT−4_2

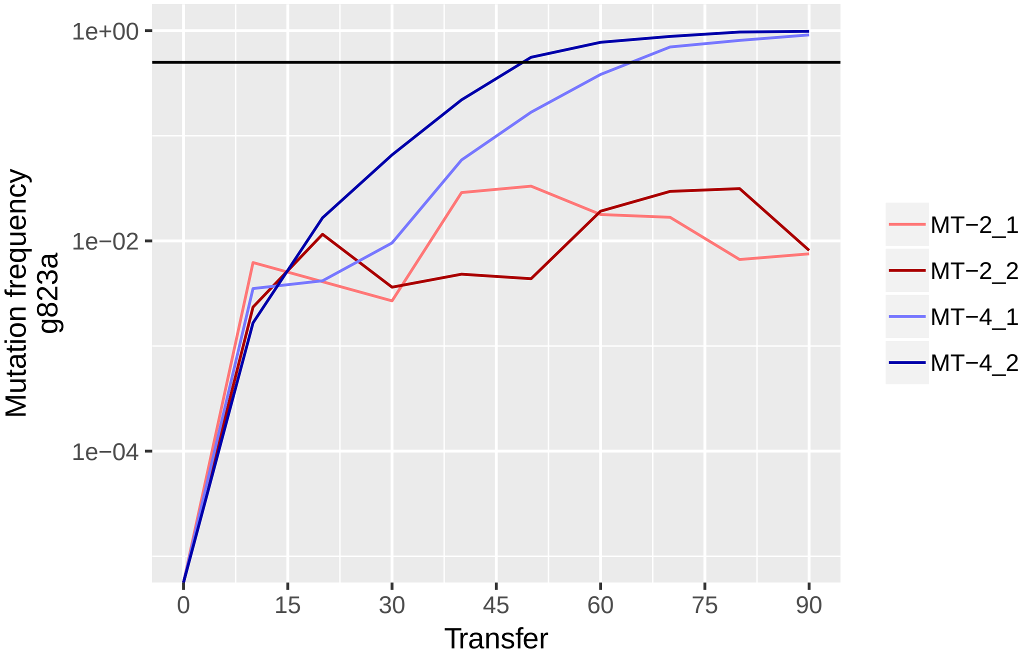

Mutation frequency
g823a
1e+00
1e−02
1e−04
0
15
30
45
60
75
90
Transfer
MT−2_1
MT−2_2
MT−4_1
MT−4_2

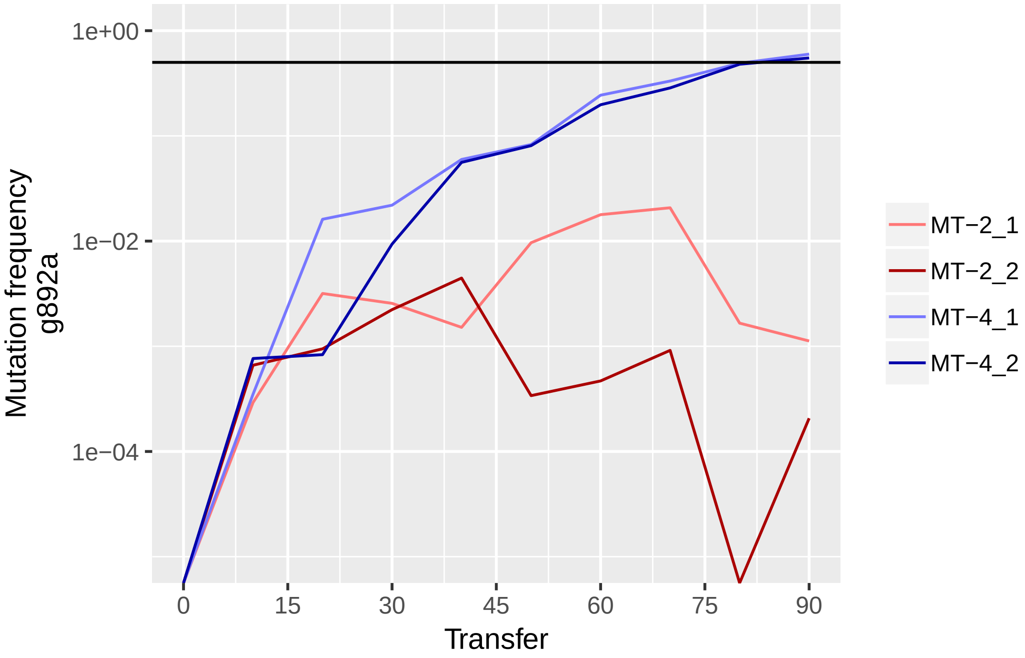

Mutation frequency
g892a
1e+00
1e−02
1e−04
0
15
30
45
60
75
90
Transfer
MT−2_1
MT−2_2
MT−4_1
MT−4_2

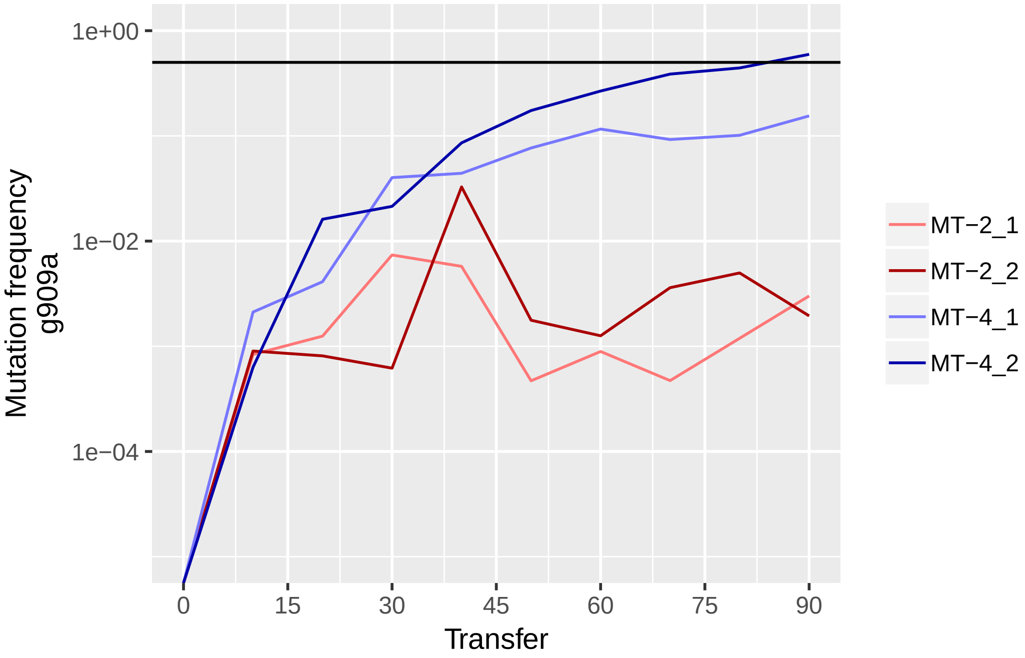

1e+00
1e−02
1e−04
Mutation frequency
g909a
0
15
30
45
60
75
90
Transfer
MT−2_1
MT−2_2
MT−4_1
MT−4_2

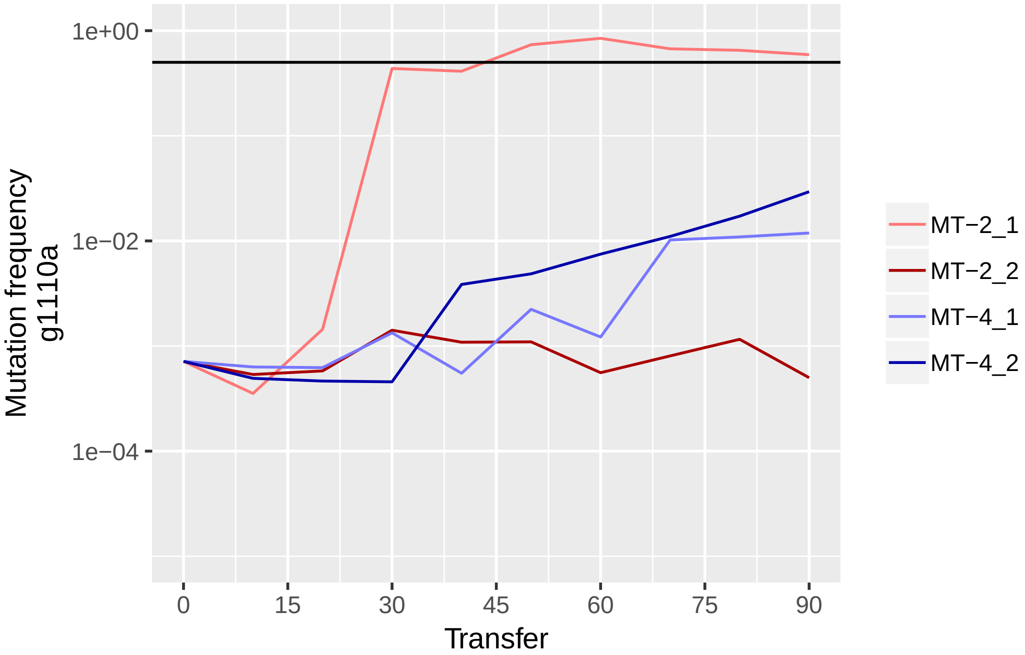

1e+00
1e−02
1e−04
Mutation frequency
g1110a
0
15
30
45
60
75
90
Transfer
MT−2_1
MT−2_2
MT−4_1
MT−4_2

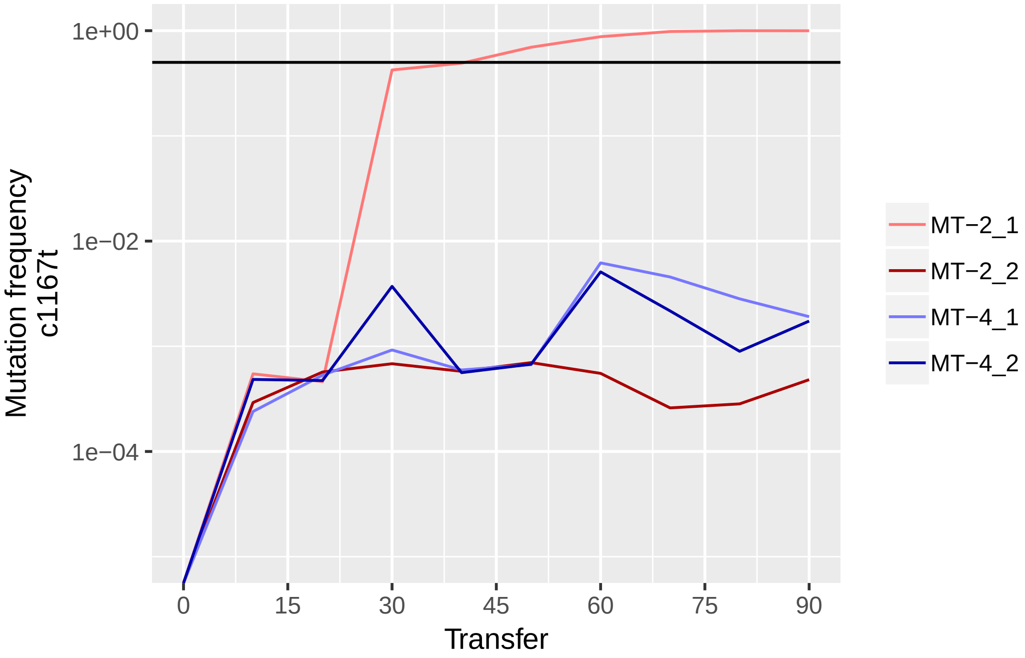

Mutation frequency
c1167t
1e+00
1e−02
1e−04
0
15
30
45
60
75
90
Transfer
MT−2_1
MT−2_2
MT−4_1
MT−4_2

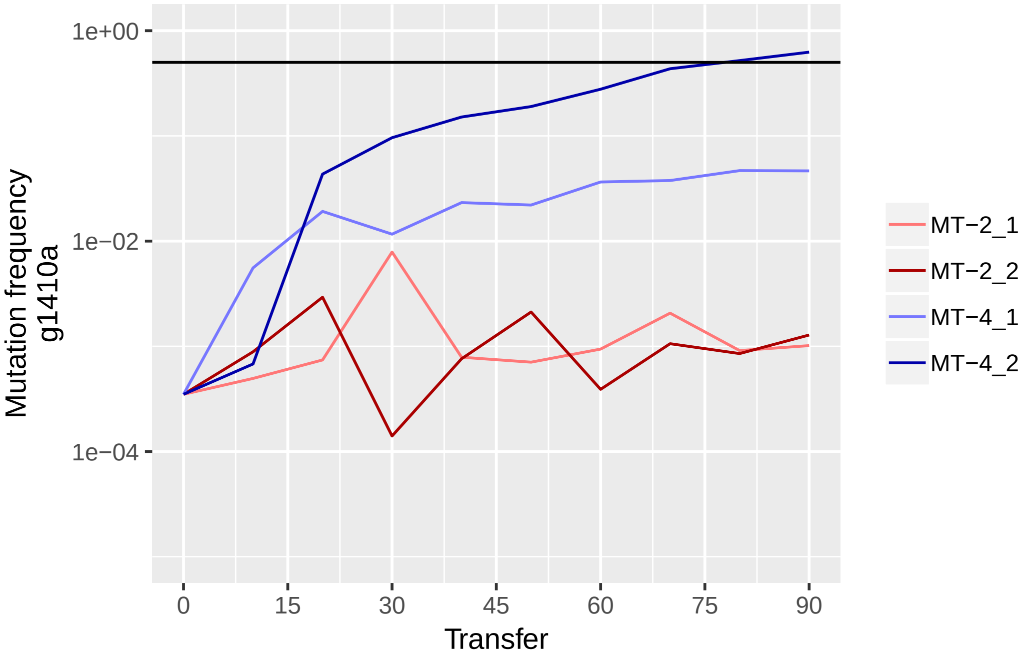

1e+00
1e−02
1e−04
Mutation frequency
g1410a
0
15
30
45
60
75
90
Transfer
MT−2_1
MT−2_2
MT−4_1
MT−4_2

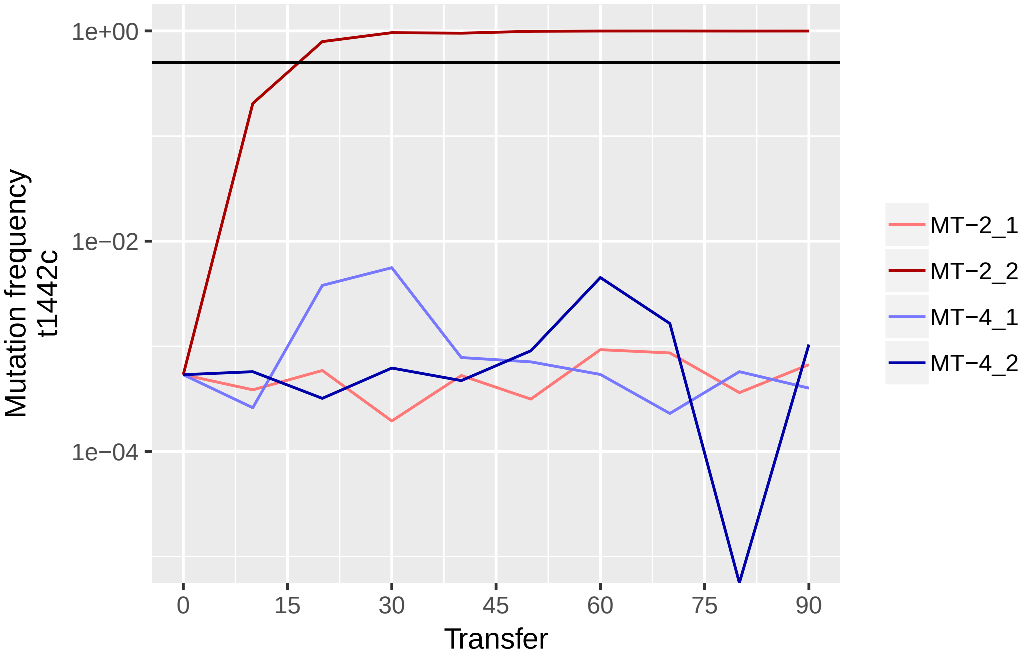

1e+00
1e−02
1e−04
Mutation frequency
t1442c
0
15
30
45
60
75
90
Transfer
MT−2_1
MT−2_2
MT−4_1
MT−4_2

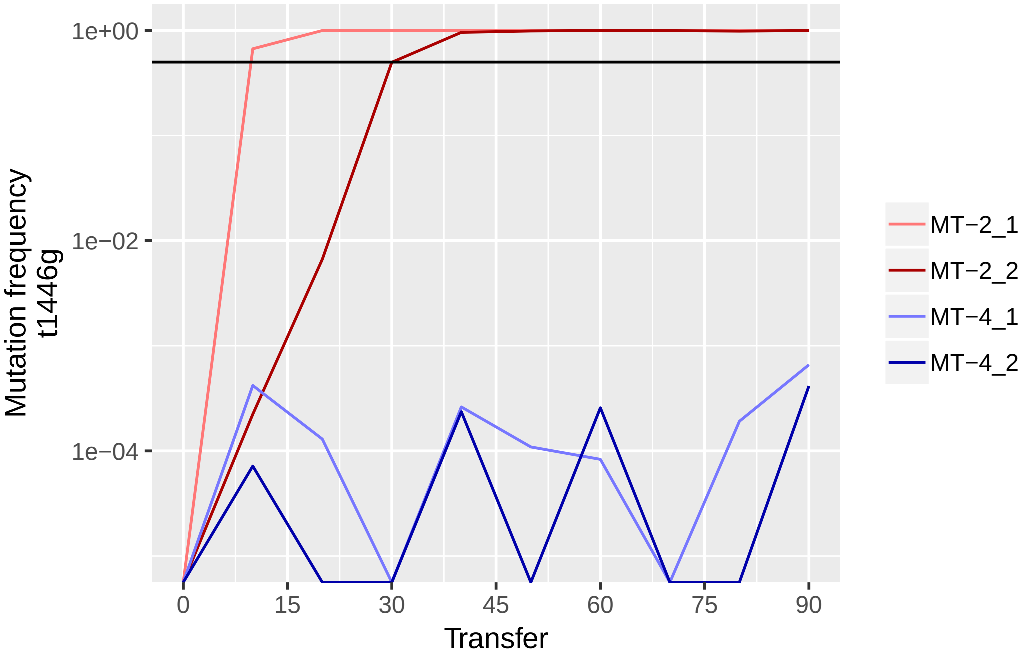

1e+00
1e−02
1e−04
Mutation frequency
t1446g
0
15
30
45
60
75
90
Transfer
MT−2_1
MT−2_2
MT−4_1
MT−4_2

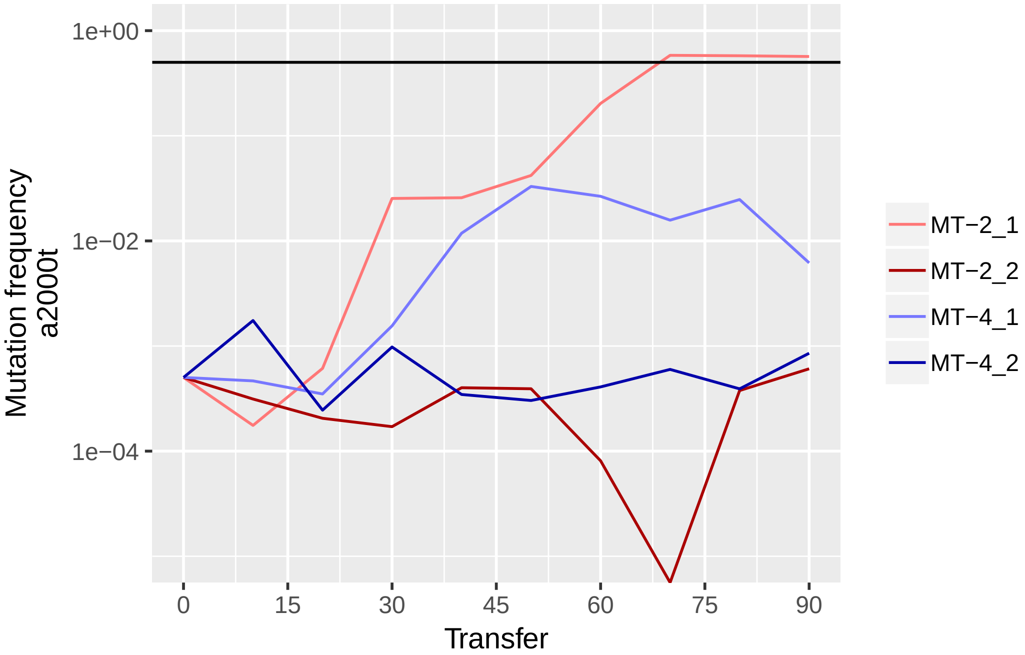

1e+00
1e−02
1e−04
Mutation frequency
a2000t
0
15
30
45
60
75
90
Transfer
MT−2_1
MT−2_2
MT−4_1
MT−4_2

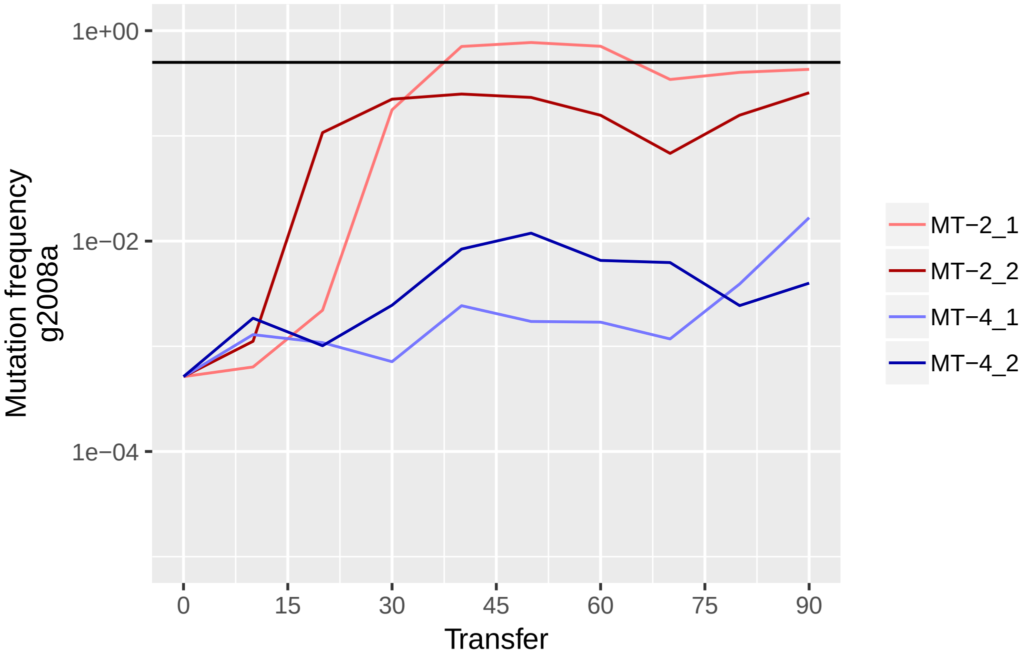

1e+00
1e−02
1e−04
Mutation frequency
g2008a
0
15
30
45
60
75
90
Transfer
MT−2_1
MT−2_2
MT−4_1
MT−4_2

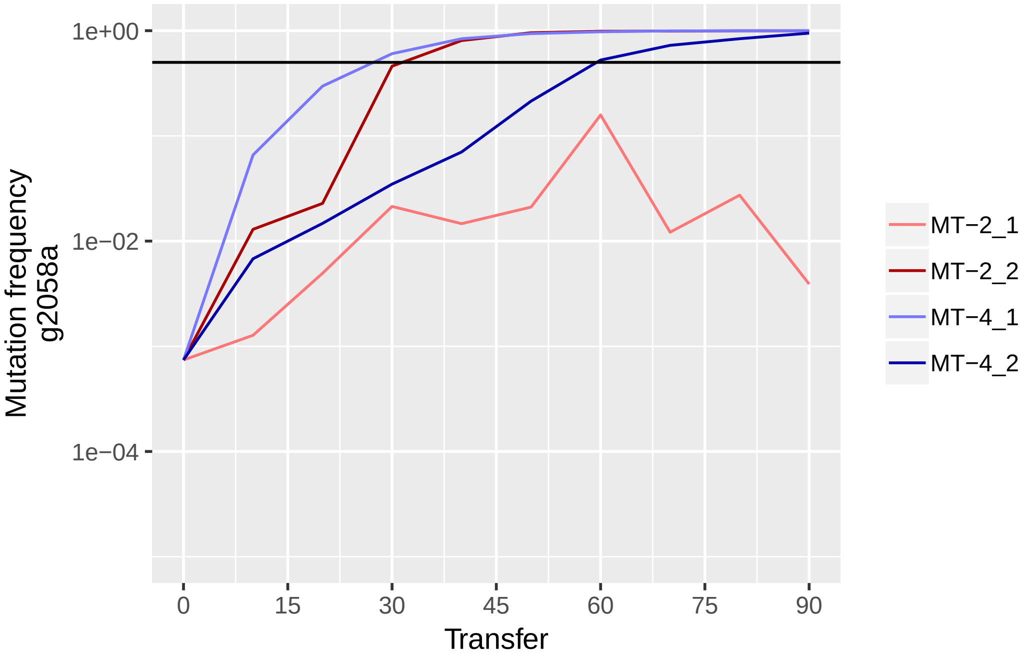

1e+00
1e−02
1e−04
Mutation frequency
g2058a
0
15
30
45
60
75
90
Transfer
MT−2_1
MT−2_2
MT−4_1
MT−4_2

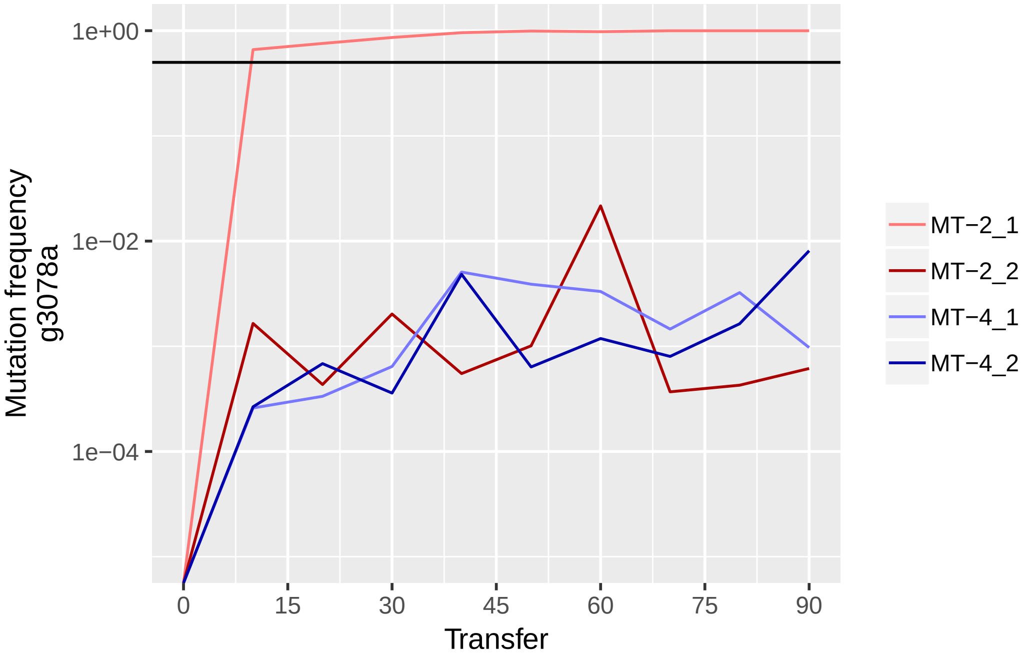

1e+00
1e−02
1e−04
Mutation frequency
g3078a
0
15
30
45
60
75
90
Transfer
MT−2_1
MT−2_2
MT−4_1
MT−4_2

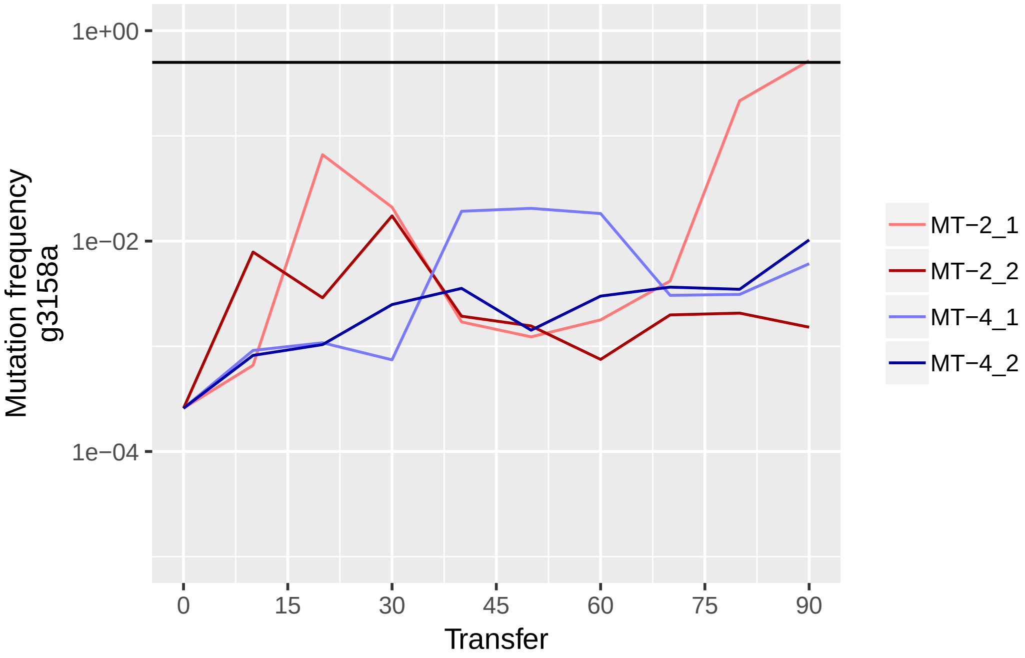

1e+00
1e−02
1e−04
Mutation frequency
g3158a
0
15
30
45
60
75
90
Transfer
MT−2_1
MT−2_2
MT−4_1
MT−4_2

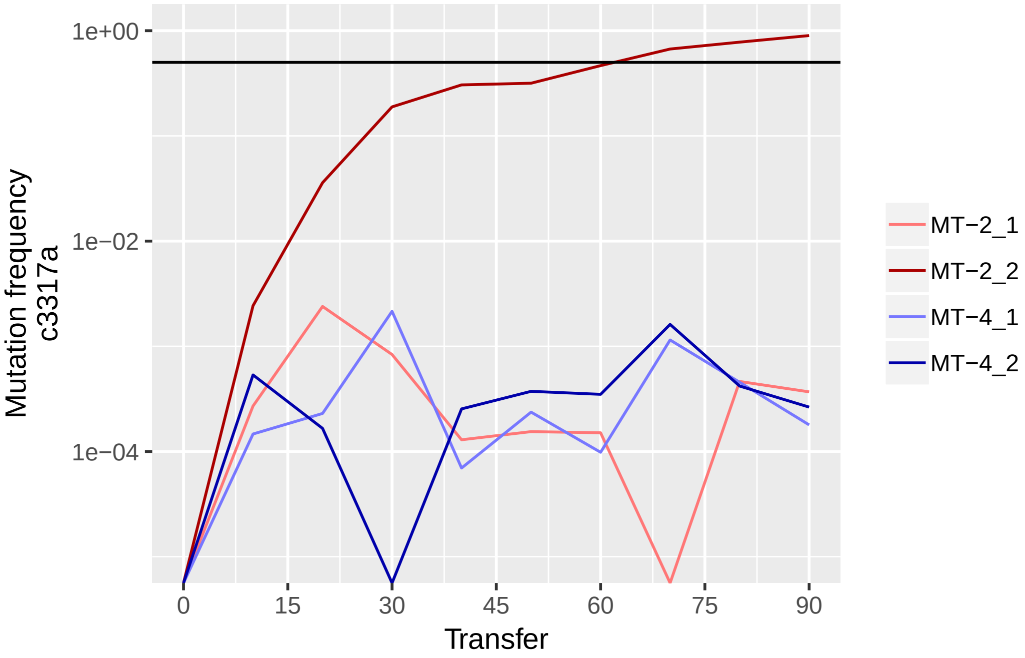

Mutation frequency
c3317a
1e+00
1e−02
1e−04
0
15
30
45
60
75
90
Transfer
MT−2_1
MT−2_2
MT−4_1
MT−4_2

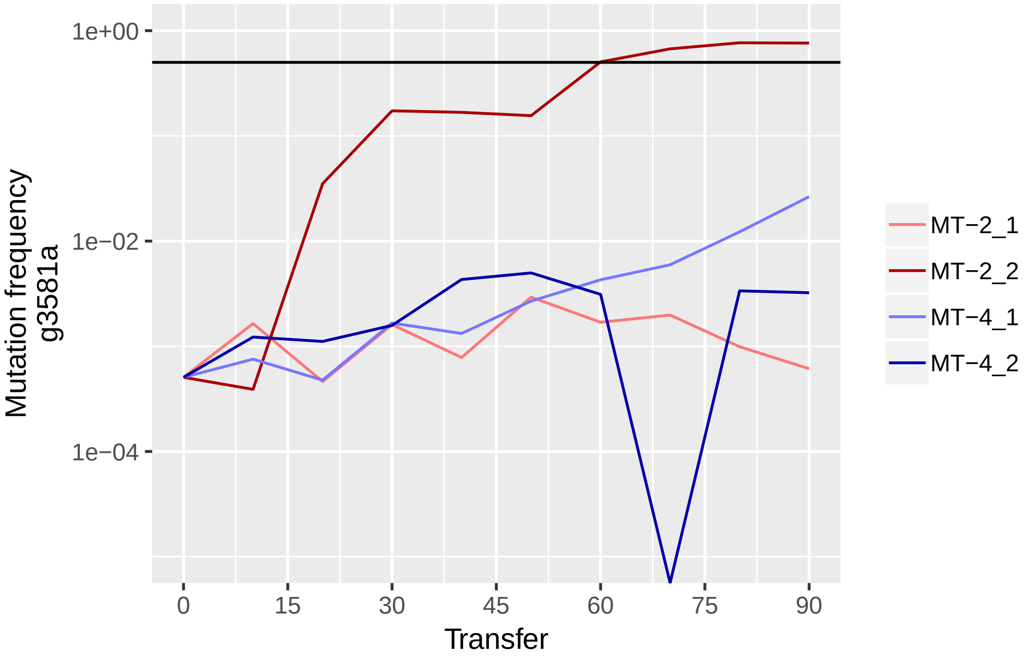

1e+00
1e−02
1e−04
Mutation frequency
g3581a
0
15
30
45
60
75
90
Transfer
MT−2_1
MT−2_2
MT−4_1
MT−4_2

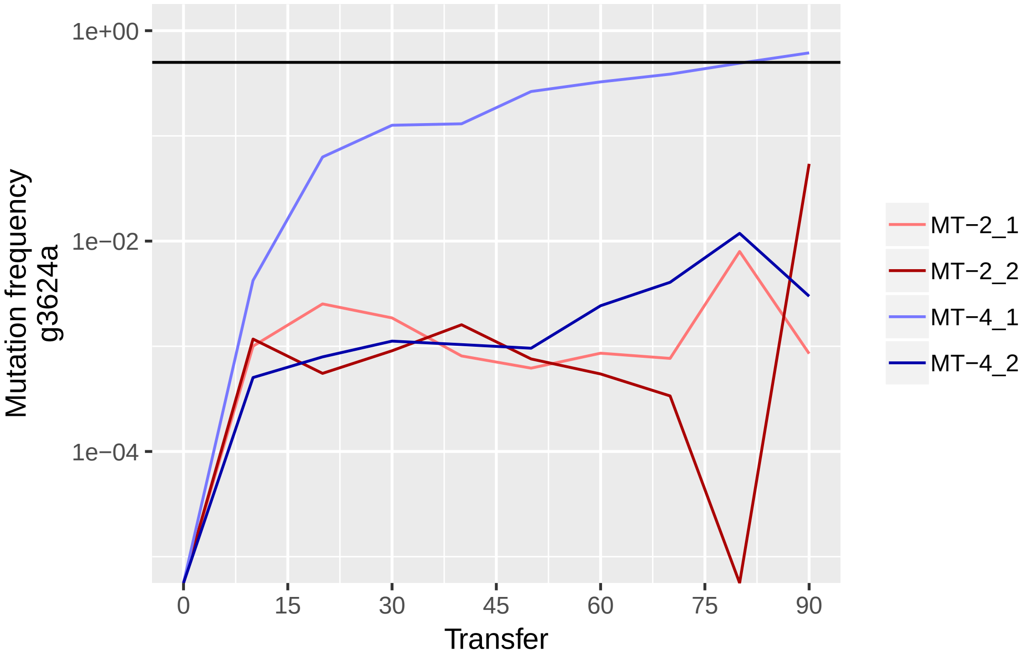

1e+00
1e−02
1e−04
Mutation frequency
g3624a
0
15
30
45
60
75
90
Transfer
MT−2_1
MT−2_2
MT−4_1
MT−4_2

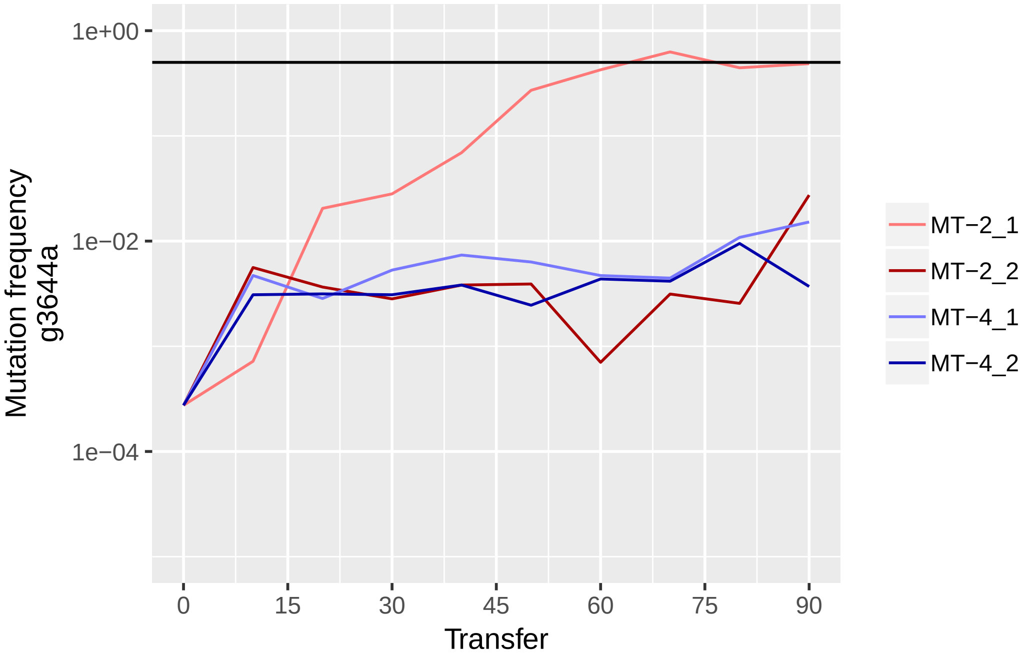

1e+00
1e−02
1e−04
Mutation frequency
g3644a
0
15
30
45
60
75
90
Transfer
MT−2_1
MT−2_2
MT−4_1
MT−4_2

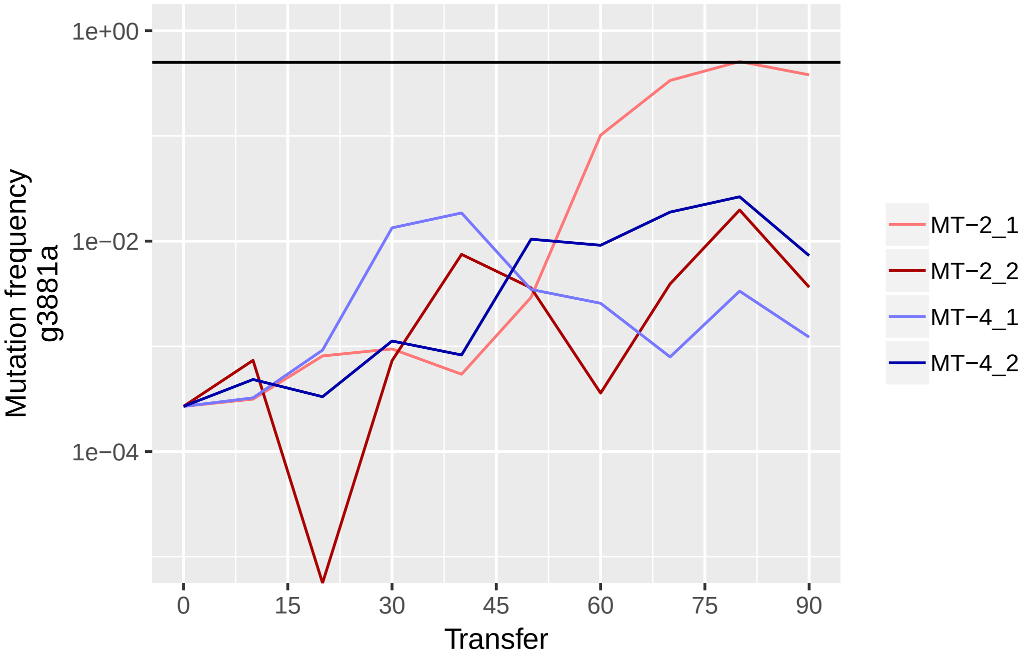

1e+00
1e−02
1e−04
Mutation frequency
g3881a
0
15
30
45
60
75
90
Transfer
MT−2_1
MT−2_2
MT−4_1
MT−4_2

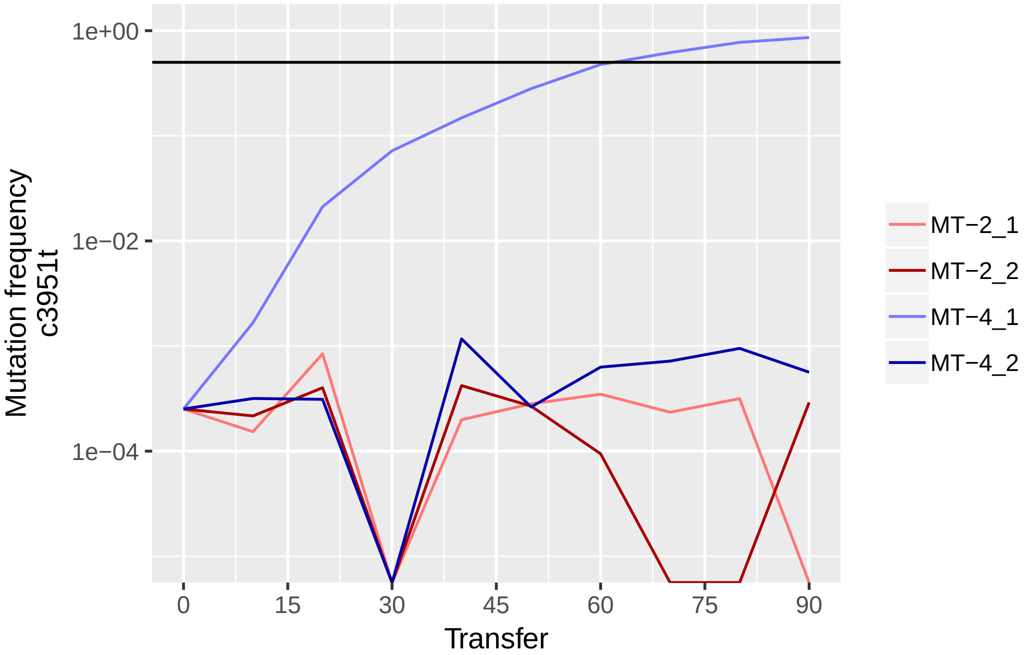

1e+00
1e−02
1e−04
Mutation frequency
c3951t
0
15
30
45
60
75
90
Transfer
MT−2_1
MT−2_2
MT−4_1
MT−4_2

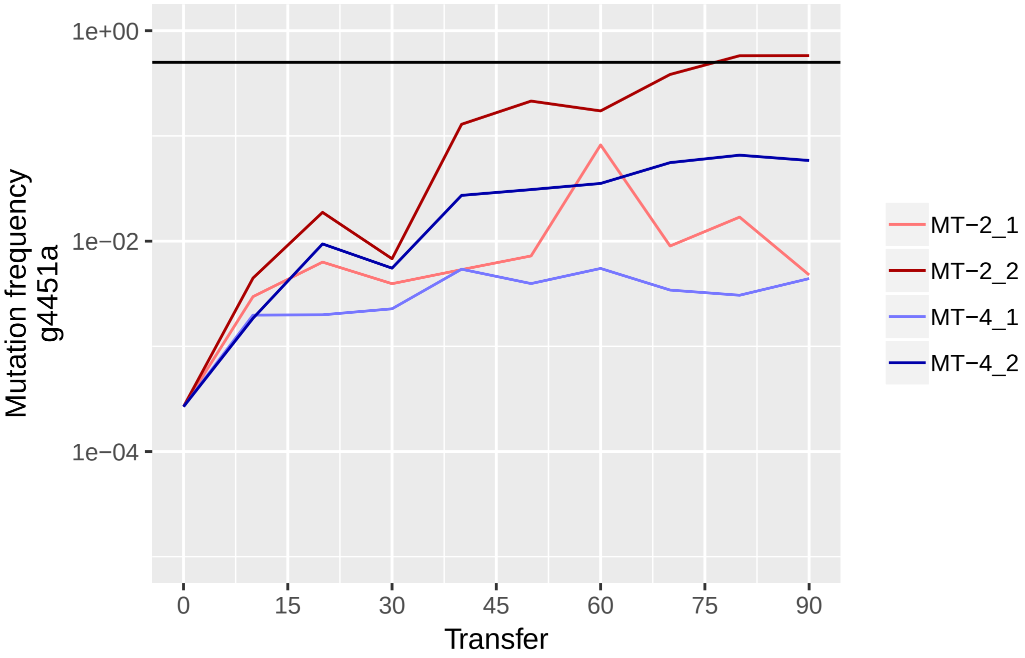

1e+00
1e−02
1e−04
Mutation frequency
g4451a
0
15
30
45
60
75
90
Transfer
MT−2_1
MT−2_2
MT−4_1
MT−4_2

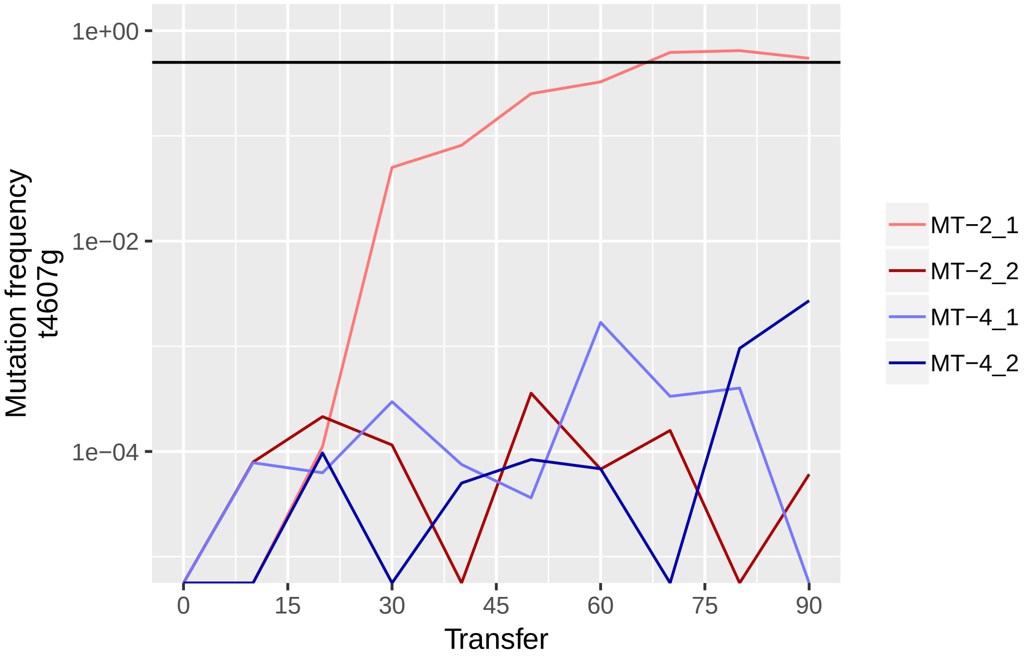

1e+00
1e−02
1e−04
Mutation frequency
t4607g
0
15
30
45
60
75
90
Transfer
MT−2_1
MT−2_2
MT−4_1
MT−4_2

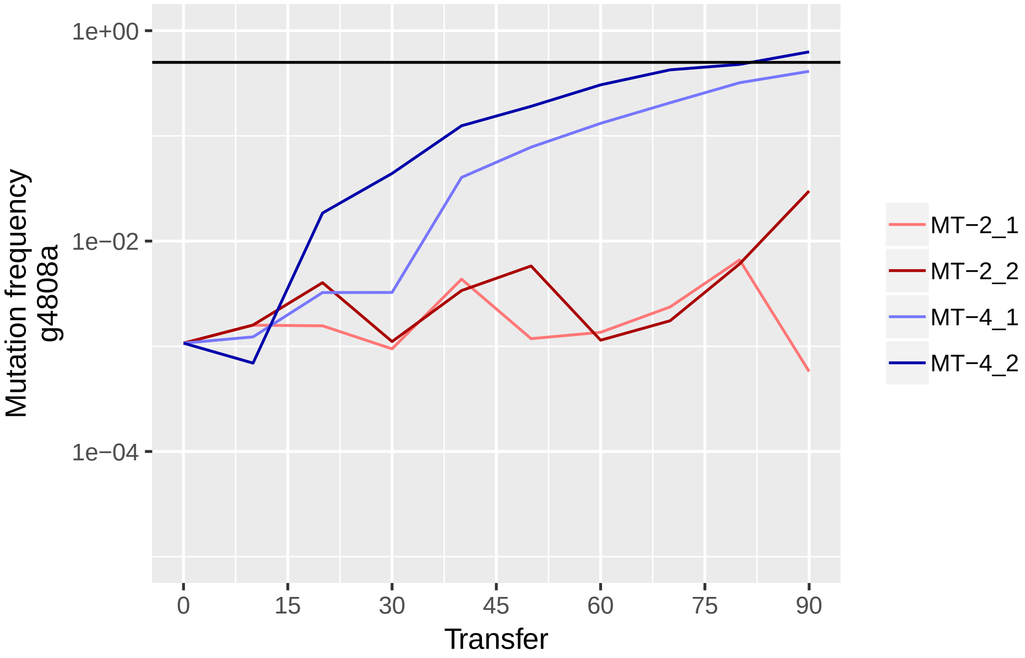
1e+00
1e−02
1e−04
Mutation frequency
g4808a
0
15
30
45
60
75
90
Transfer
MT−2_1
MT−2_2
MT−4_1
MT−4_2

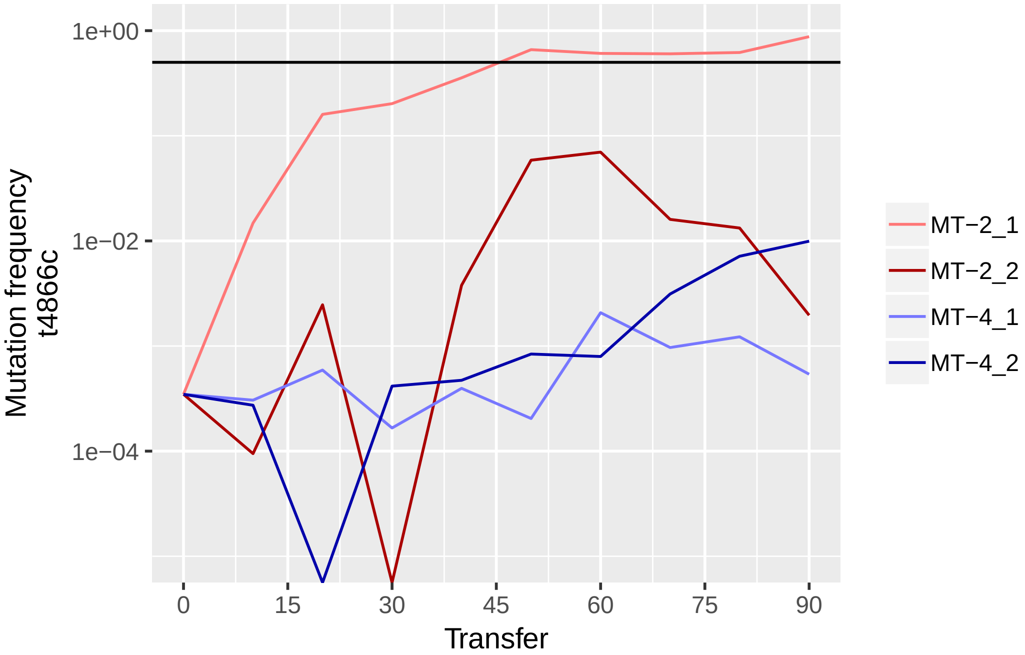
1e+00
1e−02
1e−04
Mutation frequency
t4866c
0
15
30
45
60
75
90
Transfer
MT−2_1
MT−2_2
MT−4_1
MT−4_2

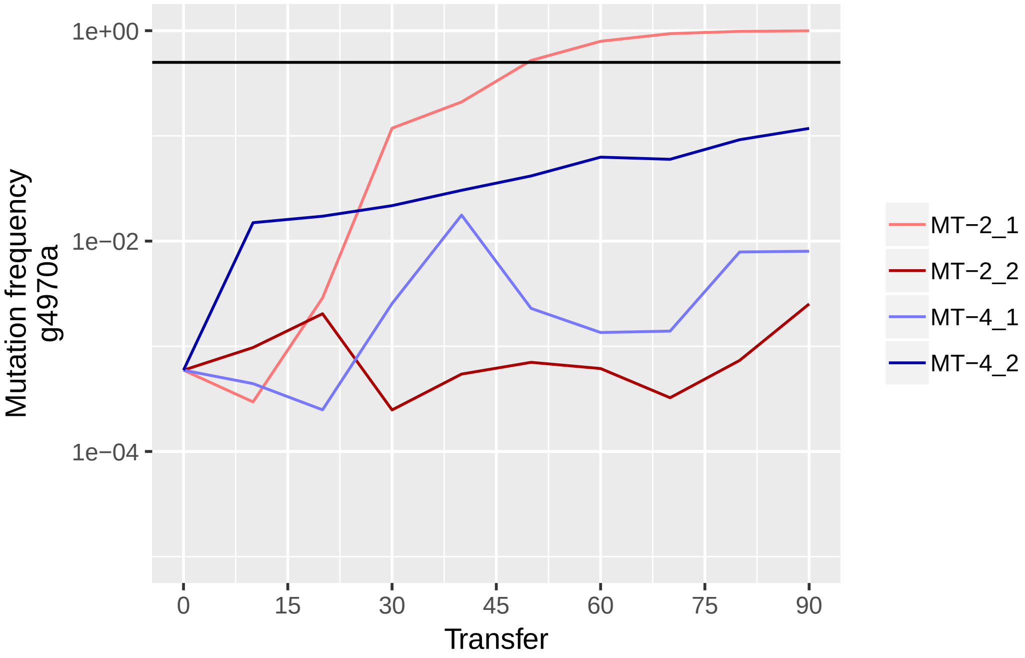

1e+00
1e−02
1e−04
0
15
30
45
60
75
90
Transfer
Mutation frequency
g4970a
MT−2_1
MT−2_2
MT−4_1
MT−4_2

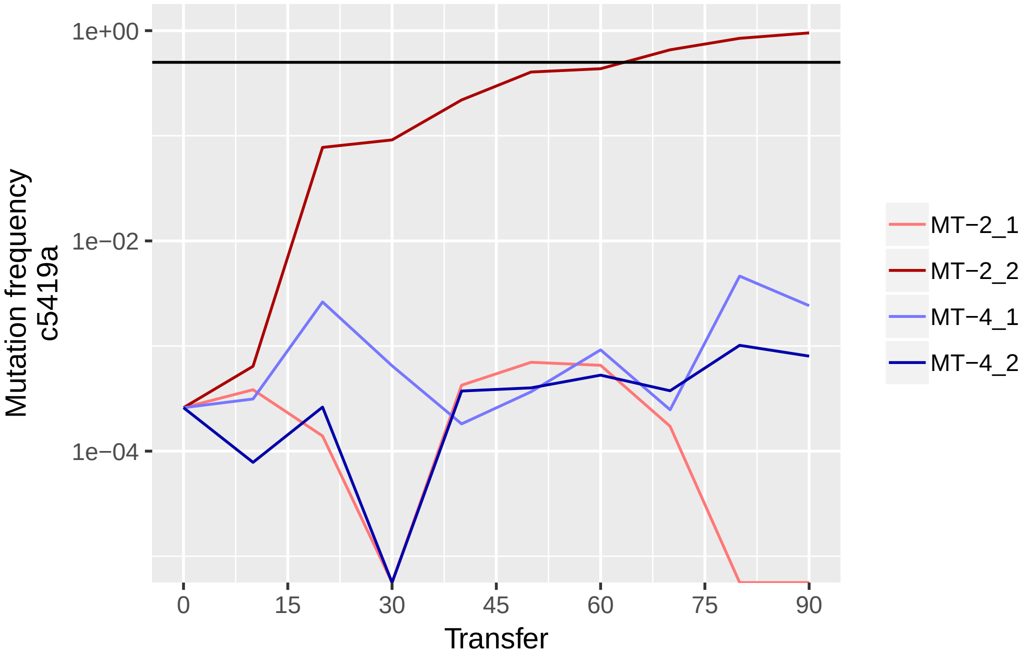

1e+00
1e−02
1e−04
Mutation frequency
c5419a
0
15
30
45
60
75
90
Transfer
MT−2_1
MT−2_2
MT−4_1
MT−4_2

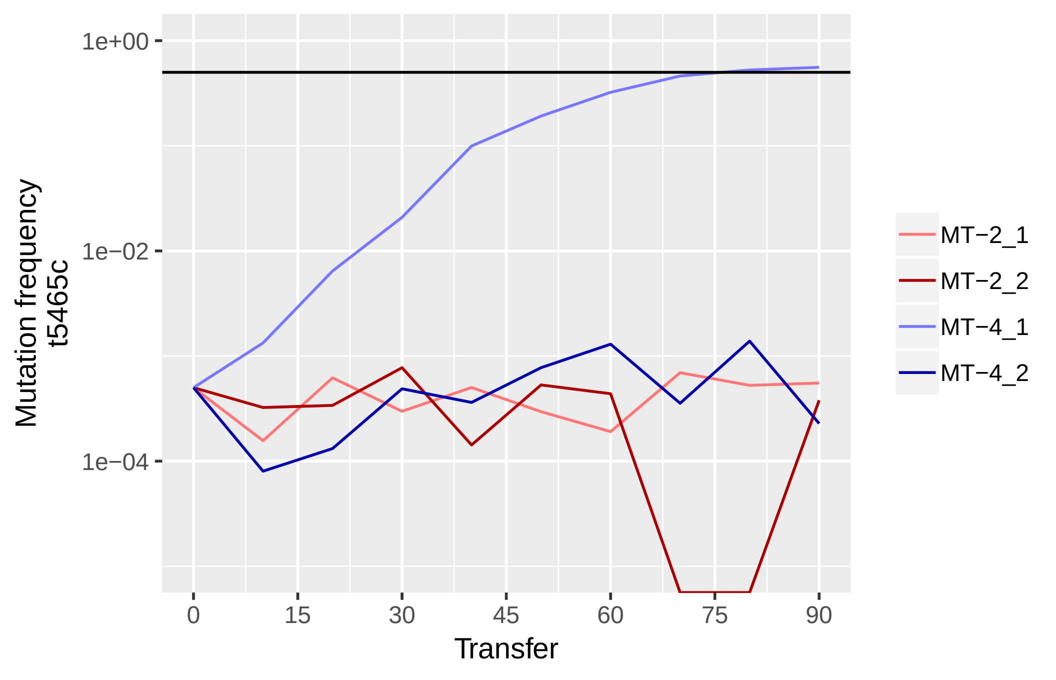

1e+00
1e−02
1e−04
Mutation frequency
t5465c
0
15
30
45
60
75
90
Transfer
MT−2_1
MT−2_2
MT−4_1
MT−4_2

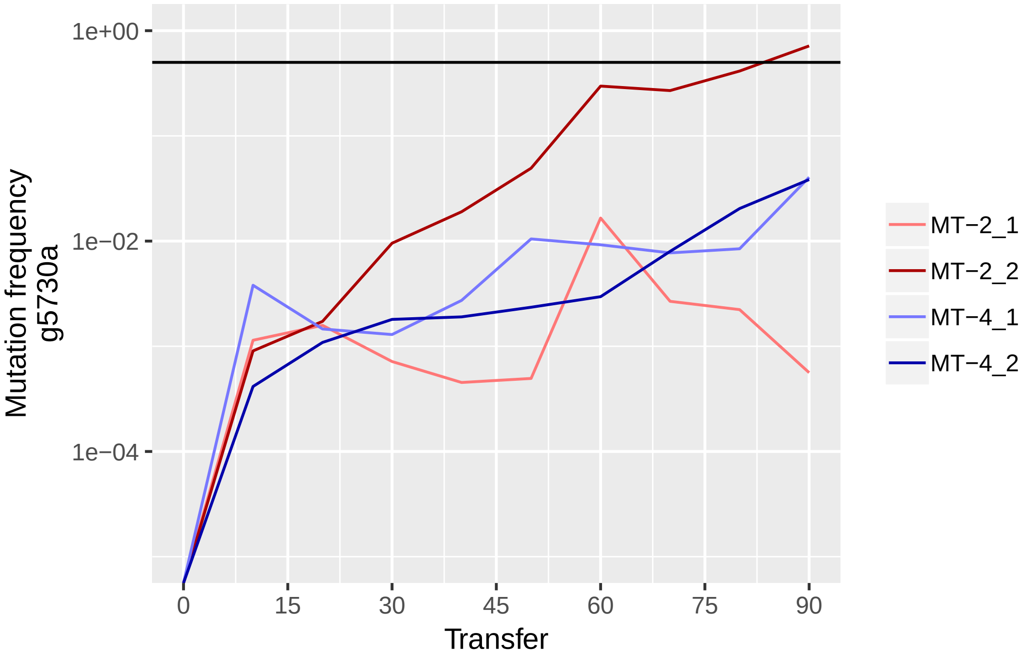

Mutation frequency
g5730a
1e+00
1e−02
1e−04
0
15
30
45
60
75
90
Transfer
MT−2_1
MT−2_2
MT−4_1
MT−4_2

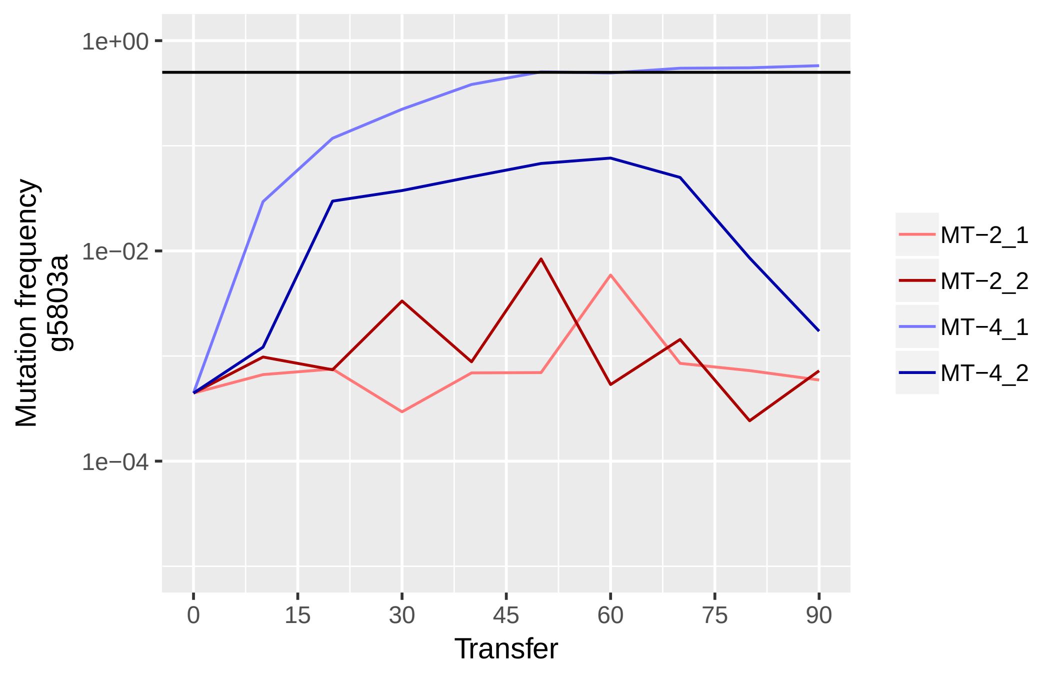

1e+00
1e−02
1e−04
Mutation frequency
g5803a
0
15
30
45
60
75
90
Transfer
MT−2_1
MT−2_2
MT−4_1
MT−4_2

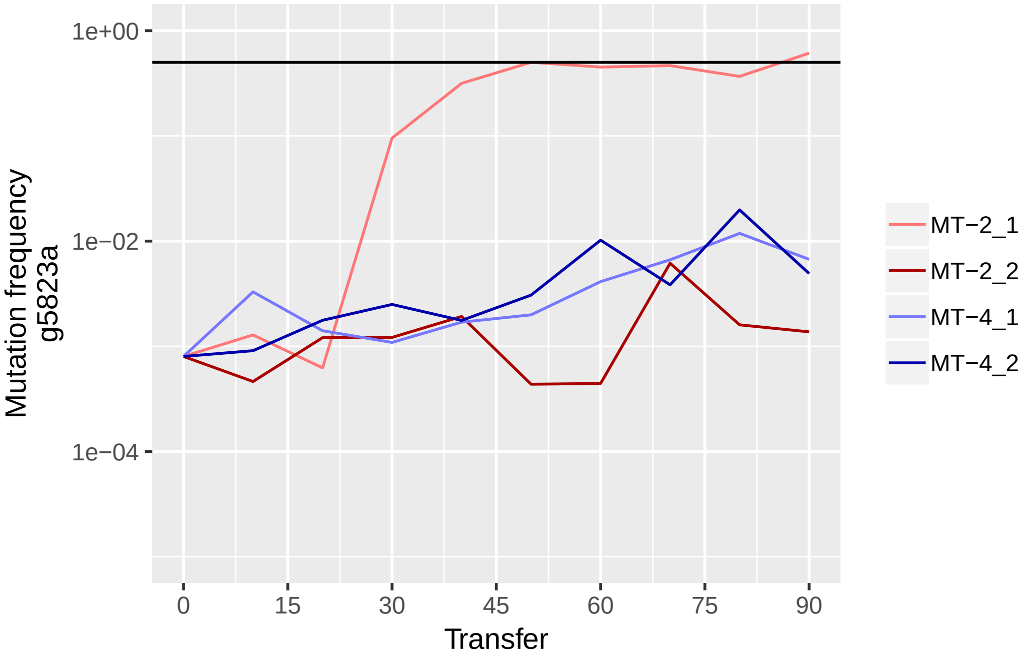
1e+00
1e−02
1e−04
Mutation frequency
g5823a
0
15
30
45
60
75
90
Transfer
MT−2_1
MT−2_2
MT−4_1
MT−4_2

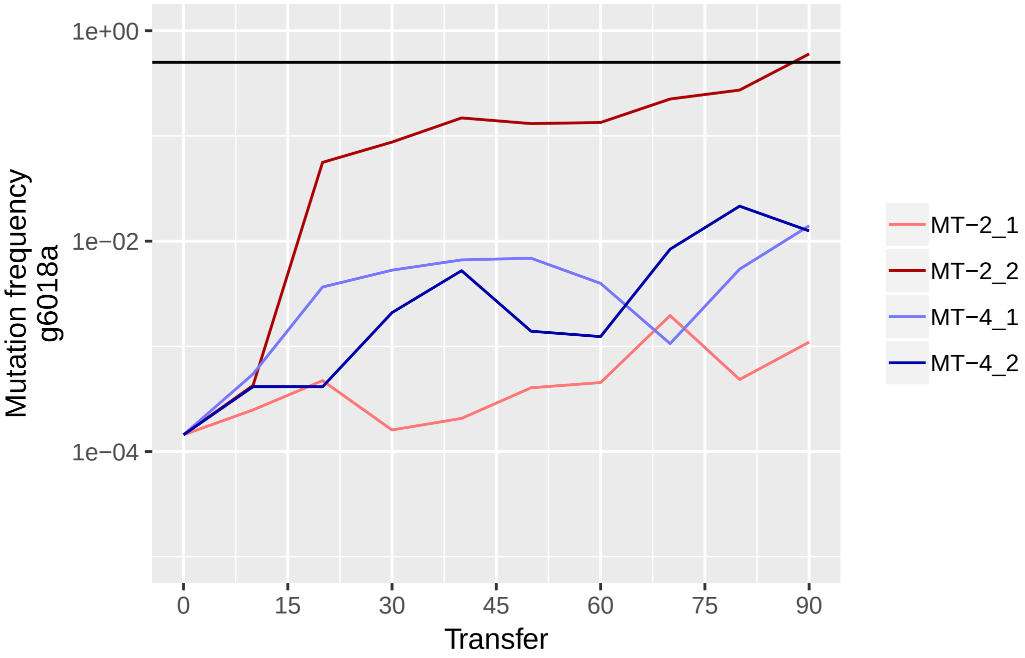

1e+00
1e−02
1e−04
Mutation frequency
g6018a
0
15
30
45
60
75
90
Transfer
MT−2_1
MT−2_2
MT−4_1
MT−4_2

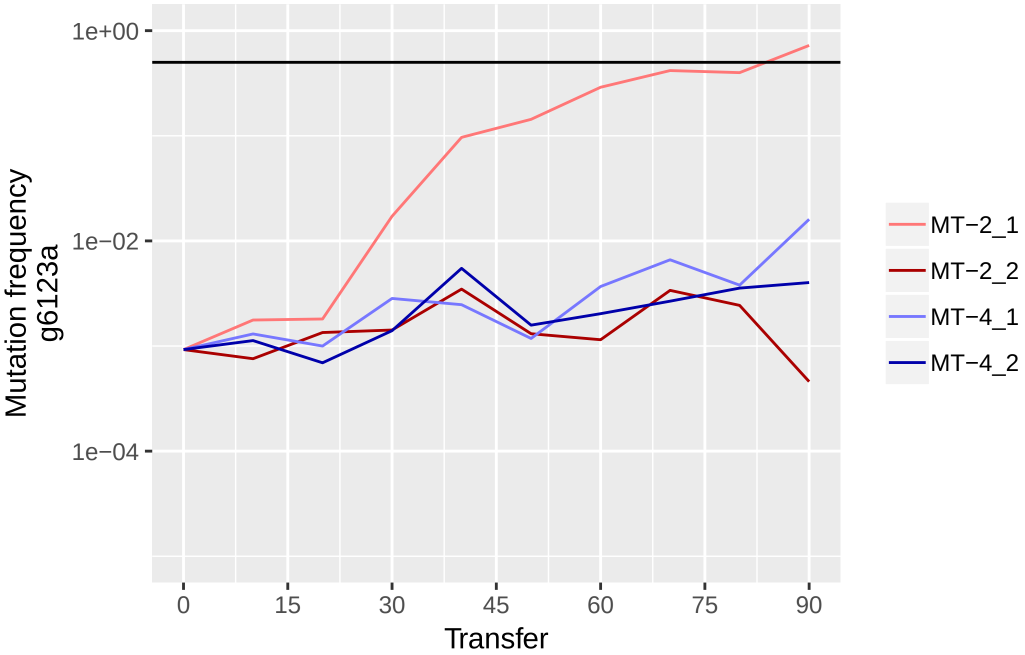

1e+00
1e−02
1e−04
Mutation frequency
g6123a
0
15
30
45
60
75
90
Transfer
MT−2_1
MT−2_2
MT−4_1
MT−4_2

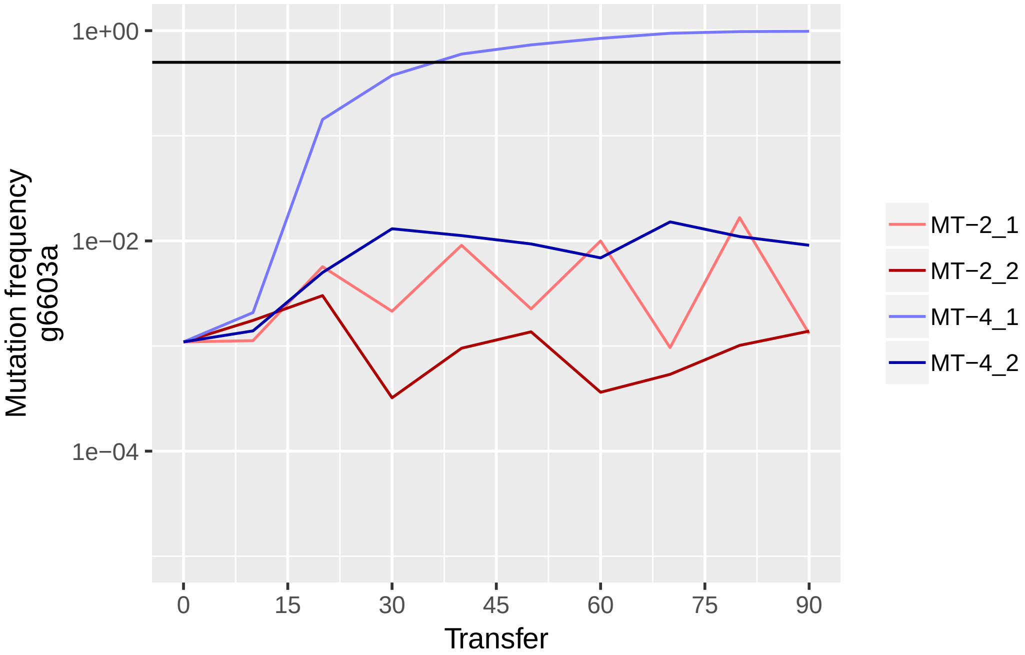

1e+00
1e−02
1e−04
Mutation frequency
g6603a
0
15
30
45
60
75
90
Transfer
MT−2_1
MT−2_2
MT−4_1
MT−4_2

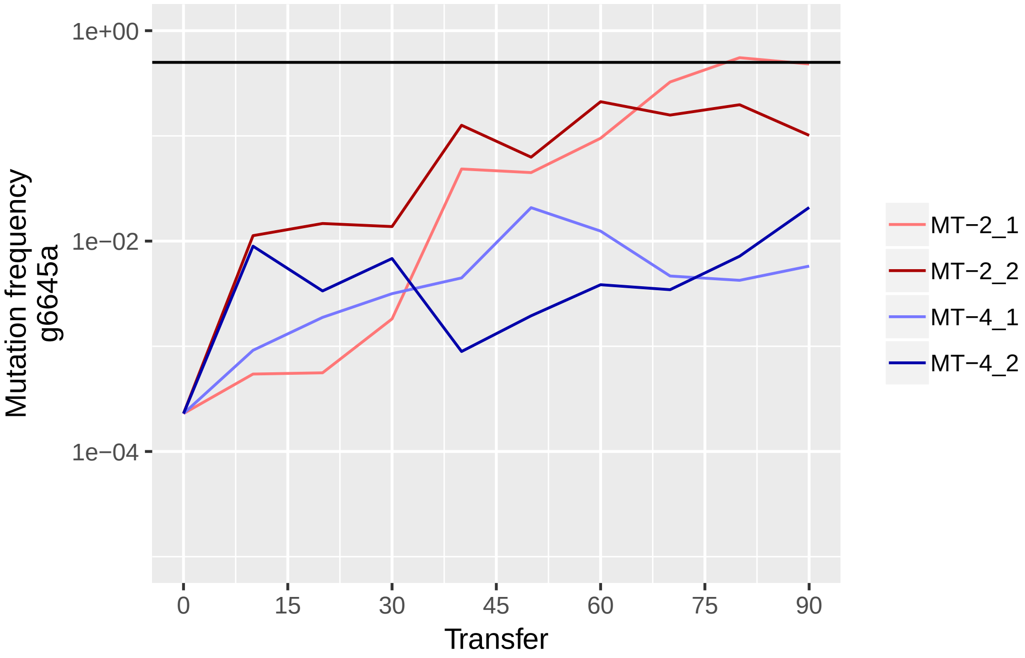

Mutation frequency
g6645a
1e+00
1e−02
1e−04
0
15
30
45
60
75
90
Transfer
MT−2_1
MT−2_2
MT−4_1
MT−4_2

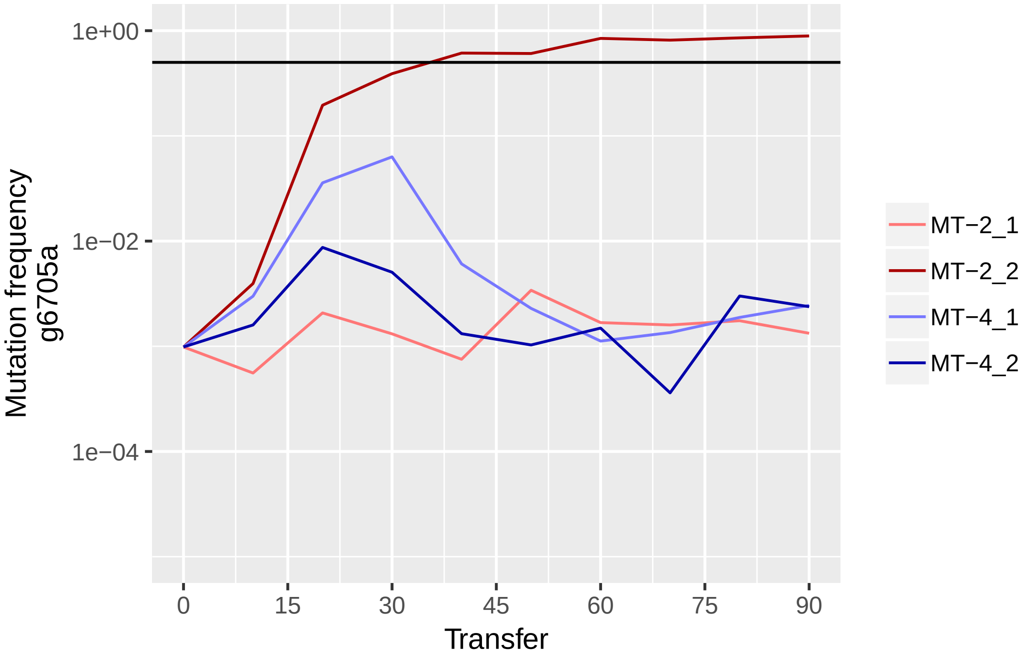

1e+00
1e−02
1e−04
Mutation frequency
g6705a
0
15
30
45
60
75
90
Transfer
MT−2_1
MT−2_2
MT−4_1
MT−4_2

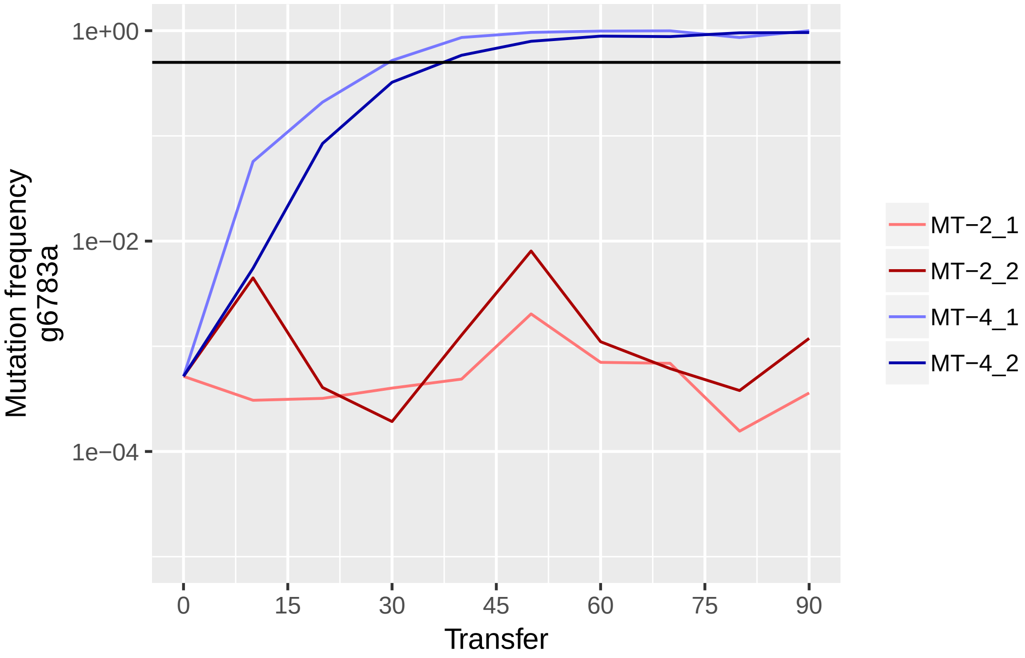
1e+00
1e−02
1e−04
Mutation frequency
g6783a
0
15
30
45
60
75
90
Transfer
MT−2_1
MT−2_2
MT−4_1
MT−4_2

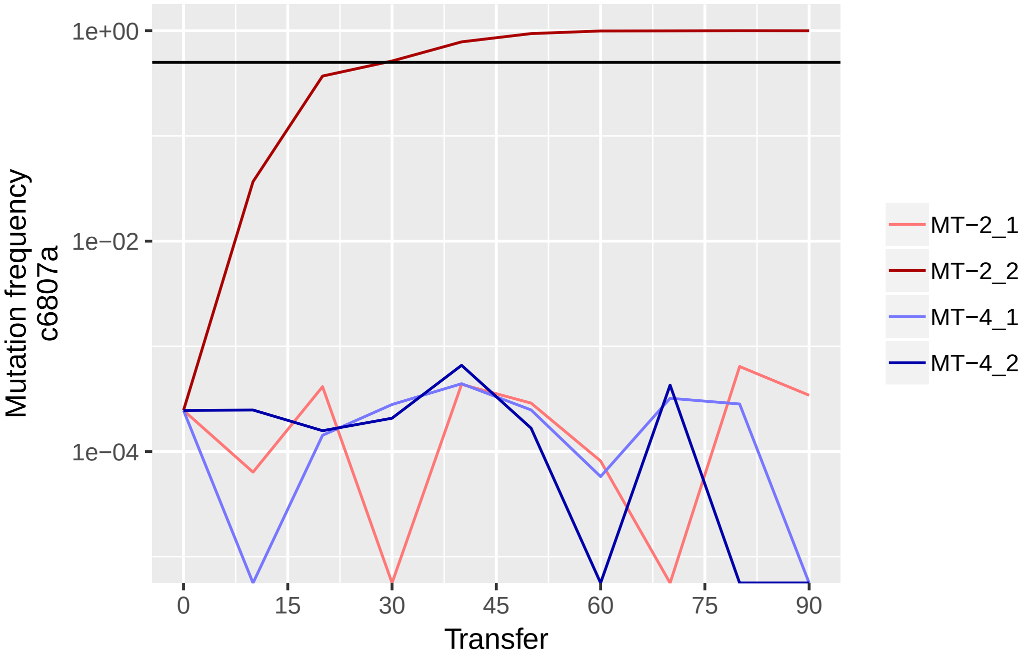

1e+00
1e−02
1e−04
Mutation frequency
c6807a
0
15
30
45
60
75
90
Transfer
MT−2_1
MT−2_2
MT−4_1
MT−4_2

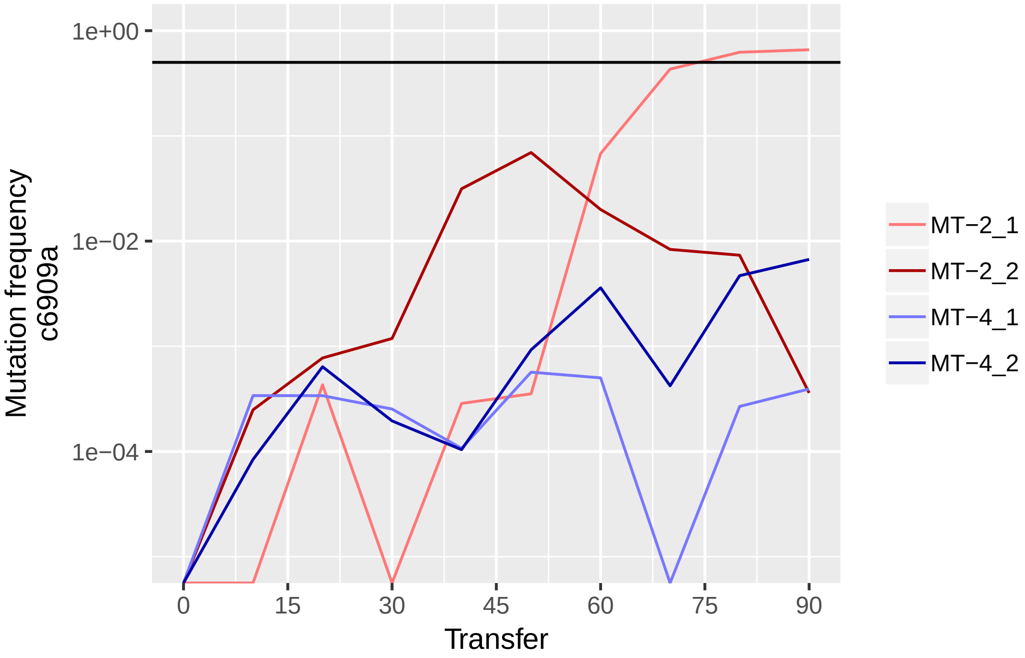
Mutation frequency
c6909a
1e+00
1e−02
1e−04
0
15
30
45
60
75
90
Transfer
MT−2_1
MT−2_2
MT−4_1
MT−4_2

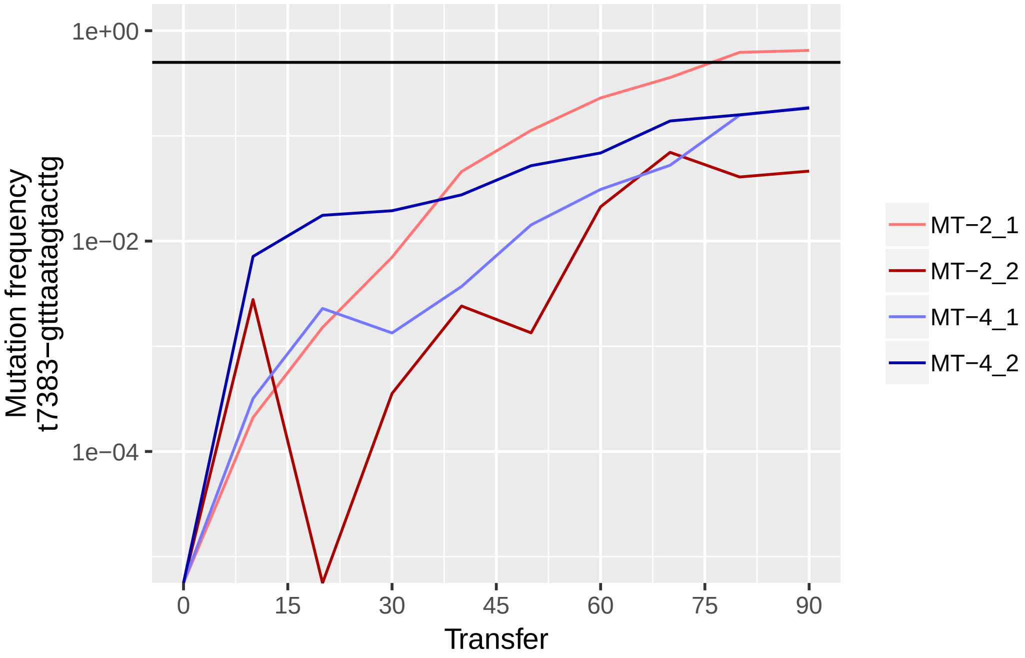

Mutation frequency
t7383−gtttaatagtactg
1e+00
1e−02
1e−04
0
15
30
45
60
75
90
Transfer
MT−2_1
MT−2_2
MT−4_1
MT−4_2

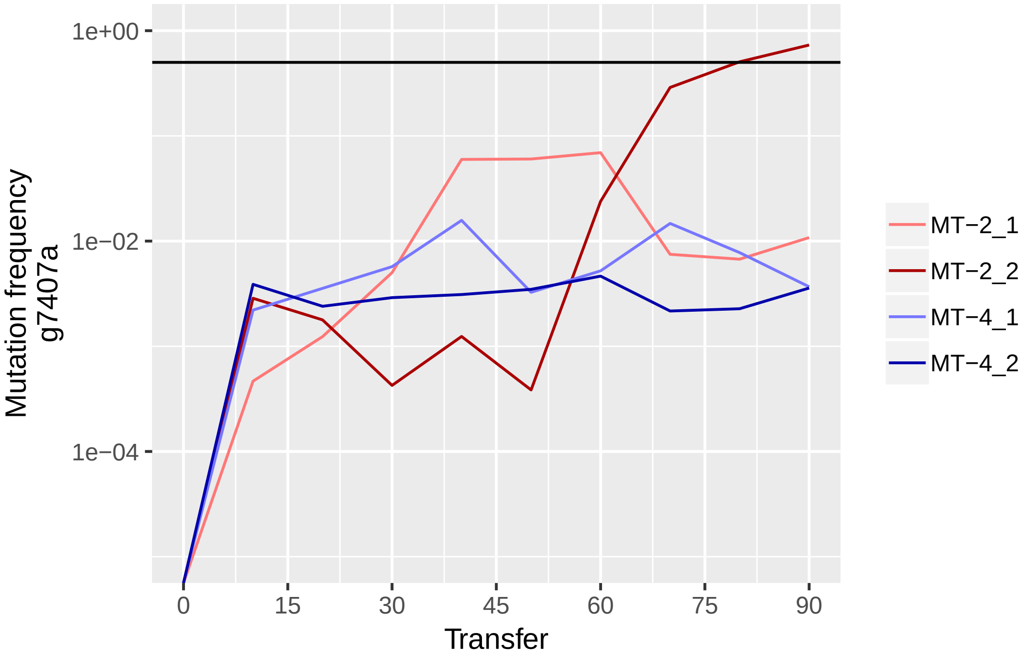

Mutation frequency
g7407a
1e+00
1e−02
1e−04
0
15
30
45
60
75
90
Transfer
MT−2_1
MT−2_2
MT−4_1
MT−4_2

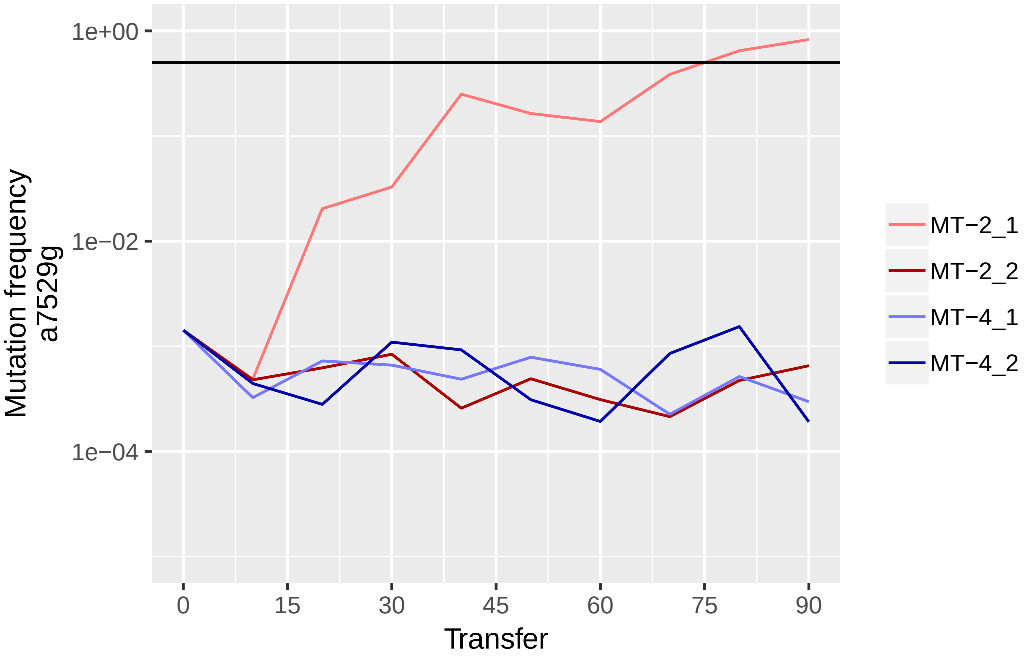
1e+00
1e−02
1e−04
Mutation frequency
a7529g
0
15
30
45
60
75
90
Transfer
MT−2_1
MT−2_2
MT−4_1
MT−4_2

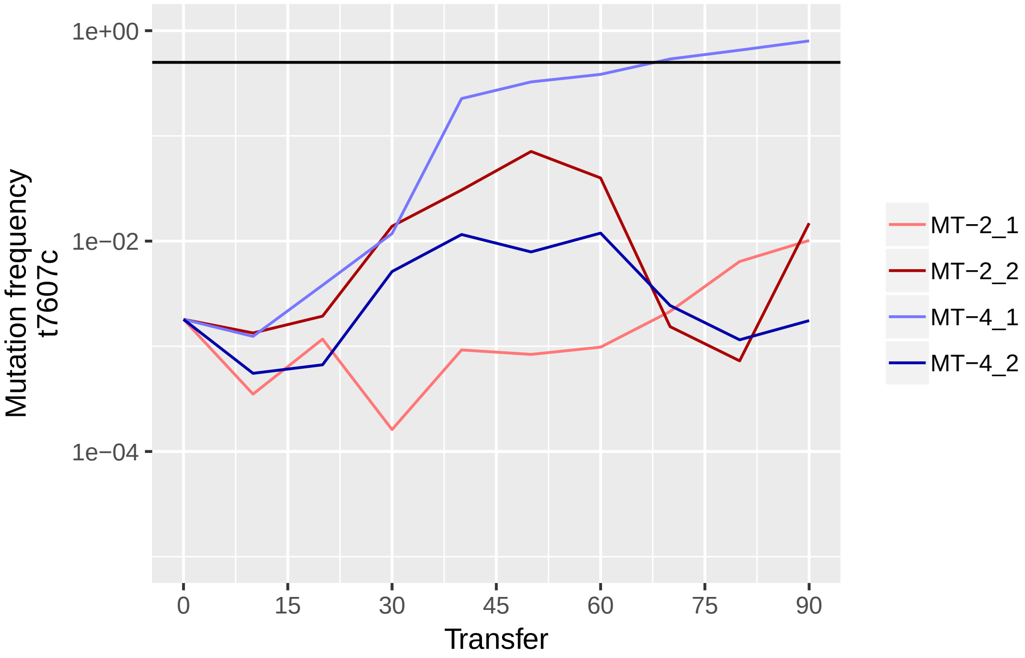

1e+00
1e−02
1e−04
Mutation frequency
t7607c
0
15
30
45
60
75
90
Transfer
MT−2_1
MT−2_2
MT−4_1
MT−4_2

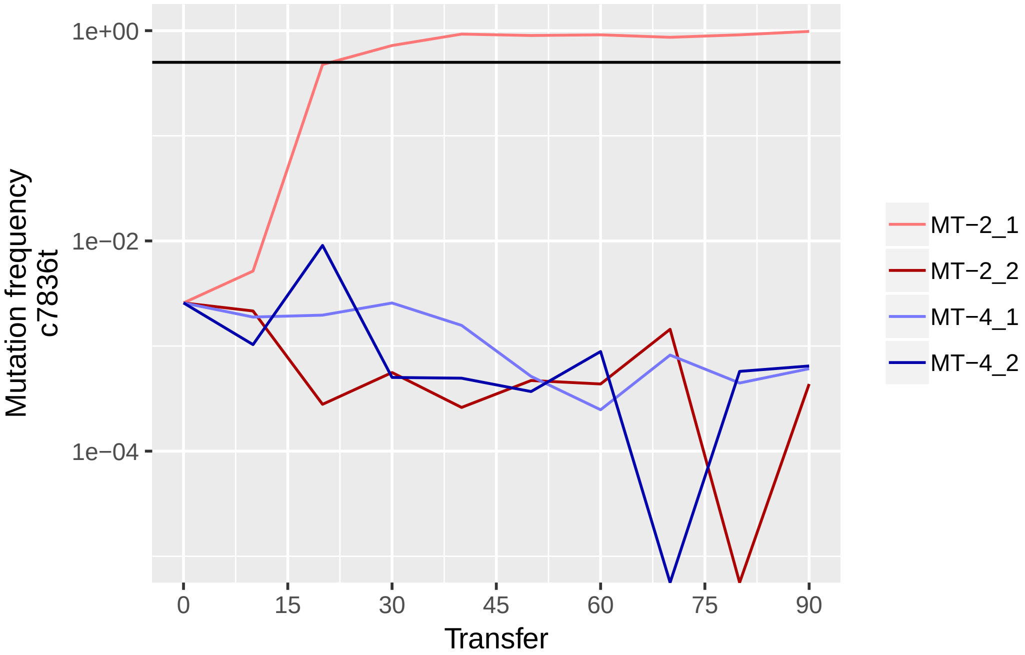

1e+00
1e−02
1e−04
Mutation frequency
c7836t
0
15
30
45
60
75
90
Transfer
MT−2_1
MT−2_2
MT−4_1
MT−4_2

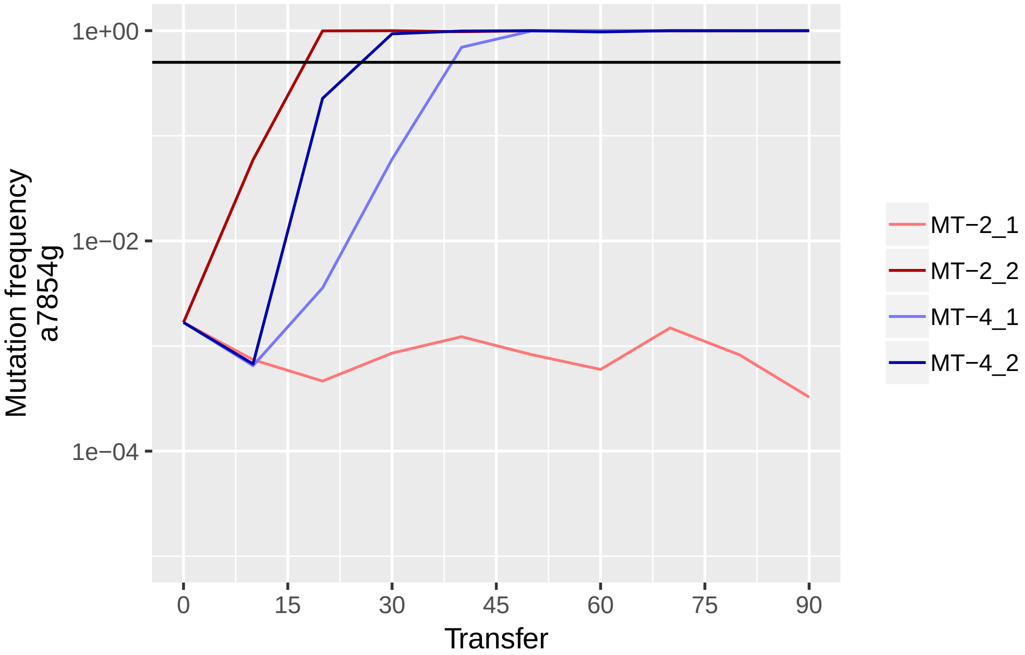

Mutation frequency
a7854g
1e+00
1e−02
1e−04
0
15
30
45
60
75
90
Transfer
MT−2_1
MT−2_2
MT−4_1
MT−4_2

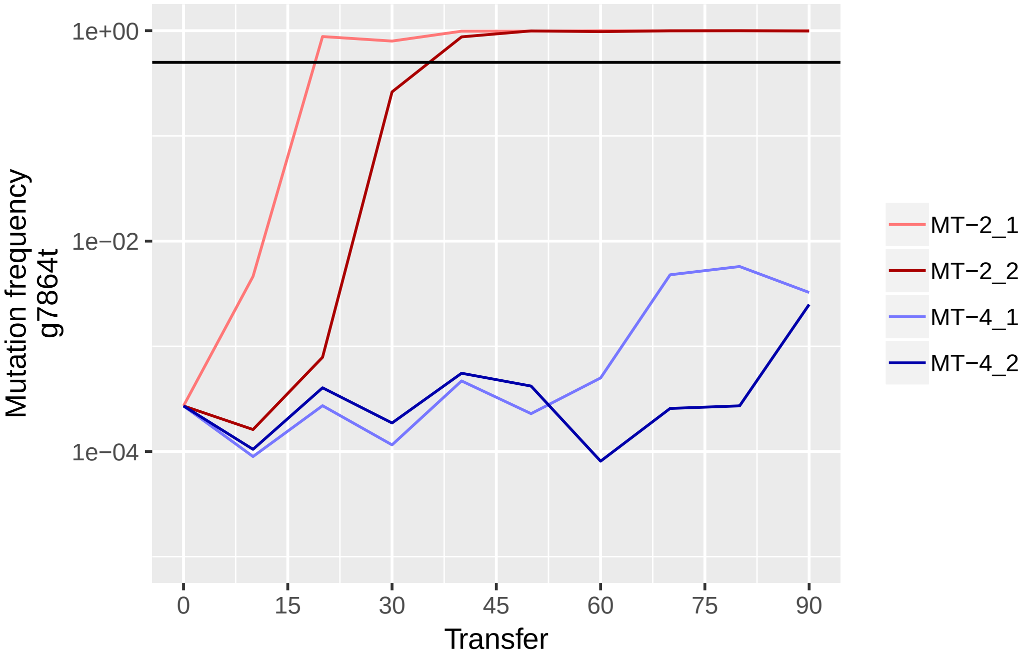

1e+00
1e−02
1e−04
Mutation frequency
g7864t
0
15
30
45
60
75
90
Transfer
MT−2_1
MT−2_2
MT−4_1
MT−4_2

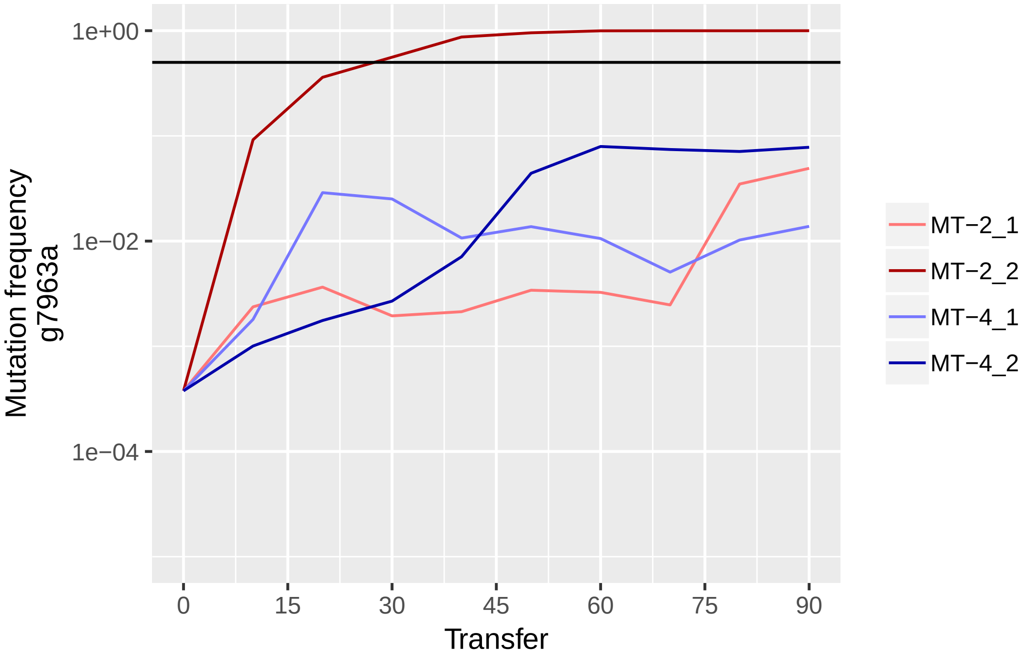
1e+00
1e−02
1e−04
Mutation frequency
g7963a
0
15
30
45
60
75
90
Transfer
MT−2_1
MT−2_2
MT−4_1
MT−4_2

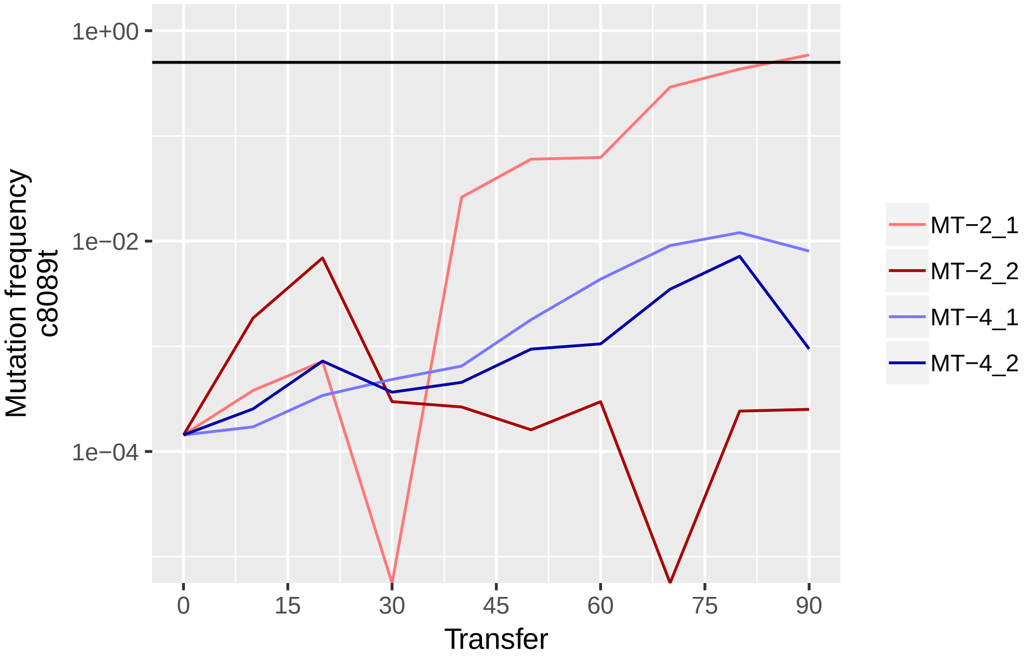

Mutation frequency
c8089t
1e+00
1e−02
1e−04
0
15
30
45
60
75
90
Transfer
MT−2_1
MT−2_2
MT−4_1
MT−4_2

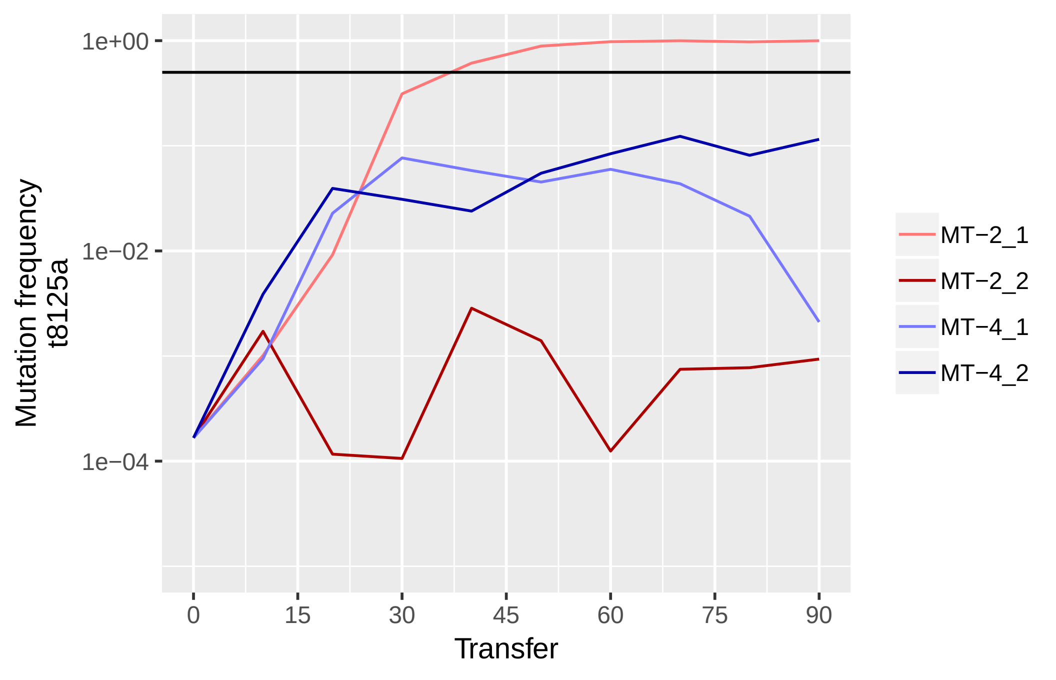

1e+00
1e−02
1e−04
Mutation frequency
t8125a
0
15
30
45
60
75
90
Transfer
MT−2_1
MT−2_2
MT−4_1
MT−4_2

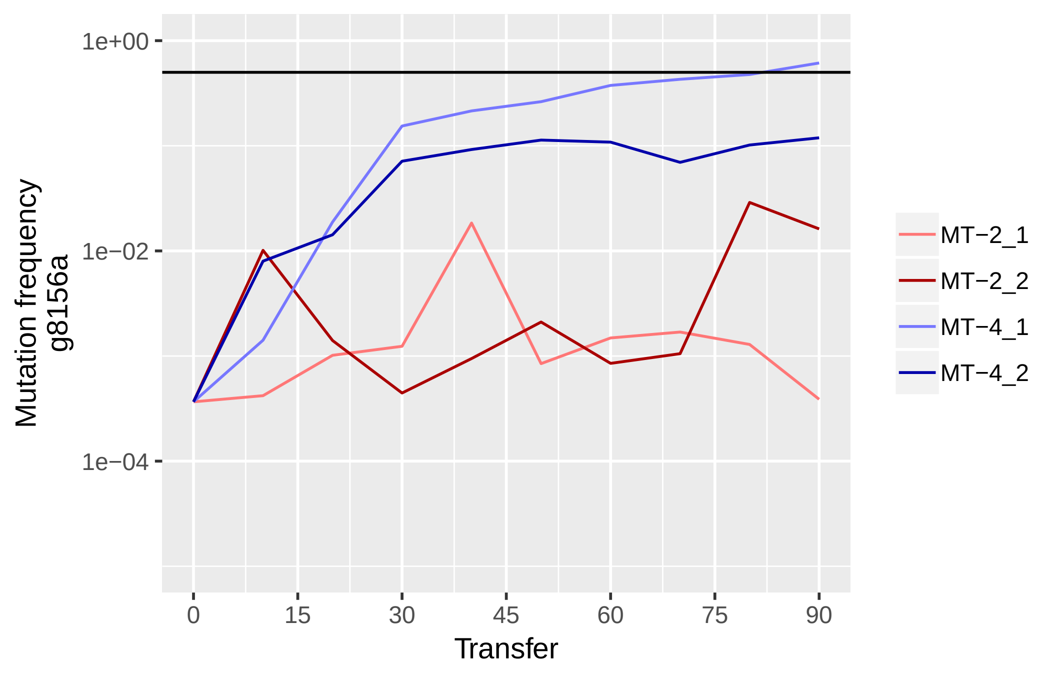

1e+00
1e−02
1e−04
Mutation frequency
g8156a
0
15
30
45
60
75
90
Transfer
MT−2_1
MT−2_2
MT−4_1
MT−4_2

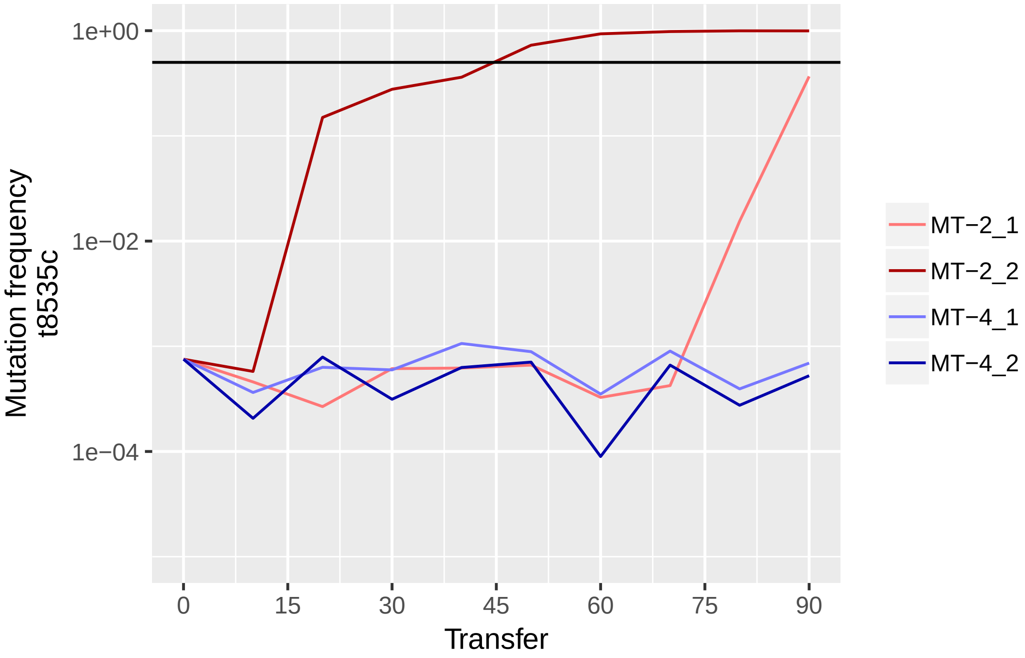

1e+00
1e−02
1e−04
Mutation frequency
t8535c
0
15
30
45
60
75
90
Transfer
MT−2_1
MT−2_2
MT−4_1
MT−4_2

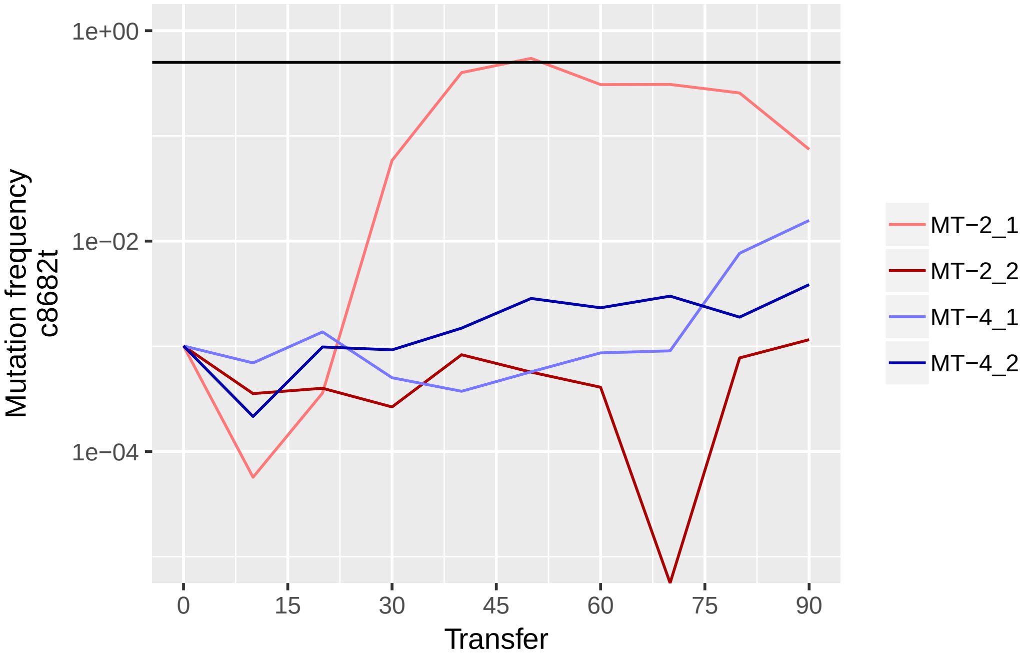

1e+00
1e−02
1e−04
Mutation frequency
c8682t
0
15
30
45
60
75
90
Transfer
MT−2_1
MT−2_2
MT−4_1
MT−4_2

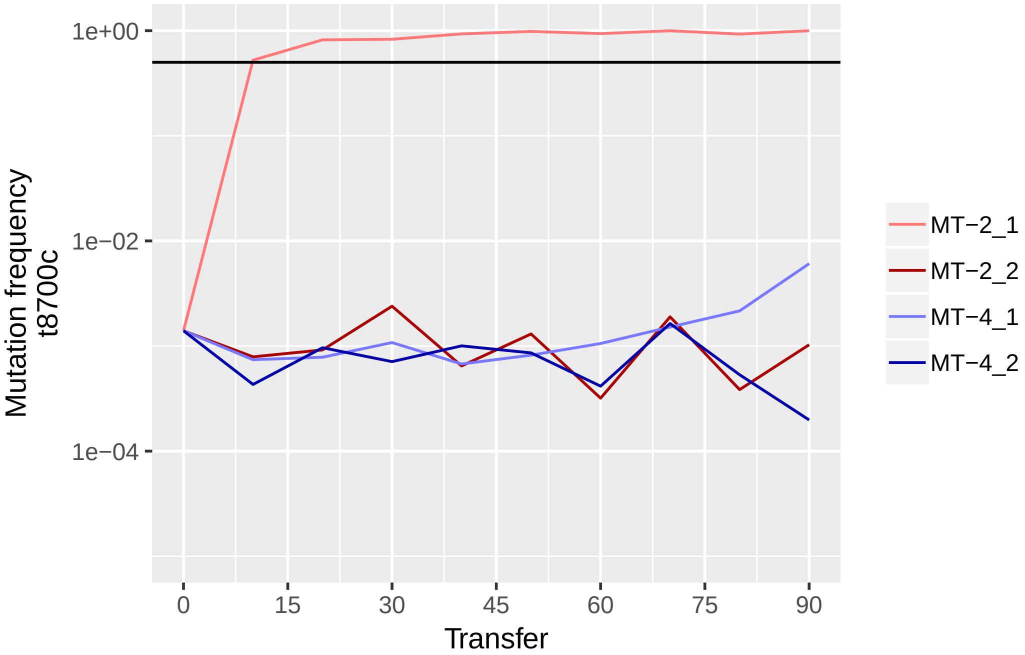

1e+00
1e−02
1e−04
Mutation frequency
t8700c
0
15
30
45
60
75
90
Transfer
MT−2_1
MT−2_2
MT−4_1
MT−4_2

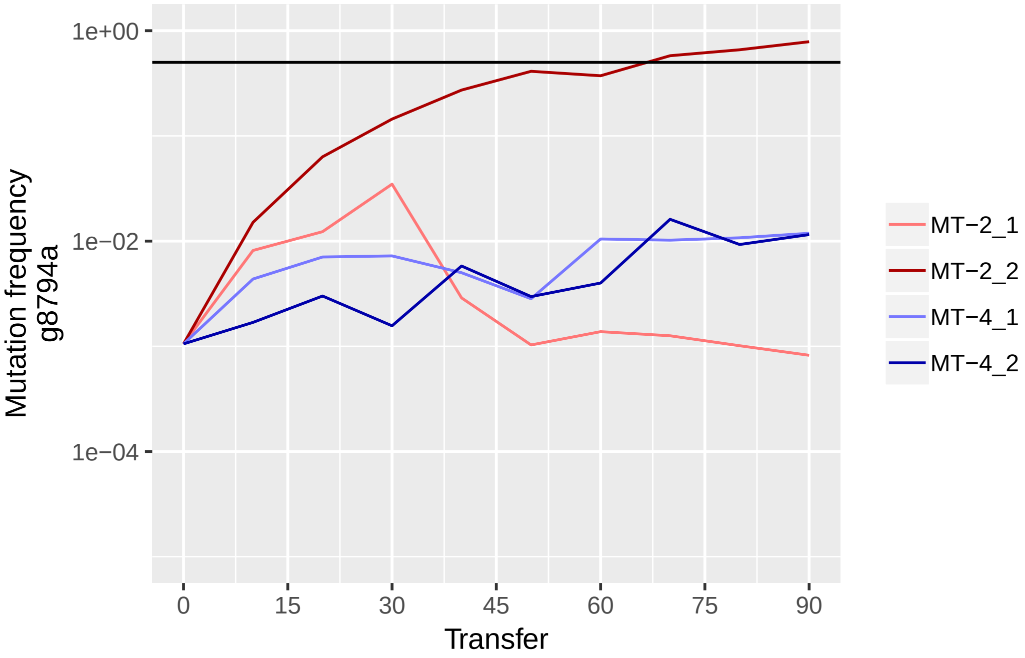

Mutation frequency
g8794a
1e+00
1e−02
1e−04
0
15
30
45
60
75
90
Transfer
MT−2_1
MT−2_2
MT−4_1
MT−4_2

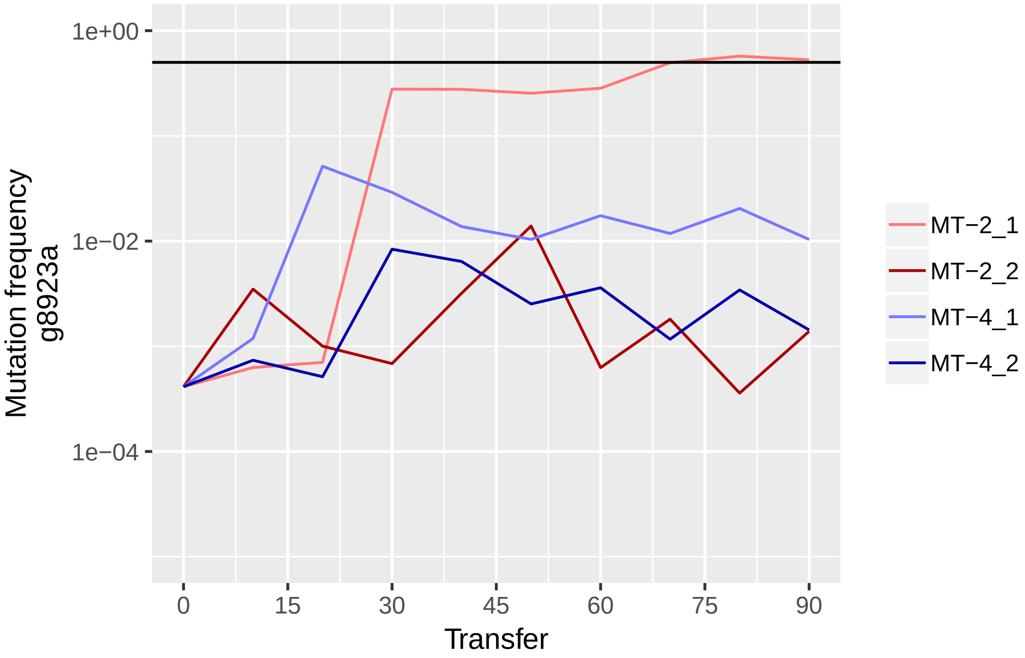
1e+00
1e−02
1e−04
Mutation frequency
g8923a
0
15
30
45
60
75
90
Transfer
MT−2_1
MT−2_2
MT−4_1
MT−4_2

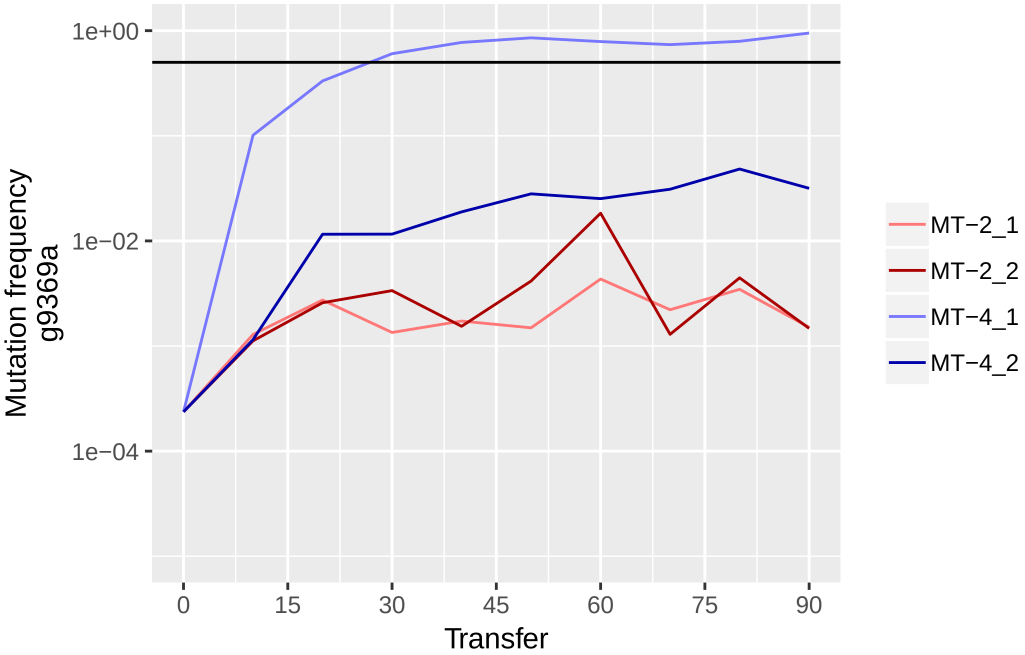
1e+00
1e−02
1e−04
Mutation frequency
g9369a
0
15
30
45
60
75
90
Transfer
MT−2_1
MT−2_2
MT−4_1
MT−4_2

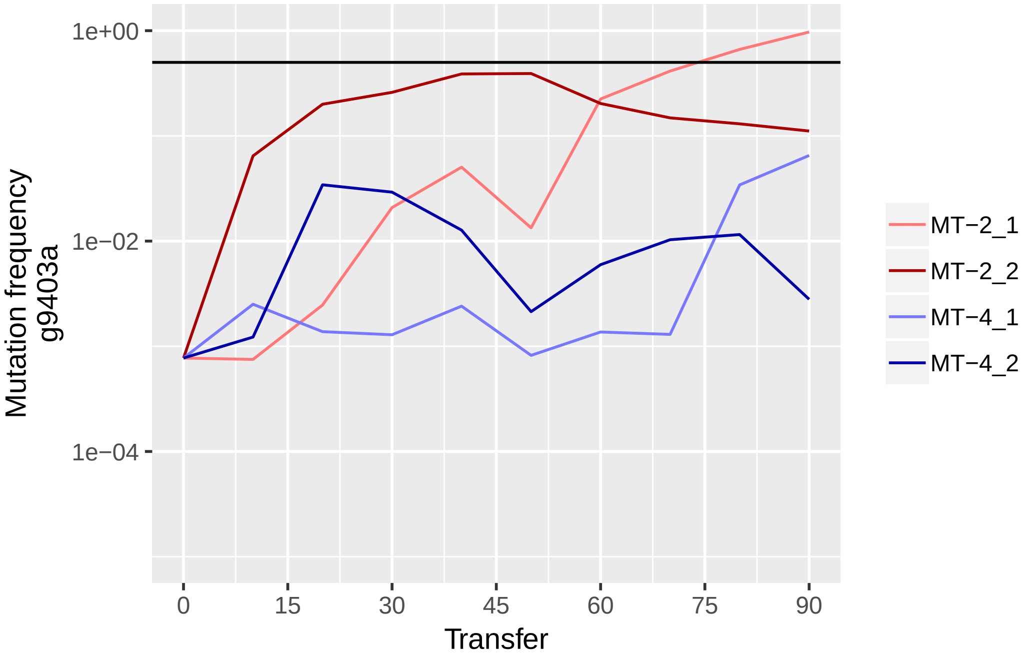

Mutation frequency
g9403a
1e+00
1e−02
1e−04
0
15
30
45
60
75
90
Transfer
MT−2_1
MT−2_2
MT−4_1
MT−4_2

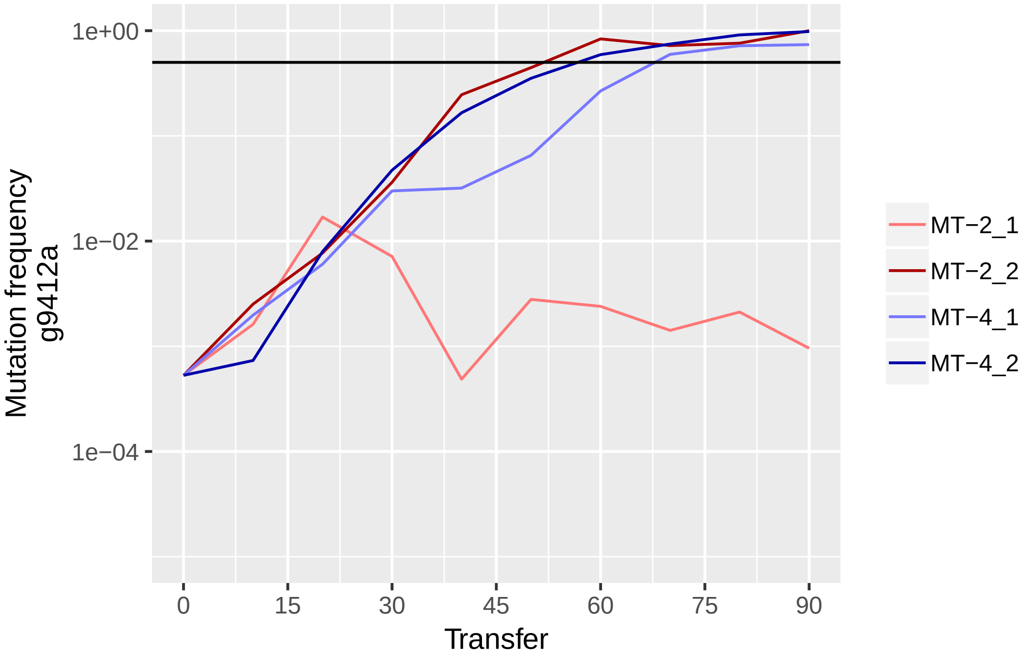

Mutation frequency
g9412a
1e+00
1e−02
1e−04
0
15
30
45
60
75
90
Transfer
MT−2_1
MT−2_2
MT−4_1
MT−4_2

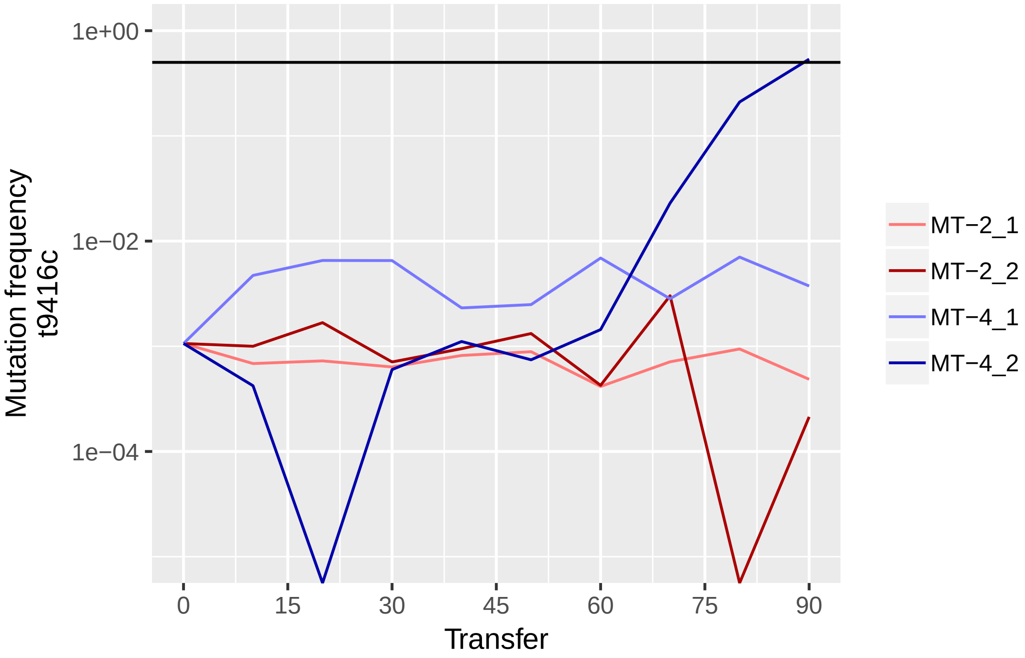

1e+00
1e−02
1e−04
Mutation frequency
t9416c
0
15
30
45
60
75
90
Transfer
MT−2_1
MT−2_2
MT−4_1
MT−4_2

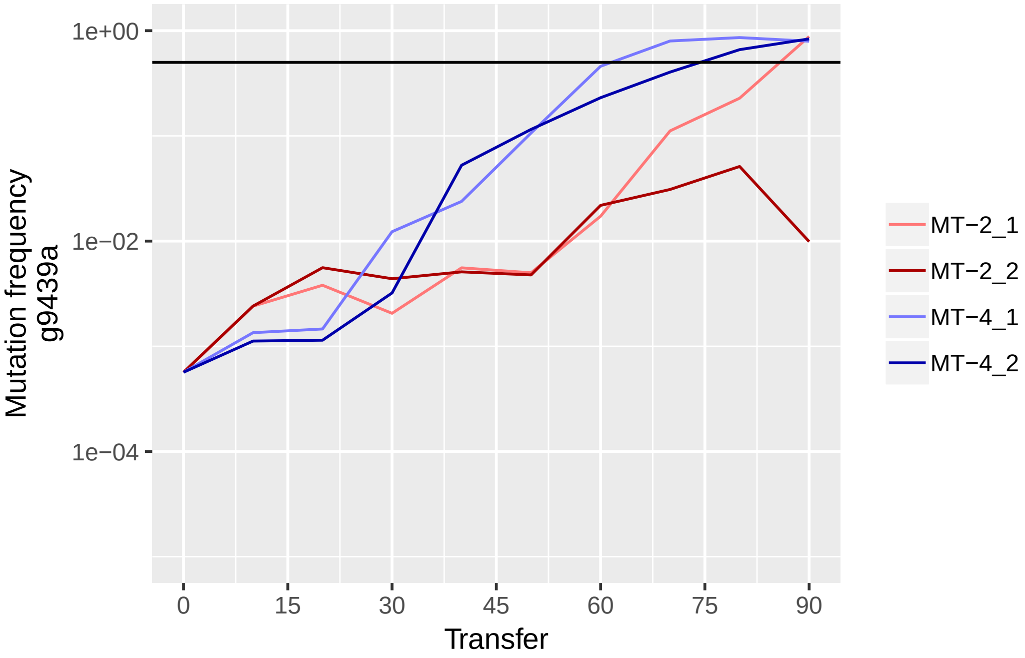

Mutation frequency
g9439a
1e+00
1e−02
1e−04
0
15
30
45
60
75
90
Transfer
MT−2_1
MT−2_2
MT−4_1
MT−4_2

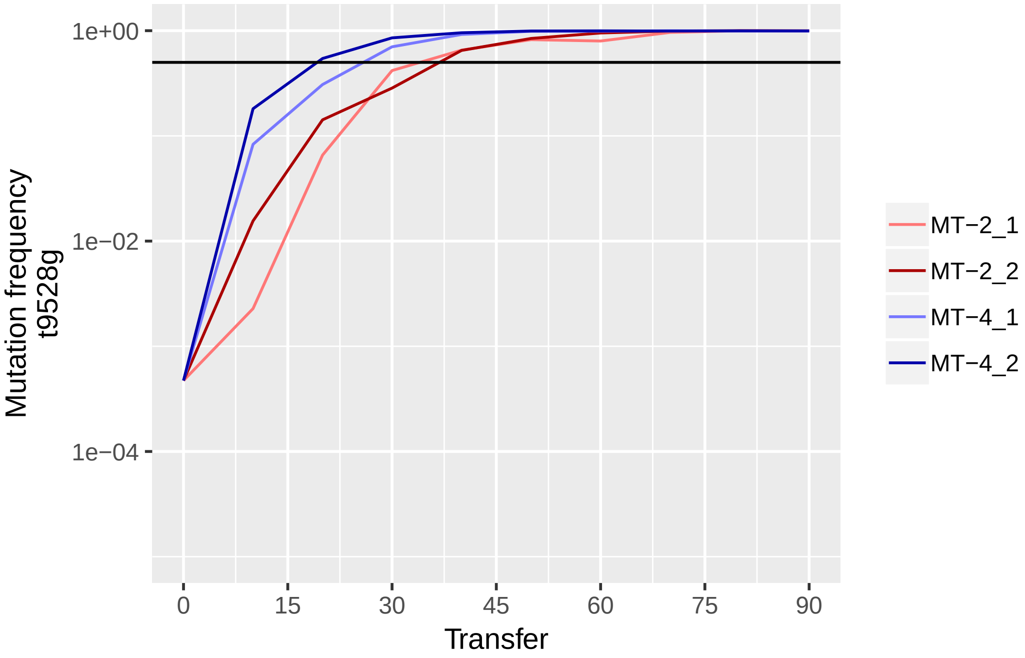

Mutation frequency
t9528g
1e+00
1e−02
1e−04
0
15
30
45
60
75
90
Transfer
MT−2_1
MT−2_2
MT−4_1
MT−4_2

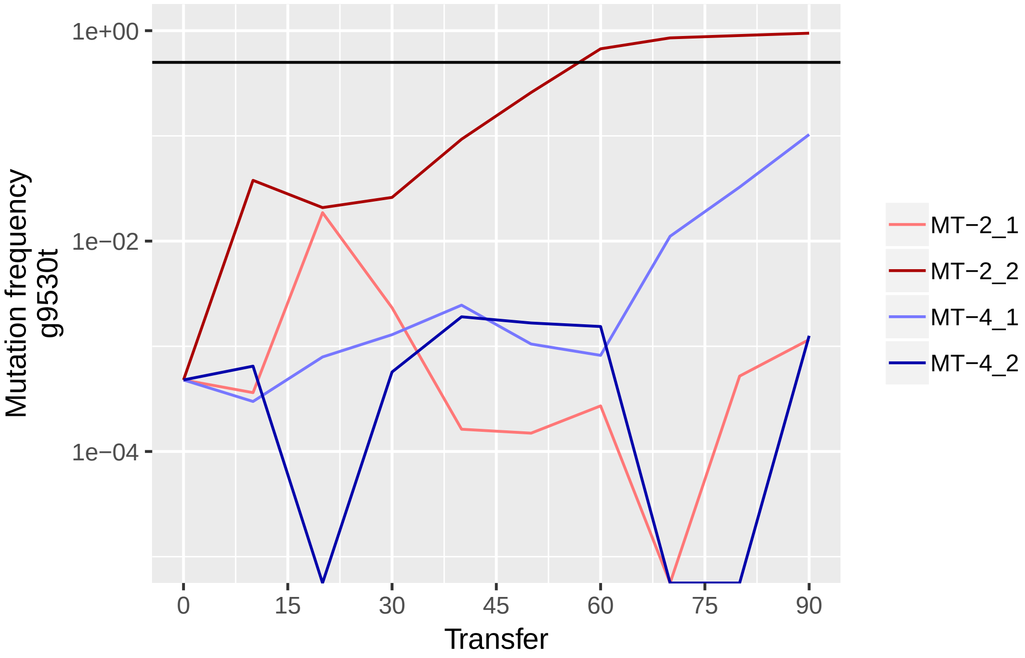

1e+00
1e−02
1e−04
Mutation frequency
g9530t
0
15
30
45
60
75
90
Transfer
MT−2_1
MT−2_2
MT−4_1
MT−4_2

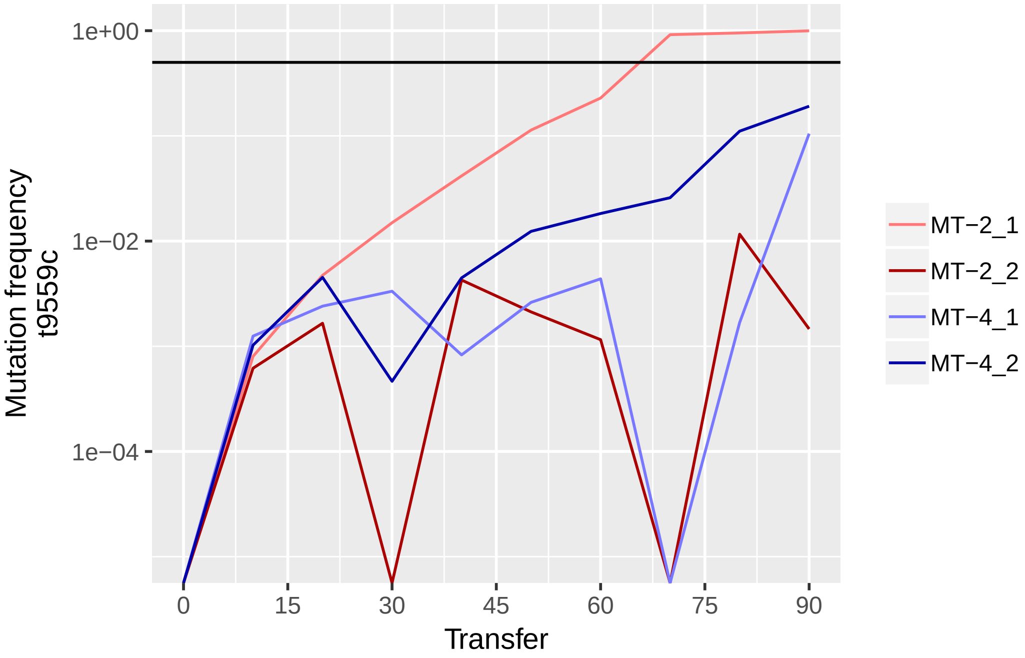
1e+00
1e−02
1e−04
Mutation frequency
t9559c
0
15
30
45
60
75
90
Transfer
MT−2_1
MT−2_2
MT−4_1
MT−4_2
