# Supplementary material for: Parallel Evolution of HIV-1 in a Long-Term Experiment
Source: Mol Biol Evol. 2019 Jul 4;36(11):2400–14. doi: 10.1093/molbev/msz155 (PMC6805227; doi:10.1093/molbev/msz155)
Supplement: msz155_Supplementary_Data [file msz155_supplementary_data.zip › Table_S1.pdf]

Table S1. Majority mutation frequencies observed at transfer 90.

DNA mutation	MT-2_1	MT-2_2	MT-4_1	MT-4_2	Mutation Type	Gene:AA mutation
**g566a**	0	0.97	0.01	0.04	U	
**t569a**	1	0.02	0	0	U	
**c604a**	0.93	0.03	0.61	0.99	U	
**c605a**	0.02	0.94	0.01	0	U	
**t609g**	0.54	0.91	0	0	U	
**c614a**	0.43	0	0.01	0.78	U	
**c614t**	0.01	0.93	0	0.19	U	
**g616a**	0.02	0.28	0.57	0.02	U	
**g618a**	0.96	0.55	0.2	0.99	U	
**g621a**	0.62	0	0.01	0.01	U	
**g656a**	0	0.79	0.05	0	U	
**a670g**	0	0	0	0.88	U	
**g678a**	0.07	0	0.55	0.94	U	
**g823a**	0.01	0.01	0.91	0.99	N	GAG:E12K
**g892a**	0	0	0.6	0.55	N	GAG:V35I
**g909a**	0	0	0.15	0.6	S	GAG:E40E
**g1110a**	0.59	0	0.01	0.03	S	GAG:E107E
**c1167t**	1	0	0	0	S	GAG:S126S
**g1410a**	0	0	0.05	0.63	S	GAG:E207E
**t1442c**	0	1	0	0	N	GAG:V218A
**t1446g**	1	1	0	0	N	GAG:H219Q
**a2000t**	0.57	0	0.01	0	N	GAG:N404I
**g2058a**	0	1	1	0.95	N	GAG:M423I
**g3078a**	1	0	0	0.01	N	RT:D177N
**g3158a**	0.52	0	0.01	0.01	S	RT:E203E
**c3317a**	0	0.9	0	0	N	RT:D256E
**g3581a**	0	0.76	0.03	0	S	RT:E344E
**g3624a**	0	0.05	0.62	0	N	RT:G359S
**c3951t**	0	0	0.86	0	N	Rnase:P468S
**g4451a**	0	0.58	0	0.06	S	INT:L74L
**t4607g**	0.55	0	0	0	S	INT:V126V
**g4808a**	0	0.03	0.41	0.63	S	INT:G193G
**t4866c**	0.88	0	0	0.01	S	INT:L213L
**g4970a**	1	0	0.01	0.12	S	INT:G247G
**c5419a**	0	0.95	0	0	N	VIF:R127S
**t5465c**	0	0	0.56	0	N	VIF:V142A
**g5730a**	0	0.72	0.04	0.04	N	VPR:E58K
**g5803a**	0	0	0.58	0	N	VPR:G82D
**g5823a**	0.61	0	0.01	0	N	VPR:A89T
**g6018a**	0	0.6	0.01	0.01	S	TAT:Q63Q REV:R17K

**g6123a**	0.72	0	0.02	0	S	VPU:V21V
**g6603a**	0	0	0.99	0.01	N	ENV:S128N
**g6705a**	0	0.89	0	0	N	ENV:S162N
**g6783a**	0	0	1	0.96	N	ENV:S190N
**c6807a**	0	1	0	0	N	ENV:T198N
**c6909a**	0.66	0	0	0.01	N	ENV:T232K
**t7383-gtttaatagt acttg**	0.65	0.05	0.19	0.18	In-frame deletion	
**g7407a**	0.01	0.73	0	0	N	ENV:S398N
**a7529g**	0.83	0	0	0	N	ENV:I439V
**t7607c**	0.01	0.01	0.8	0	N	ENV:S465P
**c7836t**	0.98	0	0	0	N	ENV:A541V
**a7854g**	0	1	1	1	N	ENV:D547G
**g7864t**	1	0.99	0	0	N	ENV:Q550H
**g7963a**	0.05	1	0.01	0.08	S	ENV:V583V
**c8089t**	0.59	0	0.01	0	S	ENV:N625N
**t8125a**	1	0	0	0.12	N	ENV:N637K
**g8156a**	0	0.02	0.61	0.12	N	ENV:E648K
**t8535c**	0.37	1	0	0	N	ENV:L774S
**t8700c**	1	0	0.01	0	N	ENV:V829A
**g8794a**	0	0.79	0.01	0.01	N	NEF:G3D
**g8923a**	0.53	0	0.01	0	N	NEF:S46N
**g9369a**	0	0	0.95	0.03	N	NEF:A195T
**g9403a**	0.97	0.11	0.07	0	N	NEF:C206Y
**g9412a**	0	1	0.74	0.98	U	
**t9416c**	0	0	0	0.54	U	
**g9439a**	0.88	0.01	0.79	0.84	U	
**t9528g**	1	1	0.99	1	U	
**g9530t**	0	0.95	0.1	0	U	
**t9559c**	1	0	0.11	0.19	U	
