# Supplementary material for: Parallel Evolution of HIV-1 in a Long-Term Experiment
Source: Mol Biol Evol. 2019 Jul 4;36(11):2400–14. doi: 10.1093/molbev/msz155 (PMC6805227; doi:10.1093/molbev/msz155)
Supplement: msz155_Supplementary_Data [file msz155_supplementary_data.zip › Table_S2.pdf]

**Table S2. Probability of positively selected codons inferred by CodeML.**

| AA change in HXB2 | Affected HIV-1 gene | Pos. in HXB2 | Parallelism | Posterior probability of positive selection | | |
|---|---|---|---|---|---|---|
| | | | | True evol. history | Inferred evol. history | Minority mut. |
| E12K | GAG | g823a | 2 | 0.975* | 0.971* | 0.978* |
| V35I | GAG | g892a | 2 | 0.976* | 0.972* | 0.979* |
| V218A | GAG | t1442c | 1 | 0.762 | 0.741 | 0.784 |
| H219Q | GAG | t1446g | 2 | 0.979* | 0.975* | 0.983* |
| N404I | GAG | a2000t | 1 | 0.754 | 0.733 | 0.774 |
| A407T | GAG | g2008a | 1 | 0.979* | 0.975* | 0.982* |
| M423I | GAG | g2058a | 3 | 0.998** | 0.997** | 0.998** |
| D177N | POL | g3078a | 1 | 0.761 | 0.740 | 0.783 |
| D256E | POL | c3317a | 1 | 0.759 | 0.738 | 0.780 |
| G359S | POL | g3624a | 1 | 0.757 | 0.736 | 0.778 |
| P623S | POL | c3951t | 1 | 0.773 | 0.753 | 0.797 |
| R127S | VIF | c5419a | 1 | 0.766 | 0.746 | 0.789 |
| V142A | VIF | t5465c | 1 | 0.741 | 0.720 | 0.759 |
| E58K | VPR | g5730a | 1 | 0.733 | 0.712 | 0.750 |
| G82D | VPR | g5803a | 1 | 0.765 | 0.744 | 0.982* |
| A89T | VPR | g5823a | 1 | 0.976* | 0.972* | 0.979* |
| S128N | ENV | g6607a | 1 | 0.753 | 0.733 | 0.774 |
| S142N | ENV | g6649a | 1 | 0.755 | 0.734 | 0.775 |
| S162N | ENV | g6709a | 1 | 0.762 | 0.742 | 0.784 |
| S190N | ENV | g6793a | 2 | 0.978* | 0.741 | 0.982* |
| T198N | ENV | c6817a | 1 | 0.766 | 0.745 | 0.788 |
| T232K | ENV | c6919a | 1 | 0.762 | 0.741 | 0.784 |
| S398N | ENV | g7417a | 1 | 0.754 | 0.733 | 0.981* |
| I439V | ENV | a7539g | 1 | 0.767 | 0.746 | 0.789 |
| S465P | ENV | t7617c | 1 | 0.773 | 0.753 | 0.797 |
| A541V | ENV | c7846t | 1 | 0.768 | 0.747 | 0.790 |
| D547G | ENV | a7864g | 3 | 0.998** | 0.997** | 0.998** |
| Q550H | ENV | g7874t | 2 | 0.998** | 0.975* | 0.982* |
| N637K | ENV | t8135a | 1 | 0.746 | 0.725 | 0.765 |
| E648K | ENV | g8166a | 1 | 0.733 | 0.712 | 0.750 |
| L774S | ENV | t8545c | 1 | 0.769 | 0.748 | 0.791 |
| A823V | ENV | c8692t | 1 | 0.760 | 0.739 | 0.781 |
| V829A | ENV | t8710c | 1 | 0.760 | 0.739 | 0.781 |
| G3D | NEF | g8804a | 1 | 0.765 | 0.745 | 0.787 |
| S46N | NEF | g8933a | 1 | 0.763 | 0.742 | 0.785 |
| A195T | NEF | g9379a | 1 | 0.768 | 0.747 | 0.790 |
| C206Y | NEF | g9413a | 1 | 0.772 | 0.751 | 0.795 |

**Green** shading indicates parallel majority mutations.
**Red** mutations have been falsely classified as positively selected due to changes in mutation frequency across the experiment (i.e., the mutations appear and disappear as majority mutations).

**The yellow** mutation has been misclassified as not positively selected because of the wrong topology of the inferred phylogenetic tree because it emerges just before the two MT-4 lines separate in the inferred phylogeny.

**Blue** mutations have been misclassified because of:

(1) The wrong tree topology of the minority mutation tree (g7417a) (For g7417a it is incorrectly inferred because the MT-2_2 90 is incorrectly clustered outside (before) MT-2_2 70 and 80, hence the mutation appeared twice in the minority mutation tree. (2)

(2) A change in mutation frequency that led to the disappearance and reappearance of the mutation g8503a. In theory this should also lead to a problem for the two other trees, this is however not the case. In the true tree this is not a problem because the branch lengths of MT-4_1 50 and MT-4_1 60 are of zero length (disappearance is of no consequence). It also is not a problem for the inferred phylogeny because there the topology is wrong, MT-4_1 50 is clustered outside (before) MT-4_1 60.
