# Supplementary figures and images for: Parallel Evolution of HIV-1 in a Long-Term Experiment
Source: Mol Biol Evol. 2019 Jul 4;36(11):2400–14. doi: 10.1093/molbev/msz155 (PMC6805227; doi:10.1093/molbev/msz155)

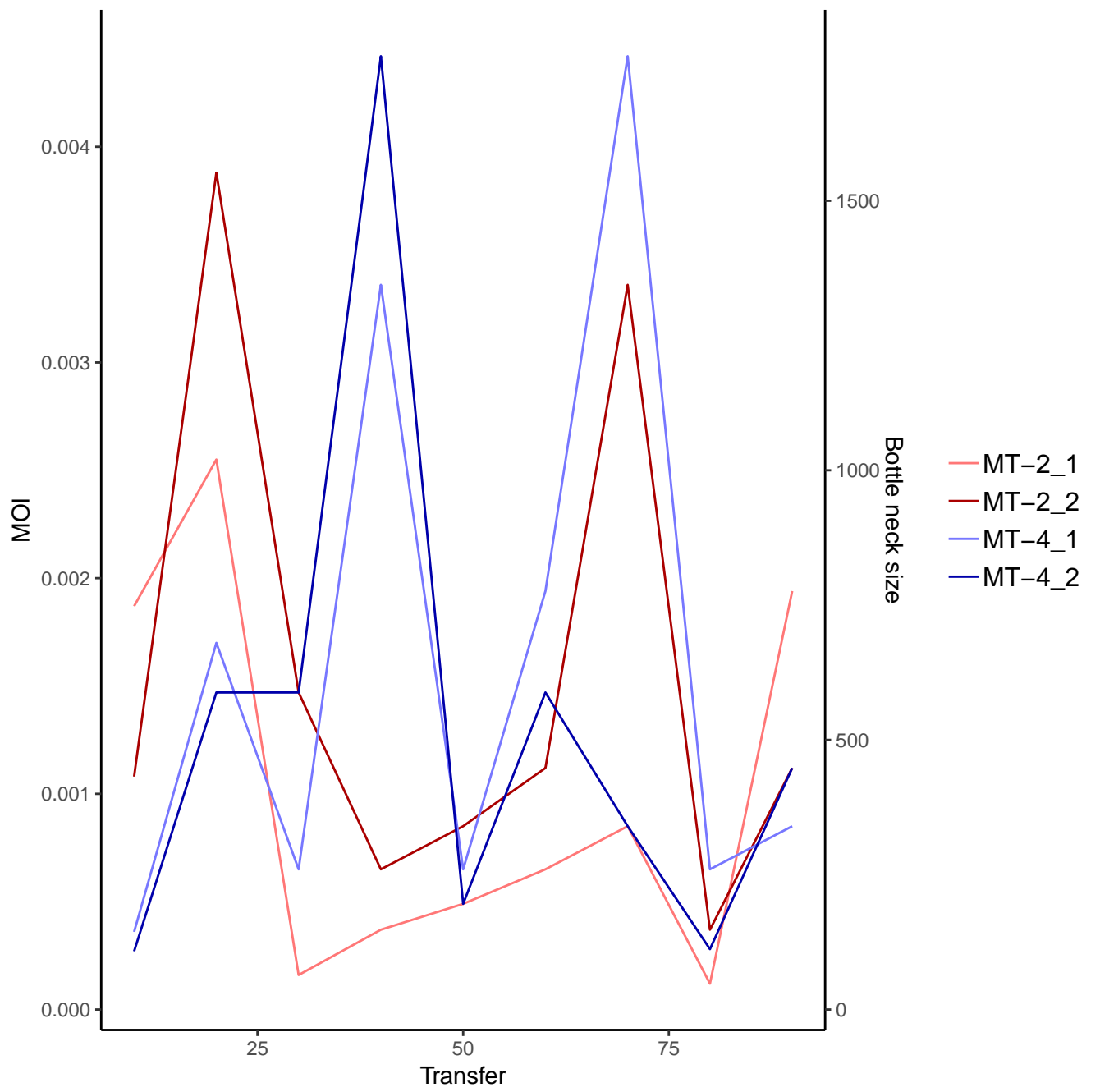

Supplement: msz155_Supplementary_Data [file msz155_supplementary_data.zip › Figure_S1.pdf]

MT-2\_1

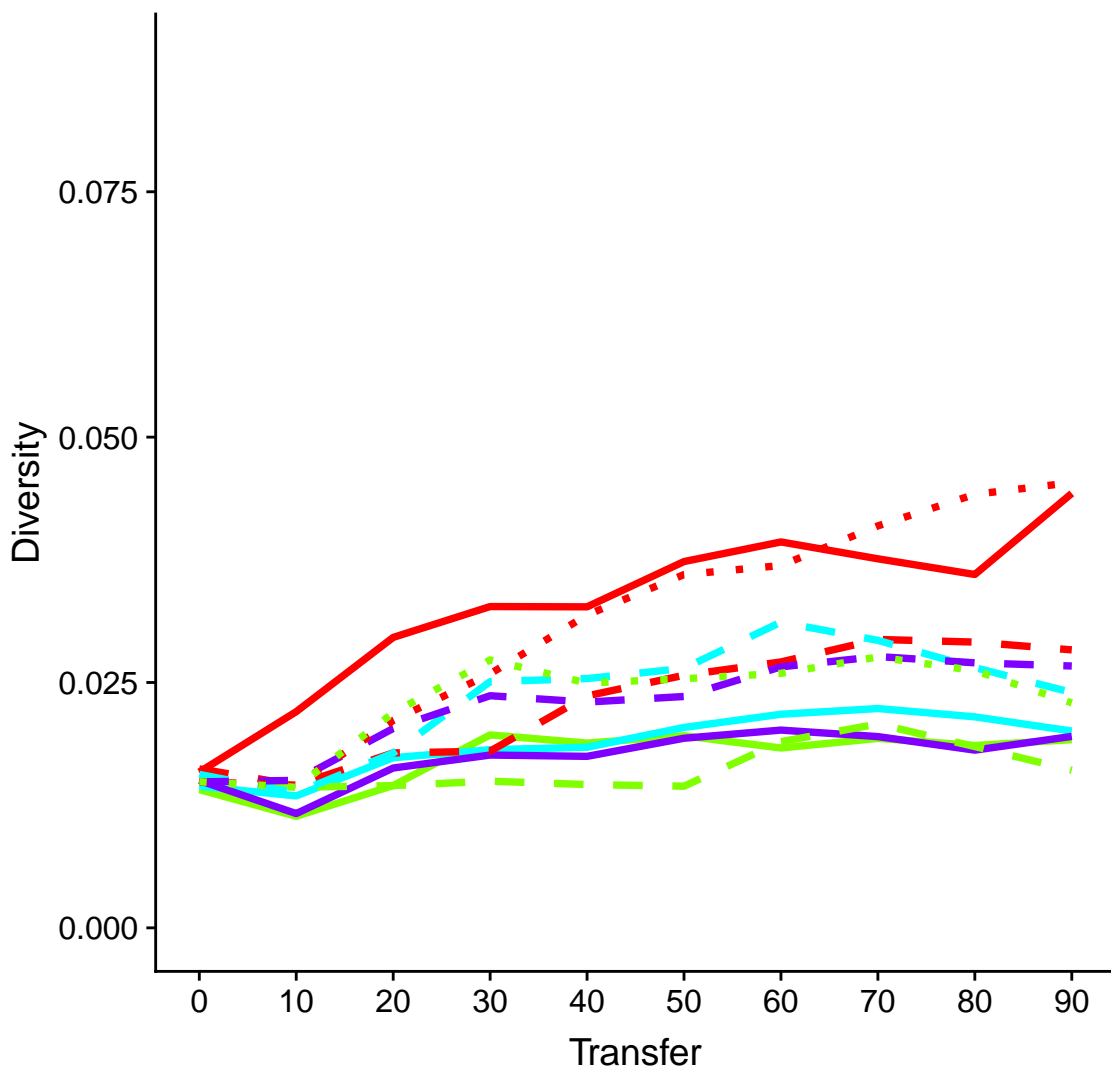

MT-2\_2

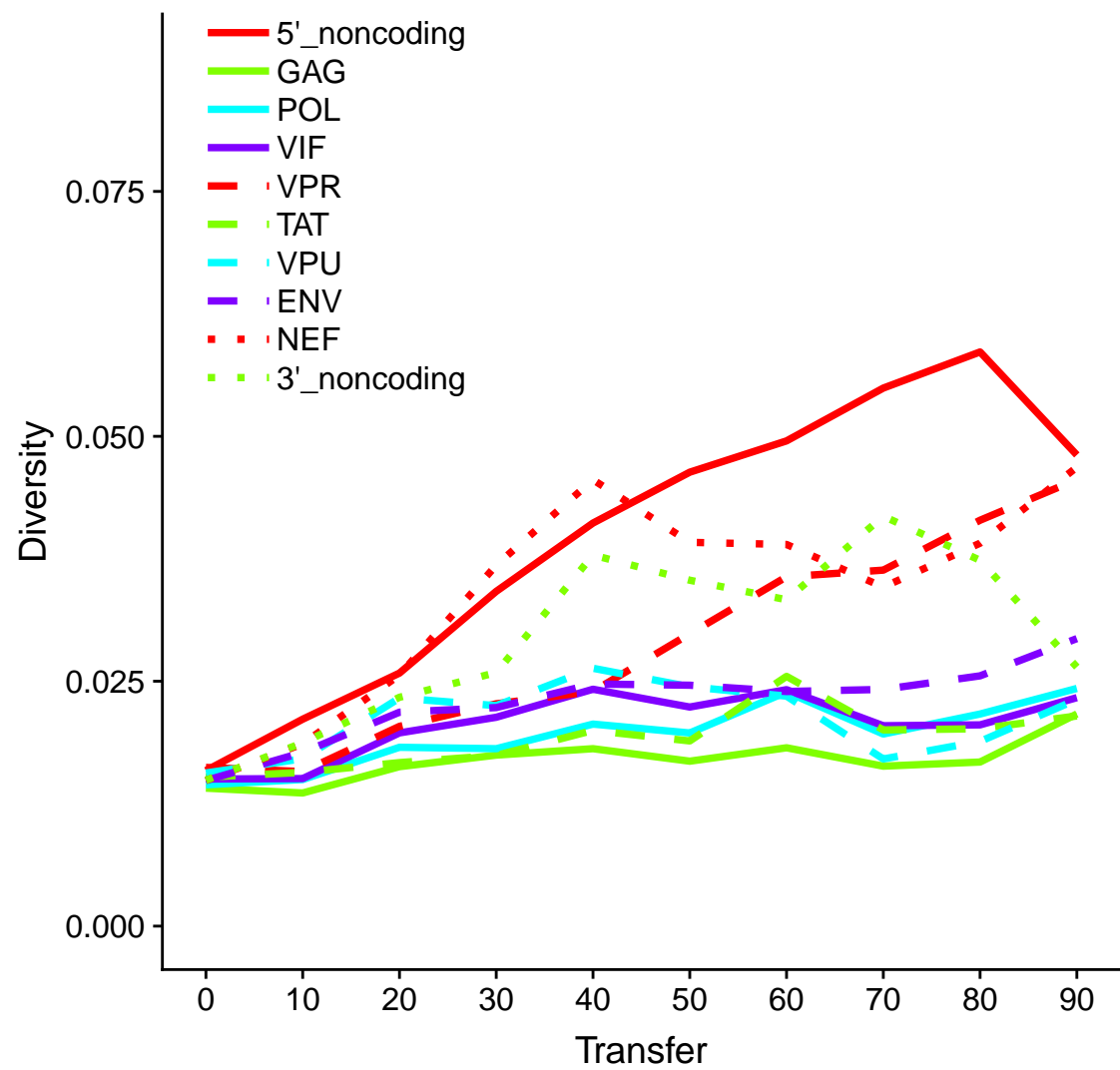

MT-4\_1

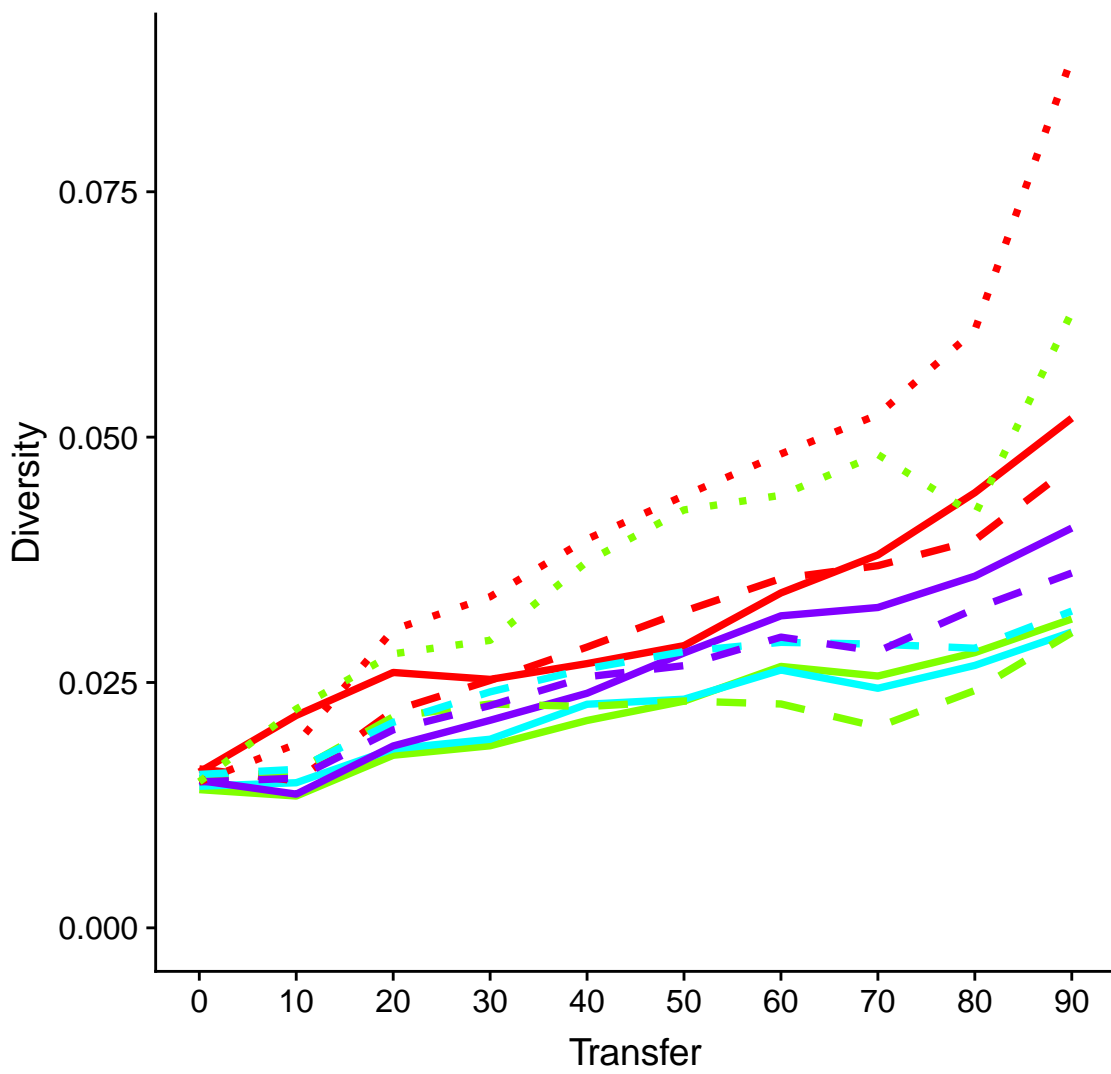

MT-4\_2

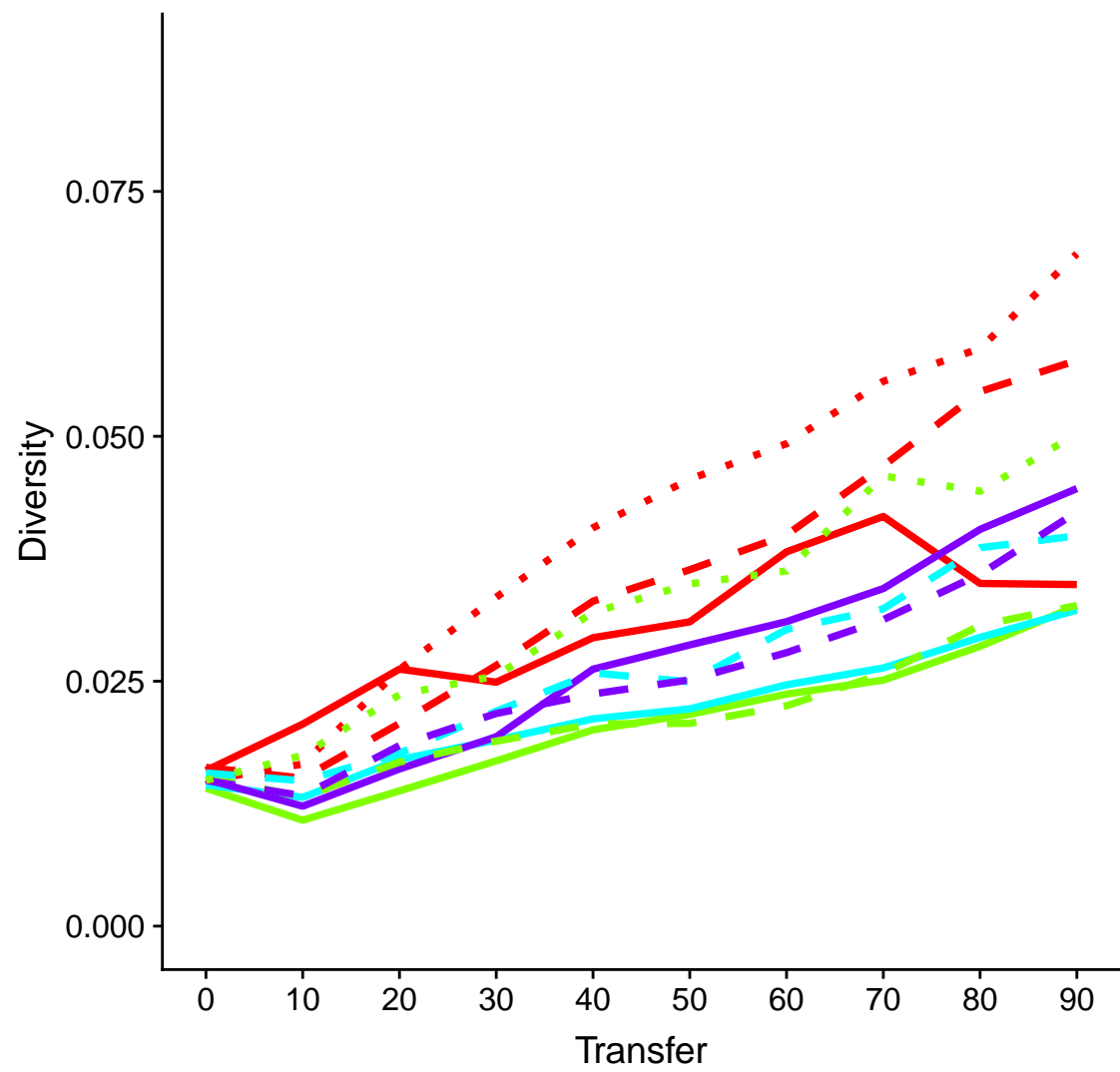

Supplement: msz155_Supplementary_Data [file msz155_supplementary_data.zip › Figure_S3.pdf]

MT-2\_1

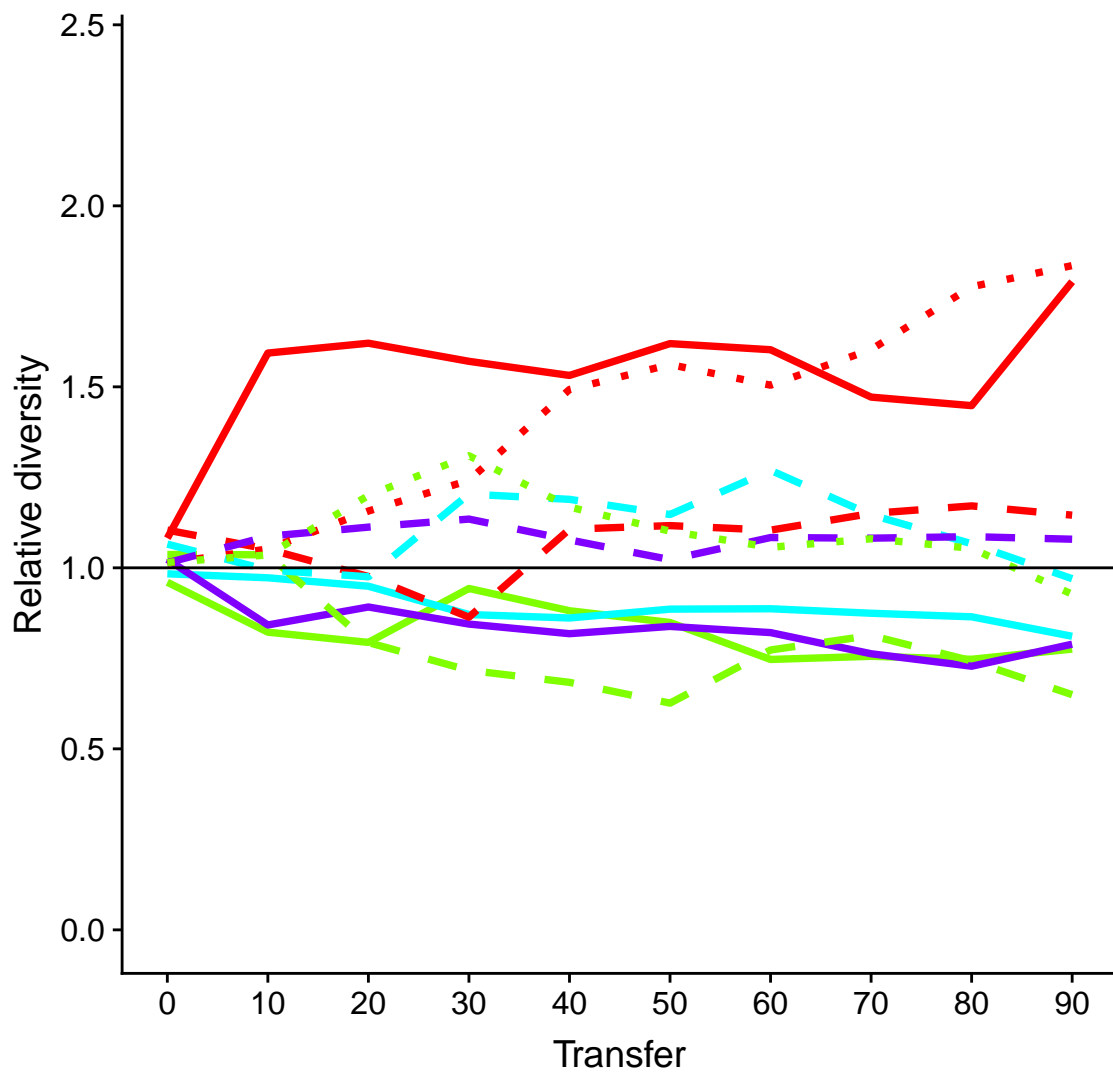

MT-2\_2

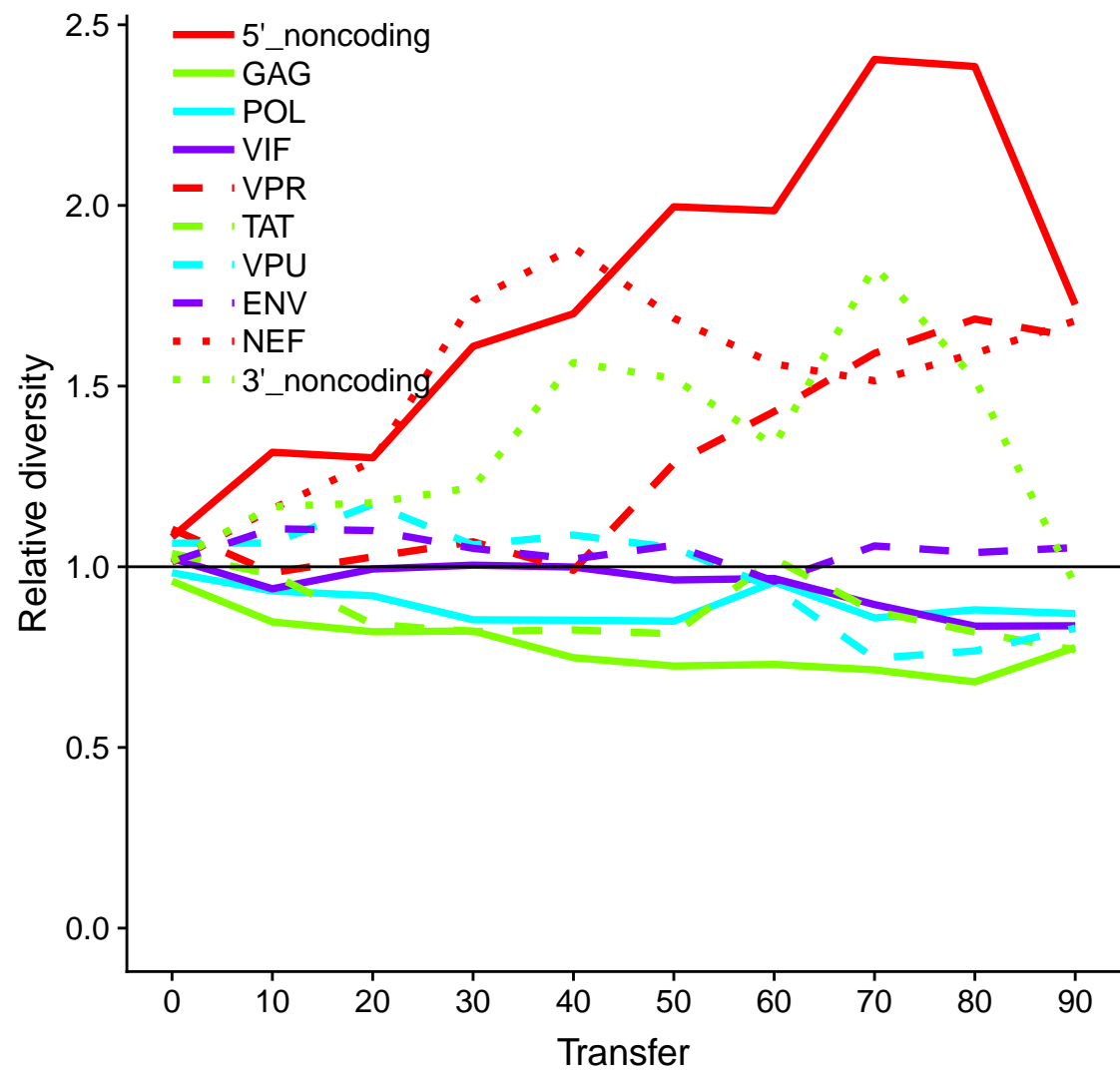

MT-4\_1

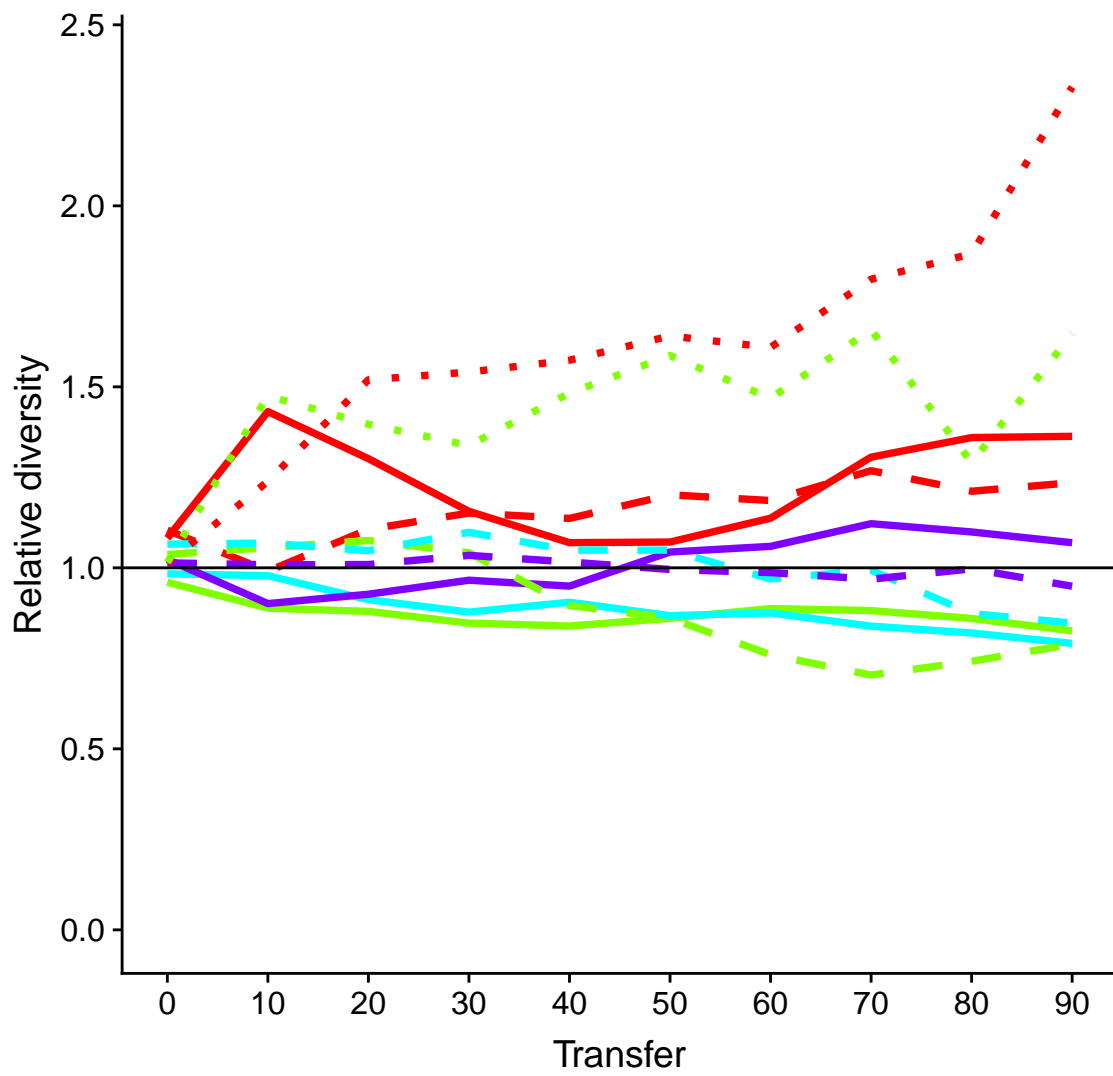

MT-4\_2

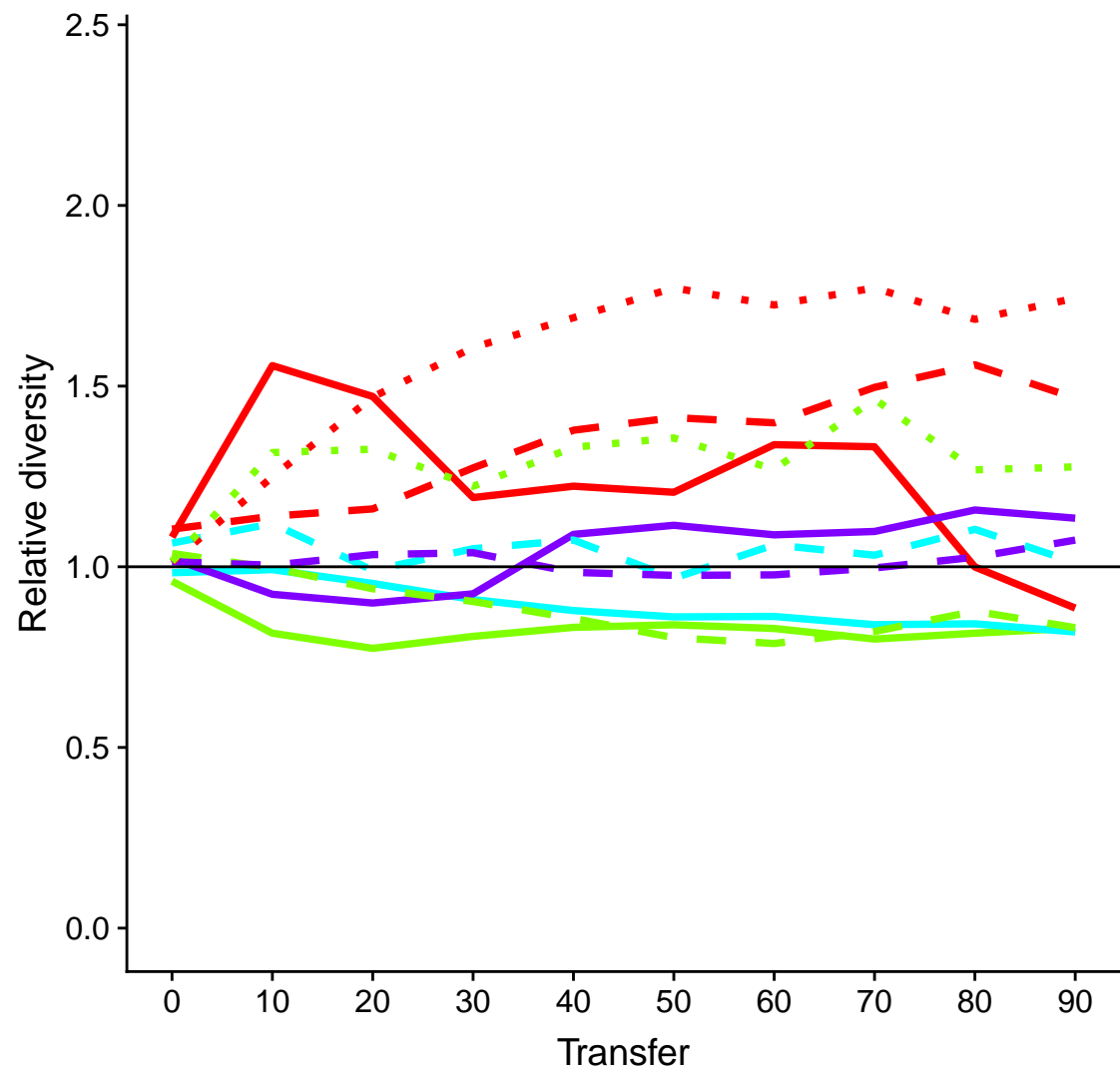

Supplement: msz155_Supplementary_Data [file msz155_supplementary_data.zip › Figure_S4.pdf]

## NGS Sample coverage

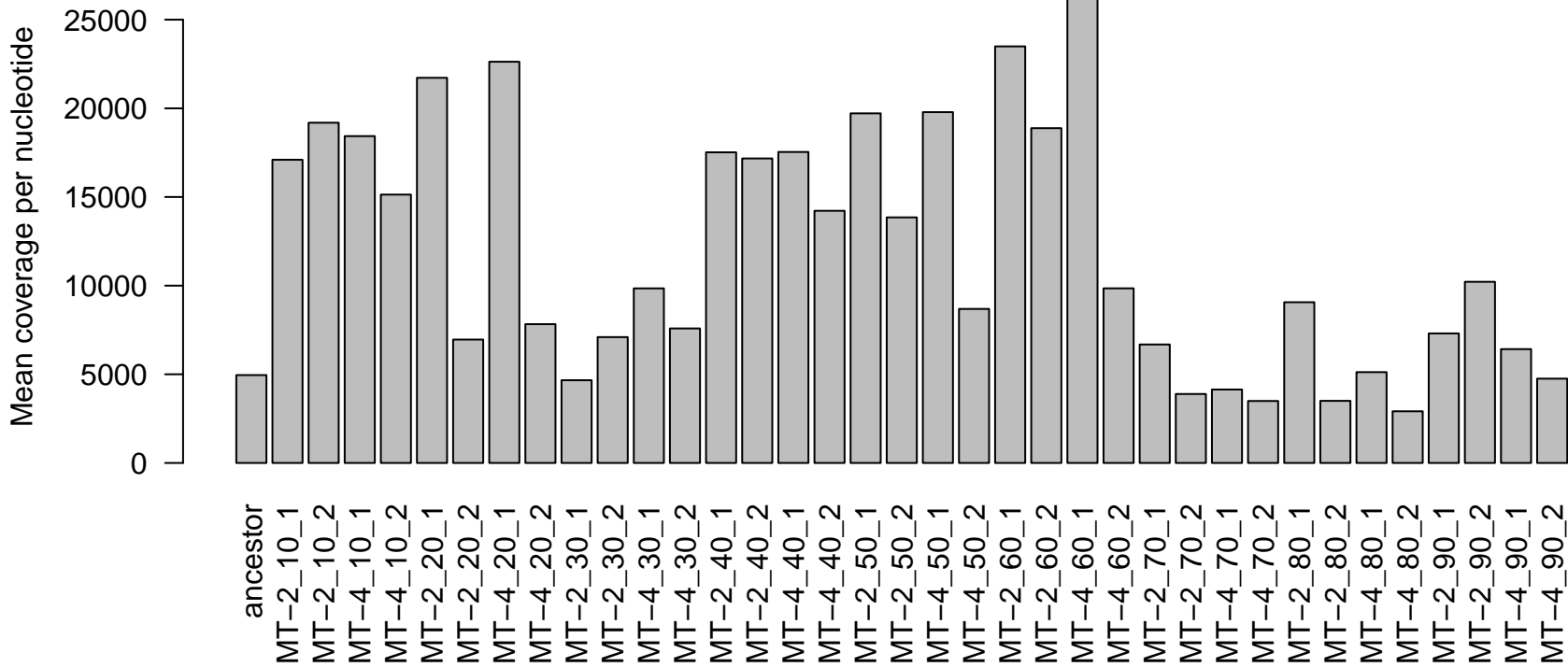

Supplement: msz155_Supplementary_Data [file msz155_supplementary_data.zip › Figure_S5.pdf]
